# Structure–Activity Relationships and Antiplasmodial Potencies of Novel 3,4-Disubstituted 1,2,5-Oxadiazoles

**DOI:** 10.3390/ijms241914480

**Published:** 2023-09-23

**Authors:** Patrick Hochegger, Theresa Hermann, Johanna Dolensky, Werner Seebacher, Robert Saf, Eva-Maria Pferschy-Wenzig, Marcel Kaiser, Pascal Mäser, Robert Weis

**Affiliations:** 1 Institute of Pharmaceutical Sciences, Pharmaceutical Chemistry, University of Graz, Schubertstraße 1, A-8010 Graz, Austria; patrick.hochegger@yahoo.de (P.H.); johanna.faist@uni-graz.at (J.D.); we.seebacher@uni-graz.at (W.S.); robert.weis@uni-graz.at (R.W.); 2 Institute for Chemistry and Technology of Materials (ICTM), Graz University of Technology, Stremayrgasse 9, A-8010 Graz, Austria; robert.saf@tugraz.at; 3 Institute of Pharmaceutical Sciences, Pharmacognosy, University of Graz, Beethovenstraße 8, A-8010 Graz, Austria; eva-maria.wenzig@uni-graz.at; 4 Swiss Tropical and Public Health Institute, Kreuzstraße 2, CH-4123 Allschwil, Switzerland; marcel.kaiser@swisstph.ch (M.K.); pascal.maeser@swisstph.ch (P.M.); 5 Faculty of Philosophy and Natural Sciences, University of Basel, Swiss TPH, Petersplatz 1, CH-4003 Basel, Switzerland

**Keywords:** 1,2,5-oxadiazoles, antimalarial, *Plasmodium falciparum*, CYP3A4-inhibition, solubility

## Abstract

The 4-substituted 3-amino-1,2,5-oxadiazole **1** from the Malaria Box Project of the Medicines for Malaria Venture foundation shows very promising selectivity and in vitro activity against *Plasmodium falciparum*. Within the first series of new compounds, various 3-acylamino analogs were prepared. This paper now focuses on the investigation of the importance of the aromatic substituent in ring position 4. A number of new structure–activity relationships were elaborated, showing that antiplasmodial activity and selectivity strongly depend on the substitution pattern of the 4-phenyl moiety. In addition, physicochemical parameters relevant for drug development were calculated (logP and ligand efficiency) or determined experimentally (CYP3A4-inhibition and aqueous solubility). *N*-[4-(3-ethoxy-4-methoxyphenyl)-1,2,5-oxadiazol-3-yl]-3-methylbenzamide **51** showed high in vitro activity against the chloroquine-sensitive strain NF54 of *P. falciparum* (*Pf*NF54 IC_50_ = 0.034 µM), resulting in a very promising selectivity index of 1526.

## 1. Introduction

Malaria unfortunately continues to be globally the most prevalent tropical disease, predominantly caused by *Plasmodium falciparum*. Despite enormous efforts in malaria eradication, almost half of the world’s population is still at risk of infection. According to the WHO’s latest *Malaria Report* from 2022, an estimated 247 million malaria cases and more than 600,000 deaths worldwide were counted in the year 2021 [1,2]. Although nowadays there are options for treatment, the COVID-19 pandemic brought progress in the fight against malaria largely to a halt. Negligence of aid and prevention programs due to a focus on COVID-19 resulted in a noticeable increase in malaria infections over the last few years [3]. And as if that is not enough, resistance development against all available drugs also has been rapidly increasing, resulting in a real threat of untreatable infections [4]. In that regard, the main problem is the resistance development against artemisinin and artemisinin-based combination therapies (ACTs) (the gold standard in malaria therapy) in the WHO African region. Most frequent mutations are reported in *Pf*kelch13 and *Pf*mdr1 [5,6,7]. The development of an effective malaria vaccine remains an enormous challenge, mostly due to the multiple possible targets. Only very few vaccine candidates have shown promising and especially long-term efficacy [8]. Therefore, triple artemisinin-based combination therapies (TACTs) are a last-ditch attempt to at least delay drug resistant malaria until new, especially orally applicable, antimalarials become available [9,10,11].

**Figure 1:**
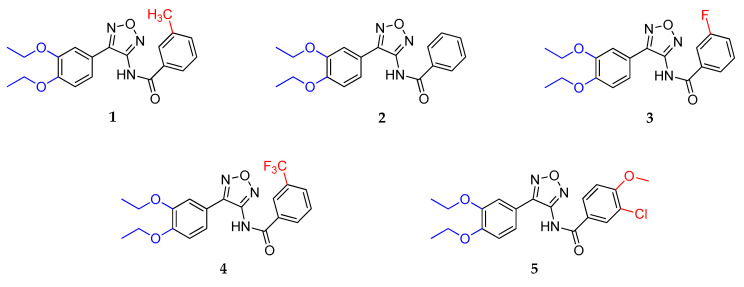
Structure–activity relationships of the lead compound **1** and promising compounds from the first series of derivatives [14].

To support open-source drug development for novel antimalarials, the foundation Medicines for Malaria Venture published data of a huge screening project, the so-called Malaria Box Project, in 2016. It contains 400 drug- and probe-like compounds with various activities against *Plasmodium* spp. [12,13]. Within our first study, we reported the synthetic preparation, first derivatizations and antiplasmodial activities of the 4-substituted 3-amino-1,2,5-oxadiazole **1**. We focused on synthesizing compounds with variously substituted *N*-acyl moieties in position 3 of the 1,2,5-oxadiazole. Thereby, a series of structure–activity relationships were elaborated. The most active compounds were 3-substituted or 3,4-disubstituted benzamides (Figure 1) [14]. This paper now focuses on the effect of different substitutions of the 4-aryl moiety of **1**. This way, we were able to gain further insights into structure–activity relationships.

## 2. Results and Discussion

### 2.1. Chemistry

**Figure 2:**
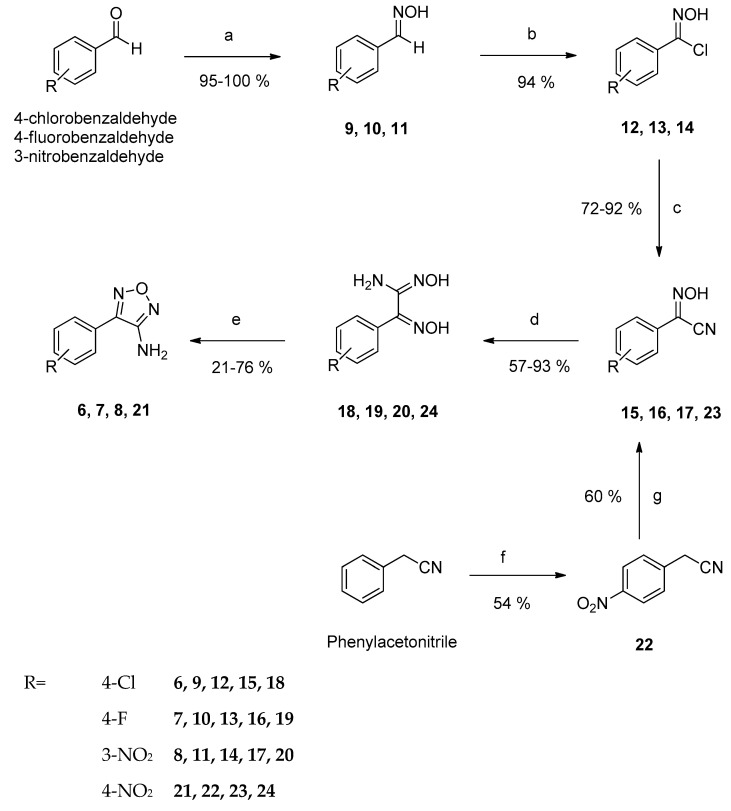
Preparation of compounds **6, 7**, **8** and **21**. Reagents and conditions: (a) NH_2_OH x HCl, NaHCO_3_, water, methanol, 100 °C, 2–3 h; (b) *N*-chlorosuccinimide, dry dimethylformamide, rt, 1–24 h; (c) potassium cyanide, diethyl ether, water, rt, 24 h or potassium cyanide, ethyl acetate, water, 5–10 °C, 30 min; (d) NH_2_OH × HCl, NaHCO_3_, water, methanol, 100 °C, 24 h; (e) 2*N* NaOH, 120 °C, 24 h or anhydrous sodium acetate, dry ethanol, 100 °C, 120–192 h; (f) H_2_SO_4_ 98%, HNO_3_ 67%, rt, 1 h; (g) Na, dry ethanol, isoamyl nitrite, rt, 24 h.

New derivatives of the lead structure **1** were prepared by synthesizing variously 4-substituted 3-amino-1,2,5-oxadiazoles via three different synthetic routes. Preparation of the 4-amino-1,2,5-oxadiazoles **6**, **7** and **8** started by treating the corresponding benzaldehydes with hydroxylamine hydrochloride, obtaining the respective aldoximes **9**, **10** and **11** in excellent yields. To improve the reactivity of the aldoximes, imidoyl chlorides **12**, **13** and **14** were prepared by subsequent reaction with *N*-chlorosuccinimide. These intermediates were treated in situ with an excess of potassium cyanide, giving the (hydroxyimino) acetonitriles **15**, **16** and **17**. The second to last step was the preparation of the amide oximes 18, 19 and 20 by the reaction of the cyano group of **15**, **16** and **17** with hydroxylamine hydrochloride. Refluxing **18**, **19** and **20** in 2*N* NaOH overnight gave the desired 3- and 4-substituted 3-aminofurazans **6**, **7** and **8** by intramolecular ring closure [15,16,17]. For the preparation of the 3-amino-4-(4-nitrophenyl)-1,2,5-oxadiazole **21**, a slightly different reaction scheme starting from phenylacetonitrile was applied. The latter was treated with a mixture of nitric and sulfuric acid, yielding its 4-nitro derivative **22** [18]. The α-position of the acetonitrile was deprotonated with sodium ethylate. The formed anion gave with isoamyl nitrite hydroxyimino derivative **23** in moderate yields [19]. Nitrile **23** was refluxed with hydroxylamine hydrochloride and sodium bicarbonate, yielding the amide oxime **24**. Finally, reaction with 2*N* NaOH gave the desired furazan **21** (Figure 2) [15,17].

**Figure 3:**
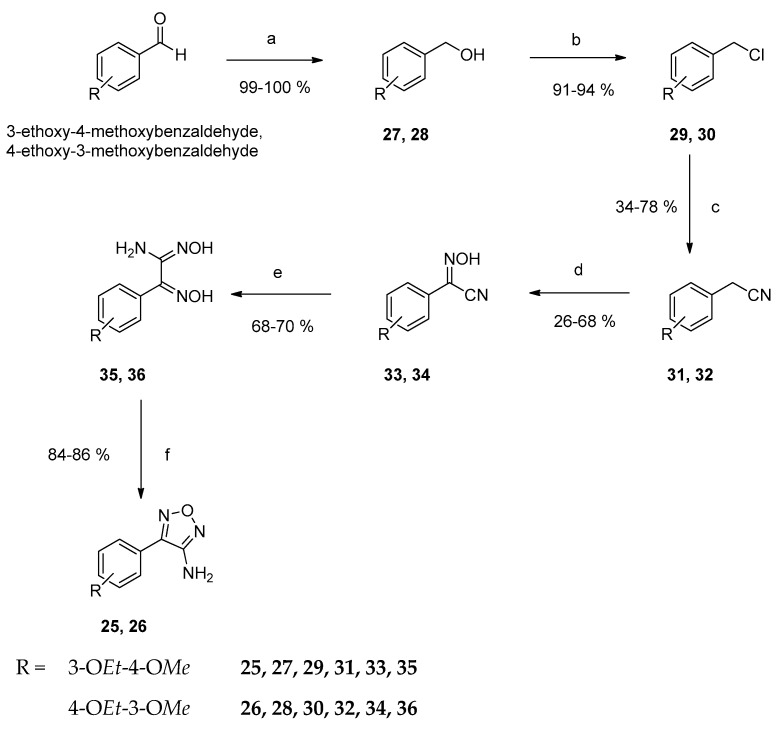
Preparation of compounds **25** and **26**. Reagents and conditions: (a) NaBH_4_, dry methanol, rt, 1 h; (b) (1) dry dichloromethane, 0 °C; (2) thionyl chloride, rt, 24 h; (c) (1) dry dimethylformamide, potassium cyanide, 100 °C, 2 h; (2) potassium cyanide, 100 °C, 2 h; (d) Na, dry ethanol, isoamyl nitrite, rt, 24 h; (e) NH_2_OH × HCl, NaHCO_3_, water, methanol, 100 °C, 24 h; (f) 2*N* NaOH, 120 °C, 24 h.

Starting from commercially available 3-ethoxy-4-methoxybenzaldehyde and 4-ethoxy-3-methoxybenzaldehyde, respectively, the corresponding 4-substituted 3-aminofurazans **25** and **26** were prepared in a 6-step synthesis. Firstly, the aldehydes were reduced to the respective primary alcohols **27** and **28** by treatment with sodium borohydride in methanol. The successful reduction was indicated by the disappearance of the carbonyl resonance in the ^13^C NMR spectrum. A signal for the new methylene carbon became visible at 65 ppm. Nucleophilic substitution with thionyl chloride in dry dichloromethane yielded the alkyl chlorides **29** and **30**. Thus, the resonance of the methylene carbon was shifted 17 ppm to higher field. The nitriles **31** and **32** were obtained by a Kolbe nitrile synthesis with an excess of potassium cyanide in dimethylformamide [20]. The resonance of the methylene carbon was shifted 23 ppm to lower frequencies and a new signal for the carbon atom of the cyano group appeared at 118 ppm. The nitriles were converted to their 2-hydroxyimino derivatives **33** and **34** as outlined above **[19]**. The resonance of the imino carbon appeared at 131 ppm, whereas the signal of the cyano group was shifted 8 ppm to higher field. The amide oximes **35** and **36** were prepared by refluxing **33** and **34** with hydroxylamine hydrochloride and sodium bicarbonate in water and methanol. The conversion of the cyano group to an amide oxime shifted the signal of the carbon of the pre-existing oxime 17 ppm downfield. The resonance of the carbon of the amidine carbon appeared at 147 ppm. Subsequent intramolecular ring closure to the desired 4-(3-ethoxy-4-methoxyphenyl)- and 4-(4-ethoxy-3-methoxyphenyl)-furazans **25** and **26** was achieved by reaction with 2*N* NaOH (Figure 3) [15]. Successful ring formation was detected by a 0.5 ppm downfield shift of the resonance of the amino protons in the ^1^H NMR spectrum, whereas the resonance of the corresponding carbon atom was shifted 6 ppm to higher frequencies in the ^13^C NMR spectrum.

**Figure 4:**
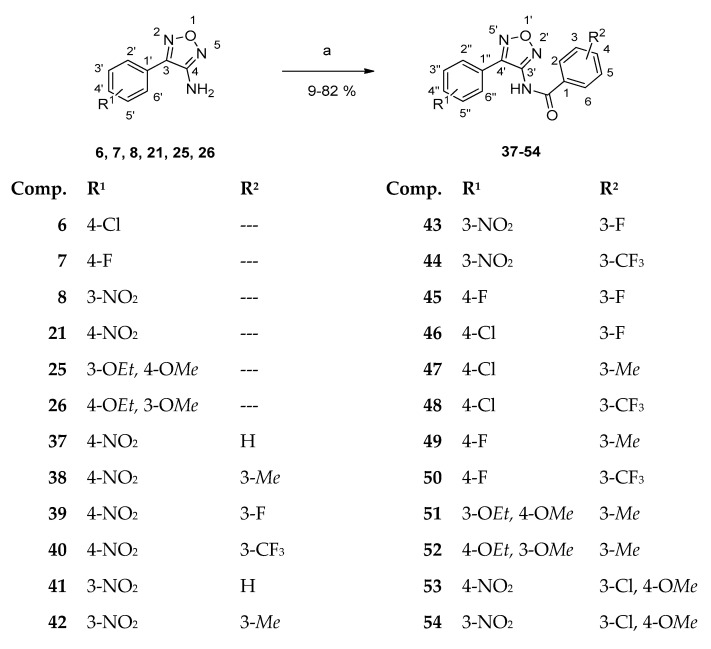
Preparation of compounds **37–54**. Reagents and conditions: (a) (1) NaH, dry dimethylformamide, aminofurazan, 0 °C, 20 min; (2) benzoyl chloride, dry dimethylformamide, 60 °C, 24 h (compounds **37** and **41**); or (1) NaH, dry dimethylformamide, aminofurazan, 0 °C, 20 min; (2) *m*-toluoyl chloride, dry dimethylformamide, 60 °C, 24–48 h (compounds **38, 42, 47, 49, 51** and **52**); or (1) NaH, dry dimethylformamide, aminofurazan, 0 °C, 20 min; (2) 3-fluorobenzoyl chloride, dry dimethylformamide, 60 °C, 24 h (compounds **39, 43, 45** and **46**); or (1) NaH, dry dimethylformamide, aminofurazan, 0 °C, 20 min; (2) 3-(trifluoromethyl)benzoyl chloride, dry dimethylformamide, 60 °C, 24 h (compounds **40, 44, 48** and **50**; or (1) 3-chloro-4-methoxybenzoic acid, *N*-hydroxy succinimide, DCC, dry tetrahydrofuran, rt, 24 h; (2) dry dimethylformamide, rt, 15 min; (3) NaH, dry dimethylformamide, aminofurazan, 0 °C, 20 min; (4) *N*-(aroyloxy)succinimide, dry dimethylformamide, 60 °C, 24 h (compounds **53** and **54**).

Prior to amide synthesis, the amino group of 3-aminofurazans **6, 7, 8, 21, 25** and **26** had to be deprotonated initially using NaH in dry DMF. Subsequently, the obtained anion was treated with the respective benzoyl chloride, giving amides **37–52**. Alternatively, reaction with *N*-(3-chloro-4-methoxybenzoyl)succinimide afforded 4-aminofurazans **53** and **54** (Figure 4) [15,21]. Amide bond formation was obvious from the appearance of signals for the amide proton and the new carbonyl carbon in NMR spectra, respectively.

**Figure 5:**
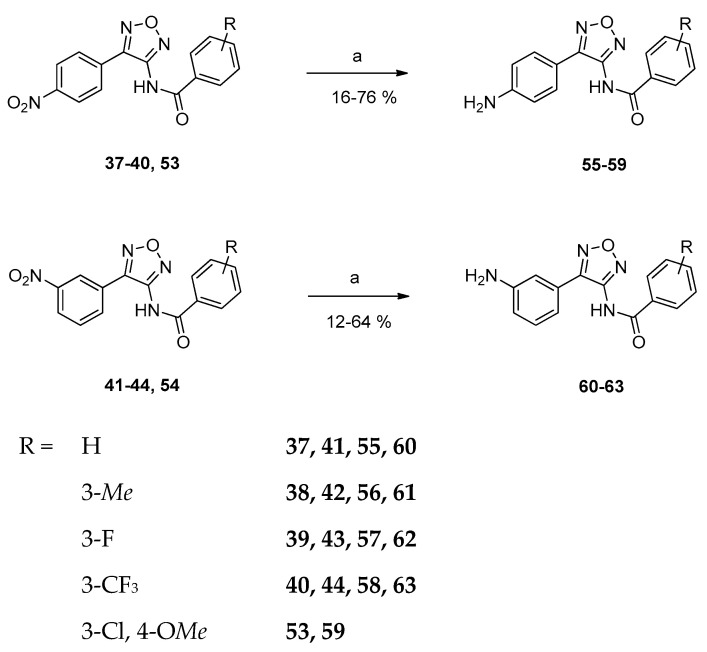
Preparation of compounds **55–63**. Reagents and conditions: (a) tin powder, 6*N* HCl, ethanol, 70 °C, 1 h.

To evaluate the influence of the electron-withdrawing nitro group in compounds **37–44** and **53,** they were treated with tin powder in a hydrochloric water ethanol mixture, giving their amino analogs **55–63** (Figure 5) [22].

### 2.2. Antiplasmodial Activity and Cytotoxicity

All newly synthesized 1,2,5-oxadiazoles were tested for their in vitro antiplasmodial activity against the chloroquine-sensitive strain NF54 of *Plasmodium falciparum*. Cytotoxicity was determined using L-6 cells (rat skeletal myofibroblasts) and podophyllotoxin as control. The IC_50_ values obtained are reported in Table 1. In order to evaluate the influence of various substituents of the phenyl moiety in ring position 4 of the 1,2,5-oxadiazole, the substituent pattern of the benzamide moieties of the five most promising compounds **1**–**5** was left unchanged (Figure 1). Consequently, these compounds served as comparison for all newly synthesized furazans. In general, 3-(trifluoromethyl)benzamides **4**, **40**, **44**, **48**, **50**, **58** and **63** (L-6 cells IC_50_ = 0.936–32.85 µM) showed the highest cytotoxicity, whereas unsubstituted benzamides **2**, **37**, **41**, **55** and **60** (L-6 cells IC_50_ = 6.067–216.9 µM) were usually the least cytotoxic, closely followed by their 3-methyl and 3-fluoro analogs. Compared with the formerly prepared compounds **1–5** (L-6 cells IC_50_ = 9.975–159.3 µM), a couple of the new derivatives exhibited similar or improved cytotoxicity. The 4-(aminophenyl) compounds **55**–**63** generally possessed low cytotoxicity (L-6 cells IC_50_ = 21.25–216.9 µM). Similar cytotoxicity (L-6 cells IC_50_ = 23.78–135.7 µM) was only observed in the 3-(3-methylbenzamido) series for the halogen-substituted compounds **47** and **49,** as well as their 3,4-disubstituted analogs **51** and **52**. The nitrophenyl compounds **37**–**44, 53** and **54** and the remaining new derivatives were much more cytotoxic (L-6 cells IC_50_ = 0.936–12.15 µM). The highest antiplasmodial activity was observed for 3-methylbenzamides **1** and **49** (*Pf*NF54 IC_50_ = 0.011–8.107 µM), their 3-trifluoromethyl analogs **63** and **48** (*Pf*NF54 IC_50_ = 8.444–17.69 µM) and the 3-chloro-4-methoxy derivatives **53**, **54** and **59** (*Pf*NF54 IC_50_ = 0.323–5.924 µM). Lowest antiplasmodial activity possessed 3-fluorobenzamides **39**, **43**, **46**, **47** and **62** (*Pf*NF54 IC_50_ = 5.331–26.69 µM) and unsubstituted benzamides **2** and **60** (*Pf*NF54 IC_50_ = 0.076–53.70 µM). The antiplasmodial activity of the new compounds (*Pf*NF54 IC_50_ = 0.034–53.70 µM) was generally lower in comparison with their formerly prepared analogs **1–5** (*Pf*NF54 IC_50_ = 0.011–0.076 µM). The nitro-substituted compounds **37**–**44**, **53** and **54** (*Pf*NF54 IC_50_ = 0.323–26.69 µM) were more active compared with their corresponding amino analogs **55–63** (*Pf*NF54 IC_50_ = 3.136–53.70 µM). The (4-nitrophenyl) **37**–**40** and **53** and the (4-aminophenyl) compounds **55–59** were more active than their respective 3-substituted analogs **41–44** and **60–63**. Two of these (4-nitrophenyl) derivatives **38** and **53** even showed activity in sub-micromolar concentrations, but their selectivity (S.I. = 3.504–10.37) was still low, similar to all selectivities in the nitrophenyl and aminophenyl series (S.I. ≤ 31.54). Two compounds of the 3-(3-methylbenzamido) series, the 3-ethoxy-4-methoxyphenyl substituted **51** (*Pf*NF54 IC_50_ = 0.034 µM) and its 4-ethoxy-3-methoxy analogue **52** (*Pf*NF54 IC_50_ = 0.275 µM), exhibited the highest antiplasmodial activity of the new derivatives. Due to its low cytotoxicity and very good selectivity, **51** (L-6 cells IC_50_ = 51.87 µM; S.I. = 1526) was the most promising of the new compounds. This strongly affirms the eminent positive impact of 4-(3,4-dialkoxyphenyl) substitution on activity and cytotoxicity of 1,2,5-oxadiazoles. The contribution of the 3-(3-methylbenzamido) substitution has a certain impact, but it is not a prerequisite for high activity and good selectivity.

### 2.3. Physicochemical Properties

In addition to in vitro antiplasmodial activity and cytotoxicity, physicochemical parameters essential for efficient lead discovery were determined. LogP values were calculated by using the ChemAxon software JChem for Excel 14.9.1500.912 (2014). Furthermore, ligand efficiency of compounds was calculated. CYP3A4-inhibtion and aqueous solubility were determined experimentally. Physicochemical parameters of compounds are summarized in Table 2. Lipophilicity of compounds is a fundamental property in drug development and optimization of lead compounds. The logP values of the compounds ranged between 2.45 and 4.47. The scarcely active 4-(4-aminophenyl) **56,** as well as the 4-(3-aminophenyl)furazans **60–63,** had good logP values (logP = 2.45–3.33). The most active compounds—**51**, **52** and **53**—exhibited only slightly higher logP values (logP = 3.67–3.84) that were, however, significantly lower than the ones of the lead compound **1** (logP = 4.19). Acceptable values of ligand efficiency for orally applied drug candidates are above 0.3 kcal per mole per heavy atom [23]. Out of all the newly synthesized compounds, the N-[4-(3-ethoxy-4-methoxyphenyl)-1,2,5-oxadiazol-3-yl]-3-methylbenzamide **51** was not only the most active derivative but also the one with the highest ligand efficiency (LE = 0.394), similar to that of the lead compound **1** (LE = 0.404).

The phase I liver enzyme CYP3A4 plays a crucial role in the metabolism of a multitude of orally applied drugs. Determination of CYP3A4-inhibition early in drug development helps to prioritize possible new drug candidates. Enzyme inhibition was determined for the newly synthesized compounds possessing the highest antiplasmodial activity. CYP3A4 inhibition of compounds could easily result in increased bioavailability of simultaneously applied drugs. The lead structure **1** exhibited moderate enzyme inhibition (45%) that was surpassed by most tested compounds. Only compounds **37** (46%) and **39** (43%) showed similar enzyme interactions.

Solubility of compounds in general, but especially aqueous solubility, is of significant importance for orally applied drugs and drug candidates. Solubility studies are often based on the use of DMSO stocks due to the fact that most in vitro and even in vivo studies involve initial dissolution of compounds in DMSO. The addition of an aqueous buffer to the DMSO stocks of compounds results in turbidity as soon as the aqueous solubility limit is reached. Precipitation was measured using a nephelometer [24,25]. The 4-(3-nitrophenyl)furazan derivatives **41** and **43** (0.84–0.98) were among the most soluble compounds, as well as the 4-(4-fluorophenyl) analog **45** (0.96). As was to be expected, the highest relative solubility (0.96–1.00) was shown by compounds **55** and **56** and **60–63**, all of which possessed an aminophenyl moiety.

## 3. Materials and Methods

### 3.1. Instrumentation and Chemicals

Melting points were obtained using the Electrothermal IA 9200 melting point apparatus (Fisher Scientific, Birmingham, UK); IR spectra (KBr discs) using the Bruker Alpha Platinum ATR FTIR spectrometer (Bruker, Etlingen, Germany). The frequencies were reported in cm^−1^. HRMS spectra were determined with a Q Exactive Hybrid Quadrupole-Orbitrap mass spectrometer (Thermo Fisher Scientific, Waltham, MA, USA) run by Thermo Q Exactive 2.9 (Thermo Fisher Scientific, Waltham, MA, USA) and Thermo Xcalibur^TM^ Software Version 4.4 (Thermo Fisher Scientific, Waltham, MA, USA) or with a Micromass Tofspec 3E spectrometer (MALDI) and GCT-Premier, Waters (EI, 70 eV). The structures of all newly prepared compounds were determined by one- and two-dimensional NMR-spectroscopy. NMR-spectra were obtained using a Varian UnityInova 400 MHz or a Bruker Avance Neo 400 MHz spectrometer, using 5 mm tubes and TMS as the internal standard. Shifts in ^1^H NMR (400 MHz) and ^13^C-NMR (100 MHz) spectra were reported in ppm; ^1^H- and ^13^C-signals were assigned using ^1^H,^1^H- and ^1^H,^13^C-correlation spectra and were numbered as shown in Figure 4. Signal multiplicities were abbreviated as follows: br—broad; d—doublet; dd—doublet of doublets; ddd—doublet of doublet of doublets; dt—doublet of triplets; m—multiplet; s—singlet; t—triplet; td—triplet of doublets; q—quartet. Assignments marked with an asterisk were interchangeable.

Materials consisted of thin layer chromatography (TLC): TLC plates silica gel 60 F_254_ (Merck); column chromatography (CC): silica gel 60 (Merck 70–230 mesh, pore diameter 60 Å) and flash silica gel (Merck, 230–400 mesh, pore diameter 60 Å); CYP3A4-inhibition assay: P450-Glo CYP3A4 Assay with Luciferin-IPA, NADPH Regeneration System and beetle luciferin, potassium salt (Promega Corporation, Madison, WI, USA), Corning Supersomes Human CYP3A4 + Oxidoreductase + b5 and Corning Supersomes Human P450 Oxidoreductase + b5 Negative Control (Corning, Glendale, AZ, USA), Ketoconazole Pharmaceutical Secondary Standard (Sigma Aldrich, Schnelldorf, Germany), 96-well white plate (Greiner Bio-One, Kremsmünster, Austria); SpectraMax M3 plate reader (Molecular Devices, San Jose, CA, USA). ^1^H- and ^13^C-NMR spectra of new compounds are available in the Supplementary Materials Section (Figures S1–S37).

### 3.2. Syntheses

#### 3.2.1. Preparation of Phenylmethanols

##### General Procedure for the Synthesis of Compounds **27** and **28**

The corresponding aldehyde was dissolved in dry methanol. NaBH_4_ was added in portions and the reaction mixture was stirred for 1 h at room temperature. After completion of the reaction, the solvent was evaporated in vacuo. Water was added and the aqueous phase was extracted three times with dichloromethane. The organic phases were combined and washed with water, dried over anhydrous sodium sulfate and filtered. The solvent was evaporated in vacuo.

(3-Ethoxy-4-methoxyphenyl)methanol (**27**): The reaction of 3-ethoxy-4-methoxybenzaldehyde (1.80 g (10.00 mmol)) with NaBH_4_ (0.38 g (10.00 mmol)) in dry methanol (15 mL) yielded compound **27** as a colorless oil (1.80 g (99%)). IR = 3266, 2934, 1590, 1517, 1458, 1426, 1373, 1260, 1242, 1161, 1137, 1027, 1004, 857, 818; ^1^H NMR (CDCl_3_, 400 MHz) δ = 1.47 (t, *J* = 7.0 Hz, 3H, CH_3_), 1.66 (s, 1H, OH), 3.87 (s, 3H, OCH_3_), 4.11 (q, *J* = 7.0 Hz, 2H, OCH_2_), 4.61 (s, 2H, ArCH_2_), 6.84 (d, *J* = 8.2 Hz, 1H, 5-H), 6.89 (dd, *J* = 8.2, 1.6 Hz, 1H, 6-H), 6.92 (d, *J* = 1.6 Hz, 1H, 2-H); ^13^C NMR (CDCl_3_, 100 MHz) δ = 14.79 (CH_3_), 55.98 (OCH_3_), 64.22 (OCH_2_), 65.33 (ArCH_2_), 111.29 (C-5), 111.83 (C-2), 119.37 (C-6), 133.48 (C-1), 148.41 (C-3), 148.83 (C-4); HRMS (MALDI) calcd. for: C_10_H_14_O_3_: 182.0943; found: 182.0935.

(4-Ethoxy-3-methoxyphenyl)methanol (**28**): The reaction of 4-ethoxy-3-methoxybenzaldehyde (1.80 g (10.00 mmol)) with NaBH_4_ (0.38 g (10.00 mmol)) in dry methanol (15 mL) gave compound **28** as a colorless oil (1.81 g (100%)). NMR data were in accordance with the literature data [26].

#### 3.2.2. Preparation of Benzyl Chlorides

##### General Procedure for the Preparation of Compounds **29** and **30**

The corresponding alcohol was dissolved in dry dichloromethane and the reaction mixture was cooled in an ice bath to 0 °C. Thionyl chloride was added slowly via a dropping funnel. The reaction mixture was stirred at room temperature for 24 h. After that, the mixture was cooled in an ice bath to 0 °C and basified to a pH of 10–11 using 2*N* NaOH. The aqueous and organic layers were separated, and the aqueous phase was extracted twice with dichloromethane. The organic phases were combined, dried over anhydrous sodium sulfate and filtered, and the solvent was evaporated in vacuo yielding the alkyl chlorides.

4-(Chloromethyl)-2-ethoxy-1-methoxybenzene (**29**): The reaction of compound **27** (2.15 g (11.80 mmol)) with thionyl chloride (4.07 g (34.22 mmol)) in dry dichloromethane (50 mL) yielded compound **29** as a brown oil (2.15 g (91%)). IR = 2977, 1606, 1508, 1442, 1392, 1340, 1255, 1199, 1137, 1089, 1017, 935, 872; ^1^H NMR (CDCl_3_, 400 MHz) δ = 1.47 (t, *J* = 7.0 Hz, 3H, CH_3_), 3.86 (s, 3H, OCH_3_), 4.11 (q, *J* = 7.0 Hz, 2H, OCH_2_), 4.55 (s, 2H, ArCH_2_), 6.82 (d, *J* = 8.7 Hz, 1H, 5-H), 6.90–6.92 (m, 2H, 2-H, 6-H); ^13^C NMR (CDCl_3_, 100 MHz) δ = 14.67 (CH_3_), 46.64 (ArCH_2_), 55.87 (OCH_3_), 64.25 (OCH_2_), 111.15 (C-5), 113.01 (C-2), 121.01 (C-6), 129.85 (C-1), 148.34 (C-3), 149.39 (C-4); HRMS (EI+) calcd. for C_10_H_13_ClO_2_: 200.0604; found: 200.0602.

4-(Chloromethyl)-1-ethoxy-2-methoxybenzene (**30**): Reaction of **28** (2.67 g (14.64 mmol)) with thionyl chloride (5.05 g (42.46 mmol)) in dry dichloromethane (50 mL) gave compound **30** as a brown oil (2.77 g (94%)). IR = 2980, 1610, 1517, 1468, 1423, 1342, 1263, 1238, 1166, 1137, 1036, 865, 800; ^1^H NMR (CDCl_3_, 400 MHz) δ = 1.32 (t, *J* = 6.9 Hz, 3H, CH_3_), 3.76 (s, 3H, OCH_3_), 4.00 (q, *J* = 6.9 Hz, 2H, OCH_2_), 4.71 (s, 2H, ArCH_2_), 6.91 (d, *J* = 8.3 Hz, 1H, 5-H), 6.95 (dd, *J* = 8.3, 1.7 Hz, 1H, 1-H, 6-H), 7.03 (d, *J* = 1.7 Hz, 1H, 2-H); ^13^C NMR (CDCl_3_, 100 MHz) δ = 14.69 (CH_3_), 46.73 (ArCH_2_), 55.41 (OCH_3_), 63.67 (OCH_2_), 112.56 (C-5), 112.61 (C-2), 121.45 (C-6), 129.84 (C-1), 148.08 (C-4), 148.76 (C-3); HRMS (EI+) calcd. for C_10_H_13_ClO_2_: 200.0604; found: 200.0600.

#### 3.2.3. Preparation of Nitriles

(4-Nitrophenyl)acetonitrile (**22**): H_2_SO_4_ 98% (27.50 mL) and HNO_3_ 67% (27.50 mL) were added to a three-neck round-bottom flask and cooled in an ice bath to 10 °C. Phenylacetonitrile (10.00 g (85.40 mmol)) was added slowly via a dropping funnel. The reaction mixture was stirred for 1 h at room temperature. After completion, the mixture was poured on ice. The precipitate was filtered with suction, washed with water and recrystallized from ethanol, giving compound **22** as a white amorphous solid (7.33 g (54%)). NMR data were in accordance with the literature data [27].

##### General Procedure for the Preparation of Compounds **31** and **32**

The corresponding alkyl chloride was dissolved in dry dimethylformamide. Potassium cyanide was added and the reaction mixture was stirred for 2 h at 100 °C. Another portion of potassium cyanide was added and the reaction mixture was stirred at 100 °C for a further 2 h. After completion, the mixture was cooled to room temperature and the solvent was evaporated in vacuo. Water and dichloromethane were added and the mixture was stirred intensely for 5 min. The phases were separated, and the aqueous phase was extracted twice with dichloromethane. The organic phases were combined, washed four times with brine, dried over anhydrous sodium sulfate and filtered, and the solvent was evaporated in vacuo. The residue was co-distilled twice with toluene, yielding the nitriles.

(3-Ethoxy-4-methoxyphenyl)acetonitrile (**31**): Compound **29** (1.91 g (9.50 mmol)) was dissolved in dry dimethylformamide (20 mL) and treated with potassium cyanide (1.86 g (28.50 mmol)), yielding the crude product. It was purified by column chromatography (silica gel, CH_2_Cl_2_/*Me*OH 99:1), giving compound **31** as a pale brown solid (1.42 g (78%)). m.P. 192 °C. IR = 2979, 2249, 1592, 1512, 1428, 1257, 1234, 1140, 1026, 983, 804; ^1^H NMR (CDCl_3_, 400 MHz) δ = 1.47 (t, *J* = 7.0 Hz, 3H, CH_3_), 3.68 (s, 2H, ArCH_2_), 3.87 (s, 3H, OCH_3_), 4.10 (q, *J* = 7.0 Hz, 2H, OCH_2_), 6.81 (s, 1H, 2-H), 6.84 (s, 2H, 5-H, 6-H); ^13^C NMR (CDCl_3_, 100 MHz) δ = 14.64 (CH_3_), 23.09 (ArCH_2_), 55.92 (OCH_3_), 64.37 (OCH_2_), 111.65 (C-5), 112.23 (C-2), 118.12 (CN), 120.10 (C-6), 122.00 (C-1), 148.64 (C-3), 148.99 (C-4); HRMS (EI+) calcd. for C_11_H_13_NO_2_: 191.0946; found: 191.0940.

(4-Ethoxy-3-methoxyphenyl)acetonitrile (**32**): The reaction of compound **30** (0.41 g (2.02 mmol)) with potassium cyanide (0.26 g (4.04 mmol)) in dry dimethylformamide (19 mL) gave the crude product. It was purified by column chromatography (silica gel, CH_2_Cl_2_/*Me*OH 39:1), yielding compound **32** as a white amorphous solid (0.13 g (34%)). IR = 2978, 2929, 2250, 1593, 1515, 1473, 1420, 1396, 1340, 1297, 1234, 1191, 1142, 1043, 1026, 918, 892, 849, 805; ^1^H NMR (CDCl_3_, 400 MHz) δ = 1.46 (t, *J* = 7.0 Hz, 3H, CH_3_), 3.69 (s, 2H, ArCH_2_), 3.88 (s, 3H, OCH_3_), 4.09 (q, *J* = 7.0 Hz, 2H, OCH_2_), 6.81 (s, 1H, 2-H), 6.84 (s, 2H, 5-H, 6-H); ^13^C NMR (CDCl_3_, 100 MHz) δ = 14.66 (CH_3_), 23.12 (ArCH_2_), 55.92 (OCH_3_), 64.33 (OCH_2_), 111.12 (C-2), 112.79 (C-5), 118.12 (CN), 120.13 (C-6), 121.99 (C-1), 148.02 (C-4), 149.59 (C-3); HRMS (EI+) calcd. for C_11_H_13_NO_2_: 191.0946; found: 191.0940.

#### 3.2.4. Preparation of Oximes

##### General Procedure for the Preparation of Compounds **9**, **10** and **11**

Hydroxylamine hydrochloride and sodium bicarbonate were dissolved in water. The mixture was added to a solution of the corresponding aldehyde in methanol. The reaction mixture was stirred at 100 °C for 2 h. After completion, methanol was evaporated in vacuo. Then, water was added to the residue. The aqueous phase was extracted twice with ethyl acetate. The organic phases were combined, dried over anhydrous sodium sulfate and filtered, and the solvent was removed in vacuo, yielding the desired oximes.

(4-Chlorophenyl)-*N*-hydroxymethanimin (**9**): The reaction of 4-chlorobenzaldehyde (0.84 g (6.00 mmol)) with hydroxylamine hydrochloride (0.50 g (7.20 mmol)) and NaHCO_3_ (0.61 g (7.20 mmol)) in water (9 mL) and methanol (18 mL) gave compound **9** as a white amorphous solid (0.89 g (95%)). NMR data were in accordance with the literature data [15].

(4-Fluorophenyl)-*N*-hydroxymethanimin (**10**): The reaction of 4-fluorobenzaldehyde (0.75 g (6.00 mmol)) with hydroxylamine hydrochloride (0.50 g (7.20 mmol)) and NaHCO_3_ (0.61 g (7.20 mmol)) in water (9 mL) and methanol (18 mL) gave compound **10** as a white amorphous solid (0.83 g (100%)). NMR data were in accordance with the literature data [28].

*N*-Hydroxy-(3-nitrophenyl)methanimin (**11**): The reaction of 3-nitrobenzaldehyde (6.05 g (40.00 mmol)) with hydroxylamine hydrochloride (3.34 g (48.00 mmol)) and NaHCO_3_ (4.03 g (48.00 mmol)) in water (60 mL) and methanol (120 mL) gave compound **11** as a white amorphous solid (6.49 g (98%)). NMR data were in accordance with the literature data [29].

#### 3.2.5. Preparation of Imidoyl Chlorides

##### General Procedure for the Preparation of Compounds **12** and **13**

The corresponding oxime was dissolved in dry dimethylformamide. *N*-Chlorosuccinimide was added and the reaction mixture was cooled in an ice bath to 0 °C. Another portion of *N*-chlorosuccinimide was added and the reaction mixture was stirred at room temperature overnight. After that, water was added and the aqueous phase was extracted three times with ethyl acetate. The organic phases were combined, washed with water and brine, dried over anhydrous sodium sulfate and filtered, and the solvent was evaporated in vacuo.

4-Chloro-*N*-hydroxybenzenecarboximidoyl chloride (**12**): The reaction of **9** (0.47 g (3.00 mmol)) with *N*-chlorosuccinimide (0.40 g (3.00 mol)) in dry dimethylformamide (5 mL) yielded compound **12** as a white amorphous solid (0.54 g (94%)). NMR data were in accordance with the literature data [15].

4-Fluoro-*N*-hydroxybenzenecarboximidoyl chloride (**13**): The reaction of **10** (0.83 g (6.00 mmol)) with *N*-chlorosuccinimide (0.80 g (6.00 mmol)) in dry dimethylformamide (8 mL) yielded compound **13** as a yellow amorphous solid (0.94 g (90%)). NMR data were in accordance with the literature data [28].

#### 3.2.6. Preparation of Hydroxyimino Nitriles

(Hydroxyimino)(3-nitrophenyl)acetonitrile (**17**): The oxime 11 (6.49 g (39.10 mmol)) was dissolved in dry dimethylformamide (28 mL). *N*-chlorosuccinimide (0.58 g (4.40 mmol)) was added and the reaction mixture was heated to 45 °C for 10 min. Afterwards, the mixture was cooled to room temperature, another portion of *N*-chlorosuccinimide (4.64 g (34.70 mmol)) was added and the reaction mixture was stirred at room temperature for 1 h. The intermediate **14** was not isolated. The mixture was added dropwise to an ice-cooled solution of potassium cyanide (5.60 g (86.02 mmol)) in ethyl acetate (100 mL). After that, the reaction mixture was stirred at 5 °C for 30 min. After completion, the phases were separated. The aqueous phase was extracted three times with ethyl acetate. The organic phases were combined, washed with brine, dried over anhydrous sodium sulfate and filtered. The solvent was evaporated in vacuo, yielding the crude product. It was purified by column chromatography (silica gel, CH_2_Cl_2_/EtAc 29:1) giving compound **17** as a white amorphous solid (5.38 g (72%)). NMR data were in accordance with the literature data [30].

##### General Procedure for the Preparation of Compounds **15** and **16**

The corresponding imidoyl chloride was dissolved in diethyl ether. A solution of potassium cyanide in water was added. The reaction mixture was stirred at room temperature overnight. After that, the phases were separated. The aqueous phase was washed three times with ethyl acetate. The organic phases were combined, washed with water, dried over anhydrous sodium sulfate and filtered. The solvent was evaporated in vacuo.

(4-Chlorophenyl)(hydroxyimino)acetonitrile (**15**): The reaction of **12** (0.48 g (2.50 mmol)) with potassium cyanide (0.33 g (5.00 mmol)) in diethyl ether (11 mL) and water (5 mL) gave compound **15** as a yellow oil (0.42 g (92%)). NMR data were in accordance with the literature data [15].

(4-Fluorophenyl)(hydroxyimino)acetonitrile (**16**): The reaction of **13** (0.86 g (4.96 mmol)) with potassium cyanide (0.65 g (9.92 mmol)) in diethyl ether (20 mL) and water (10 mL) gave compound **16** as a yellow oil (0.73 g (90%)). NMR data were in accordance with the literature data [30].

##### General Procedure for the Preparation of Compounds **23**, **33** and **34**

Sodium was dissolved in dry ethanol. The formed sodium ethoxide was cooled to 0 °C. A solution of the corresponding nitrile in dry ethanol was added via a dropping funnel. Isoamyl nitrite was added with a syringe via a septum. The reaction mixture was stirred at room temperature overnight. After that, ethyl acetate was added. The organic phase was washed twice with 2*N* HCl, aqueous NaHCO_3_ and brine, respectively, dried over anhydrous sodium sulfate and filtered. The solvent was evaporated in vacuo, yielding the hydroxyimino derivatives.

(Hydroxyimino)(4-nitrophenyl)acetonitrile (**23**): Sodium (2.06 g (89.42 mmol)) was dissolved in dry ethanol (65 mL). To that solution, **22** (7.25 g (44.71 mmol)) was dissolved in dry ethanol (50 mL), followed by isoamyl nitrite (7.86 g (67.07 mmol)) being added. The crude product was recrystallized from toluene, yielding compound **23** as a light yellow amorphous solid (5.14 g (60%)). NMR data were in accordance with the literature data [31].

(3-Ethoxy-4-methoxyphenyl)(hydroxyimino)acetonitrile (**33**): Sodium (0.34 g (14.80 mmol)) was dissolved in dry ethanol (12 mL). To that solution, **31** (1.42 g (7.41 mmol)) was dissolved in dry ethanol (7 mL), followed by isoamyl nitrite (1.30 g (11.10 mmol)) being added. The crude product was recrystallized from toluene, yielding compound **33** as a pale brown solid (0.42 g (26%)). m.P.: 192 °C. IR = 3395, 1603, 1516, 1436, 1341, 1274, 1215, 1180, 1142, 1079, 1009, 852; ^1^H NMR (DMSO-d_6_, 400 MHz) δ = 1.34 (t, *J* = 6.8 Hz, 3H, CH_3_), 3.83 (s, 3H, OCH_3_), 4.04 (q, *J* = 6.8 Hz, 2H, OCH_2_), 7.10 (d, *J* = 8.8 Hz, 1H, 5-H), 7.21–7.23 (m, 2H, 2-H, 6-H), 13.46 (br, 1H, NOH); ^13^C NMR (DMSO-d_6_, 100 MHz) δ = 14.62 (CH_3_), 55.66 (OCH_3_), 63.81 (OCH_2_), 108.13 (C-2), 110.24 (CN), 111.86 (C-5), 119.55 (C-6), 122.03 (C-1), 130.89 (C=NOH), 148.40 (C-3), 151.33 (C-4); HRMS (EI+) calcd. for C_11_H_12_N_2_O_3_: 220.0848; found: 220.0837.

(4-Ethoxy-3-methoxyphenyl)(hydroxyimino)acetonitrile (**34**): Sodium (0.47 g (20.28 mmol)) was dissolved in dry ethanol (15 mL). To that solution, **32** (1.94 g (10.14 mmol)) was dissolved in dry ethanol (10 mL), followed by isoamyl nitrite (1.78 g (15.21 mmol)) being added. The crude product was co-distilled with toluene four times, yielding compound **34** as a pale brown solid (1.51 g (68%)). m.P.: 161 °C. IR = 3364, 2987, 2938, 1602, 1516, 1461, 1419, 1400, 1343, 1292, 1277, 1218, 1148, 1027, 993, 854, 800; ^1^H NMR (DMSO-d_6_, 400 MHz) δ = 1.34 (t, *J* = 6.8 Hz, 3H, CH_3_), 3.80 (s, 3H, OCH_3_), 4.07 (q, *J* = 6.8 Hz, 2H, OCH_2_), 7.07 (d, *J* = 8.4 Hz, 1H, 5-H), 7.20 (dd, *J* = 8.4, 2.0 Hz, 1H, 6-H), 7.24 (d, *J* = 2.0 Hz, 1H, 2-H), 13.43 (s, 1H, NOH); ^13^C NMR (DMSO-d_6_, 100 MHz) δ = 14.55 (CH_3_), 55.40 (OCH_3_), 63.88 (OCH_2_), 107.23 (C-2), 110.20 (CN), 112.51 (C-5), 119.58 (C-6), 121.91 (C-1), 130.85 (C=NOH), 149.23 (C-3), 150.44 (C-4); HRMS (EI+) calcd. for C_11_H_12_N_2_O_3_: 220.0848; found: 220.0833.

#### 3.2.7. Preparation of Amide Oximes

4-(4-Fluorophenyl)-1,2,5-oxadiazol-3-amine (**7**): Nitrile **16** (0.77 g (4.67 mmol)) was dissolved in methanol (13 mL). To that solution, a mixture of hydroxylamine hydrochloride (0.49 g (7.10 mmol)) and sodium bicarbonate (0.60 g (7.10 mmol)) in water (6 mL) was added. The reaction mixture was refluxed at 100 °C overnight. After that, the solvent was evaporated in vacuo. The crude residue of 19 was dissolved in 2*N* NaOH (27.5 mL (55.00 mmol)) and refluxed at 125 °C overnight. After that, the suspension was cooled to room temperature. The precipitate was filtered and washed with water, yielding compound **7** as a white amorphous solid (0.32 g (66%)). NMR data were in accordance with the literature data [32].

4-(3-Nitrophenyl)-1,2,5-oxadiazol-3-amine (**8**): Nitrile **17** (5.38 g (28.20 mmol)) was dissolved in methanol (90 mL). To that solution, a mixture of hydroxylamine hydrochloride (2.94 g (42.30 mmol)) and sodium bicarbonate (3.55 g (42.30 mmol)) in water (40 mL) was added. The reaction mixture was refluxed at 100 °C overnight. After that, the solvent was evaporated in vacuo. To the crude residue of 20, anhydrous sodium acetate (6.62 g (80.75 mmol)) and dry ethanol (280 mL) were added. The reaction mixture was refluxed at 100 °C for 120 h. After completion, the solvent was evaporated in vacuo. The residue was dissolved in ethyl acetate and the organic phase was washed with water. The aqueous phase was extracted twice with ethyl acetate. The organic phases were combined, dried over anhydrous sodium sulfate and filtered. The solvent was evaporated in vacuo, giving the crude product. It was purified by column chromatography (flash silica gel, CH_2_Cl_2_/EtAc 29:1), yielding compound **8** as a yellow amorphous solid (0.71 g (21%)). NMR data were in accordance with the literature data [33].

4-(4-Nitrophenyl)-1,2,5-oxadiazol-3-amine (**21**): The amide oxime **24** (5.63 g (25.11 mmol)) and anhydrous sodium acetate (10.30 g (125.60 mmol)) were dissolved in dry ethanol (440 mL) and refluxed for 192 h. After that, the solvent was evaporated in vacuo. The residue was dissolved in ethyl acetate and the organic phase was washed with water. The aqueous phase was extracted twice with ethyl acetate. The organic phases were combined, dried over anhydrous sodium sulfate and filtered. The solvent was evaporated in vacuo, giving the crude product. It was purified by column chromatography (flash silica gel, CH_2_Cl_2_/EtAc gradient from 9:1 to 1:1), yielding compound **21** as a yellow amorphous solid (1.92 g (37%)). NMR data were in accordance with the literature data [17].

##### General Procedure for the Preparation of Compounds **18**, **24**, **35** and **36**

The corresponding nitrile was dissolved in methanol. To that solution, a mixture of hydroxylamine hydrochloride and sodium bicarbonate in water was added. The reaction mixture was refluxed at 100 °C overnight. After that, methanol was evaporated in vacuo. Water was added to the residue and the aqueous phase was extracted three times with ethyl acetate. The organic phases were combined, dried over anhydrous sodium sulfate and filtered. The solvent was evaporated in vacuo, yielding the crude products, which were purified by column chromatography or recrystallization.

2-(4-Chlorophenyl)-*N′*-hydroxy-2-(hydroxyimino)ethanimidamide (**18**): The reaction of compound **15** (0.42 g (2.34 mmol)), hydroxylamine hydrochloride (0.24 g (3.50 mmol)) and NaHCO_3_ (0.29 g (3.50 mmol)) in methanol (7 mL) and water (3 mL) yielded the crude product. It was recrystallized from dichloromethane, giving compound **18** as a white solid (0.29 g (58%)). m.P.: 166 °C. NMR data were in accordance with the literature data [15].

*N′*-Hydroxy-2-(hydroxyimino)-2-(4-nitrophenyl)ethanimidamide (**24**): The reaction of compound **23** (5.14 g (26.88 mmol)), hydroxylamine hydrochloride (2.80 g (40.31 mmol)) and NaHCO_3_ (3.39 g (40.32 mmol)) in methanol (90 mL) and water (38 mL) yielded compound **24** as a pale green amorphous solid (5.63 g (93%)). NMR data were in accordance with the literature data [17].

2-(3-Ethoxy-4-methoxyphenyl)-*N′*-hydroxy-2-(hydroxyimino)ethanimidamide (**35**): The reaction of compound **33** (0.42 g (1.89 mmol)), hydroxylamine hydrochloride (0.30 g (4.35 mmol)) and NaHCO_3_ (0.37 g (4.35 mmol)) in methanol (11 mL) and water (5 mL) yielded the crude product. It was recrystallized from dichloromethane, giving compound **35** as a beige solid (0.33 g (68%)). m.P.: 179 °C. IR = 3360, 2972, 1650, 1599, 1518, 1444, 1270, 1209, 1179, 1144, 1059, 1019, 982, 931, 870, 800; ^1^H NMR (DMSO-d_6_, 400 MHz) δ = 1.33 (t, *J* = 7.0 Hz, 3H, CH_3_), 3.77 (s, 3H, OCH_3_), 3.98 (q, *J* = 7.0 Hz, 2H, OCH_2_), 5.68 (s, 2H, NH_2_), 6.96 (d, *J* = 8.2 Hz, 1H, 5-H), 7.06 (dd, *J* = 8.2, 1.9 Hz, 1H, 6-H), 7.20 (d, *J* = 1.9 Hz, 1H, 2-H), 9.39 (s, 1H, NOH), 11.35 (s, 1H, NOH); ^13^C NMR (DMSO-d_6_, 100 MHz) δ = 14.74 (CH_3_), 55.49 (OCH_3_), 63.66 (OCH_2_), 109.90 (C-2), 111.27 (C-5), 120.17 (C-6), 127.24 (C-1), 146.63 (C(=NOH)NH_2_), 147.50 (C-4), 149.22 (C=NOH), 149.92 (C-3); HRMS (EI+) calcd. for C_11_H_15_N_3_O_4_: 253.1063; found: 253.1051.

2-(4-Ethoxy-3-methoxyphenyl)-*N′*-hydroxy-2-(hydroxyimino)ethanimidamide (**36**): The reaction of compound **34** (0.40 g (1.82 mmol)), hydroxylamine hydrochloride (0.29 g (4.19 mmol)) and NaHCO_3_ (0.35 g (4.19 mmol)) in methanol (8 mL) and water (4 mL) yielded the crude product. It was recrystallized from dichloromethane, giving compound **36** as a beige solid (0.33 g (70%)). m.P.: 173 °C. IR = 3375, 3238, 2976, 2926, 1668, 1601, 1515, 1470, 1422, 1390, 1342, 1269, 1216, 1147, 1022, 967, 848, 809; ^1^H NMR (DMSO-d_6_, 400 MHz) δ = 1.33 (t, *J* = 7.0 Hz, 3H, CH_3_), 3.74 (s, 3H, OCH_3_), 4.02 (q, *J* = 7.0 Hz, 2H, OCH_2_), 5.68 (s, 2H, NH_2_), 6.94 (d, *J* = 8.5 Hz, 1H, 5-H), 7.04 (dd, *J* = 8.5, 1.8 Hz, 1H, 6-H), 7.22 (d, *J* = 1.8 Hz, 1H, 2-H), 9.40 (s, 1H, NOH), 11.35 (s, 1H, NOH); ^13^C NMR (DMSO-d_6_, 100 MHz) δ = 14.67 (CH_3_), 55.31 (OCH_3_), 63.68 (OCH_2_), 108.73 (C-2), 112.07 (C-5), 120.23 (C-6), 127.19 (C-1), 146.62 (C(=NOH)NH_2_), 148.46 (C-4), 149.95 (C-3), 149.21 (C=NOH); HRMS (EI+) calcd. for C_11_H_15_N_3_O_4_: 253.1063; found: 253.1053.

#### 3.2.8. Preparation of Furazan-3-Amines

##### General Procedure for the Preparation of Compounds **6**, **25** and **26**

The corresponding amide oxime and 2*N* NaOH were refluxed at 125 °C overnight. After that, the suspension was cooled to room temperature. The precipitate was filtered and washed with water, yielding the desired 1,2,5-oxadiazoles.

4-(4-Chlorophenyl)-1,2,5-oxadiazol-3-amine (**6**): The reaction of **18** (0.26 g (1.23 mmol)) with 2*N* NaOH (12.3 mL (24.60 mmol)) yielded compound **6** as a white amorphous solid (0.18 g (76%)). NMR data were in accordance with the literature data [15].

4-(3-Ethoxy-4-methoxyphenyl)-1,2,5-oxadiazol-3-amine (**25**): The reaction of **35** (0.33 g (1.28 mmol)) with 2*N* NaOH (12.8 mL (25.60 mmol)) yielded compound **25** as a beige amorphous solid (0.25 g (84%)). IR = 3337, 2981, 1621, 1535, 1494, 1443, 1406, 1326, 1303, 1280, 1253, 1217, 1174, 1142, 1047, 1020, 936, 876, 846, 812; ^1^H NMR (DMSO-d_6_, 400 MHz) δ = 1.35 (t, *J* = 7.0 Hz, 3H, CH_3_), 3.83 (s, 3H, OCH_3_), 4.09 (q, *J* = 7.0 Hz, 2H, OCH_2_), 6.15 (s, 2H, NH_2_), 7.11 (d, *J* = 8.2 Hz, 1H, 5′-H), 7.25 (d, *J* = 1.2 Hz, 1H, 2′-H), 7.31 (dd, *J* = 8.2, 1.2 Hz, 1H, 6′-H); ^13^C NMR (DMSO-d_6_, 100 MHz) δ = 14.66 (CH_3_), 55.58 (OCH_3_), 63.73 (OCH_2_), 111.70 (C-2′), 112.03 (C-5′), 117.67 (C-1′), 120.54 (C-6′), 146.70 (C-4), 148.19 (C-3′), 150.56 (C-4′), 155.23 (C-3); HRMS (EI+) calcd. for C_11_H_13_N_3_O_3_: 235.0957; found: 235.0952.

4-(4-Ethoxy-3-methoxyphenyl)-1,2,5-oxadiazol-3-amine (**26**): The reaction of **36** (0.30 g (1.19 mmol) with 2*N* NaOH (11.90 mL (23.80 mmol)) yielded compound **26** as a beige amorphous solid (0.40 g (86%)). IR = 3355, 3236, 2979, 2939, 1650, 1596, 1540, 1499, 1475, 1411, 1306, 1279, 1249, 1220, 1175, 1148, 1030, 987, 861, 849, 812; ^1^H NMR (DMSO-d_6_, 400 MHz) δ = 1.36 (t, *J* = 7.0 Hz, 3H, CH_3_), 3.84 (s, 3H, OCH_3_), 4.08 (q, *J* = 7.0 Hz, 2H, OCH_2_), 6.15 (s, 2H, NH_2_), 7.09 (d, *J* = 8.3 Hz, 1H, 5′-H), 7.26 (d, *J* = 1.8 Hz, 1H, 2′-H), 7.30 (dd, *J* = 8.3, 1.8 Hz, 1H, 6′-H); ^13^C NMR (DMSO-d_6_, 100 MHz) δ = 14.63 (CH_3_), 55.44 (OCH_3_), 63.80 (OCH_2_), 110.88 (C-2′), 112.80 (C-5′), 117.59 (C-1′), 120.54 (C-6′), 146.69 (C-4), 149.04 (C-3′), 149.67 (C-4′), 155.21 (C-3); HRMS (EI+) calcd. for C_11_H_13_N_3_O_3_: 235.0957; found: 235.0950.

#### 3.2.9. Preparation of Benzamides

##### General Procedure for the Preparation of Compounds **37**–**52**

NaH (60% dispersion in mineral oil) was suspended in dry dimethylformamide and cooled in an ice bath to 0 °C. The corresponding 5-substituted 1,2,5-oxadiazol-3-amine was added and the reaction mixture was stirred for 20 min at 0 °C. After that, a solution of the respective acid chloride in dry dimethylformamide was added via a dropping funnel. The reaction mixture was heated to 60 °C for 24–48 h. After completion of the reaction, the mixture was cooled to 0 °C and water was added. The aqueous phase was extracted three times with dichloromethane. The organic phases were combined, washed with aqueous NaHCO_3_ and brine, dried over anhydrous sodium sulfate and filtered. The solvent was evaporated in vacuo, yielding the crude products, which were purified by column chromatography or recrystallized.

*N*-[4-(4-Nitrophenyl)-1,2,5-oxadiazol-3-yl]benzamide (**37**): The reaction of compound **21** (0.39 g (1.88 mmol)), benzoyl chloride (0.49 g (3.50 mmol)) and NaH (60% dispersion in mineral oil) (0.16 g (4.00 mmol)) in dry dimethylformamide (26 mL) gave the crude product. It was recrystallized from dichloromethane, yielding compound **37** as a light yellow solid (0.4 g (68%)). m.P.: 208 °C. IR = 3426, 1675, 1525, 1475, 1342, 1301, 1108, 998, 855, 718, 691; ^1^H NMR (DMSO-d_6_, 400 MHz) δ = 7.56 (t, *J* = 7.5 Hz, 2H, 3-H, 5-H), 7.67 (t, *J* = 7.4 Hz, 1H, 4-H), 7.97 (d, *J* = 7.4 Hz, 2H, 2-H, 6-H), 8.02 (d, *J* = 8.7 Hz, 2H, 2″-H, 6″-H), 8.35 (d, *J* = 8.7 Hz, 2H, 3″-H, 5″-H), 11.60 (s, 1H, NH); ^13^C NMR (DMSO-d_6_, 100 MHz) δ = 124.20 (C-3″, C-5″), 128.23 (C-2, C-6), 128.72 (C-3, C-5), 128.83 (C-2″, C-6″), 131.80 (C-1″), 131.96 (C-1), 132.99 (C-4), 148.64 (C-4″), 150.22 (C-4′*), 150.37 (C-3′*), 166.40 (C=O); HRMS (ESI-) calcd. for C_15_H_9_N_4_O_4_^−^ [M-H]^−^: 309.0618, found: 309.0632.

3-Methyl-*N*-[4-(4-nitrophenyl)-1,2,5-oxadiazol-3-yl]benzamide (**38**): The reaction of compound **21** (0.41 g (2.00 mmol)), 3-methylbenzoyl chloride (0.54 g (3.50 mol)) and NaH (60% dispersion in mineral oil) (0.16 g (4.00 mmol)) in dry dimethylformamide (26 mL) gave the crude product. It was purified by column chromatography (silica gel, CH_2_Cl_2_/EtAc 49:1), yielding compound **38** as a light yellow amorphous solid (0.53 g (82%)). IR = 3441, 3220, 1673, 1608, 1573, 1527, 1481, 1345, 1288, 998, 854, 749, 689; ^1^H NMR (DMSO-d_6_, 400 MHz) δ = 2.39 (s, 3H, CH_3_), 7.44 (t, *J* = 7.5 Hz, 1H, 5-H), 7.48 (t, *J* = 7.4 Hz, 1H, 4-H), 7.77 (d, *J* = 7.4 Hz, 1H, 6-H), 7.79 (s, 1H, 2-H), 8.01 (d, *J* = 8.1 Hz, 2H, 2″-H, 6″-H), 8.35 (d, *J* = 8.1 Hz, 2H, 3″-H, 5″-H), 11.56 (s, 1H, NH); ^13^C NMR (DMSO-d_6_, 100 MHz) δ = 20.88 (CH_3_), 124.20 (C-3″, C-5″), 125.39 (C-6), 128.61 (C-5), 128.71 (C-2), 128.81 (C-2″, C-6″), 131.85 (C-1″), 131.94 (C-1), 133.56 (C-4), 138.14 (C-3), 148.63 (C-4″), 150.24 (C-4′*), 150.44 (C-3′*), 166.48 (C=O); HRMS (ESI-) calcd. for C_16_H_11_N_4_O_4_^−^ [M-H]^−^: 323.0775, found: 323.0787.

3-Fluoro-*N*-[4-(4-nitrophenyl)-1,2,5-oxadiazol-3-yl]benzamide (**39**): The reaction of compound **21** (0.41 g (2.00 mmol)), 3-fluorobenzoyl chloride (0.56 g (3.50 mmol)) and NaH (60% dispersion in mineral oil) (0.16 g (4.00 mmol)) in dry dimethylformamide (26 mL) gave the crude product. It was purified by column chromatography (silica gel, cyclohexane/EtAc 2:1), yielding compound **39** as a light yellow amorphous solid (0.4 g (61%)). IR = 3442, 2360, 1676, 1525, 1477, 1346, 1281, 910, 855, 758, 669; ^1^H NMR (DMSO-d_6_, 400 MHz) δ = 7.54 (td, *J* = 8.8, 2.0 Hz, 1H, 4-H), 7.62 (td, *J* = 7.9, 5.8 Hz, 1H, 5-H), 7.76–7.82 (m, 2H, 2-H, 6-H), 8.04 (d, *J* = 8.5 Hz, 2H, 2″-H, 6″-H), 8.36 (d, *J* = 8.5 Hz, 2H, 3″-H, 5″-H), 11.72 (s, 1H, NH); ^13^C NMR (DMSO-d_6_, 100 MHz) δ = 115.10 (d, *J* = 22.9 Hz, C-2), 119.92 (d, *J* = 21.4 Hz, C-4), 124.20 (C-3″, C-5″), 124.48 (d, *J* = 3.0 Hz, C-6), 128.88 (C-2″, C-6″), 130.98 (d, *J* = 8.1 Hz, C-5), 131.69 (C-1″), 134.23 (d, *J* = 6.7 Hz, C-1), 148.65 (C-4″), 150.12 (C-4′*), 150.17 (C-3′*), 161.91 (d, *J* = 245 Hz, C-3), 161.12 (d, *J* = 2.8 Hz, C=O); HRMS (ESI-) calcd. for C_15_H_8_FN_4_O_4_^−^ [M-H]^−^: 327.0524, found: 327.0537.

*N*-[4-(4-Nitrophenyl)-1,2,5-oxadiazol-3-yl]-3-(trifluoromethyl)benzamide (**40**): The reaction of compound **21** (0.82 g (4.00 mmol)), 3-(trifluoromethyl)benzoyl chloride (1.460 g (7.00 mmol)) and NaH (60% dispersion in mineral oil) (0.32 g (8.00 mmol)) in dry dimethylformamide (52 mL) gave the crude product. It was recrystallized from dichloromethane, yielding compound **40** as a yellow solid (0.59 g (40%)). m.P.: 147 °C. IR = 3441, 3229, 1680, 1527, 1335, 1262, 1175, 1123, 1074, 998, 896, 861, 755; ^1^H NMR (DMSO-d_6_, 400 MHz) δ = 7.81 (t, *J* = 7.9 Hz, 1H, 5-H), 8.03–8.07 (m, 1H, 2″-H, 4-H, 6″-H), 8.24 (d, *J* = 7.9 Hz, 1H, 6-H), 8.34 (s, 1H, 2-H), 8.35 (d, *J* = 8.6 Hz, 2H, 3″-H, 5″-H), 11.91 (s, 1H, NH); ^13^C NMR (DMSO-d_6_, 100 MHz) δ = 123.82 (q, *J* = 273 Hz, CF_3_), 124.17 (C-3″, C-5″), 124.89 (q, *J* = 3.4 Hz, C-2), 128.94 (C-2″, C-6″), 129.38 (q, *J* = 32.3 Hz, C-3), 129.45 (q, *J* = 3.0 Hz, C-4), 130.08 (C-5), 131.79 (C-1″), 132.44 (C-6), 133.00 (C-1), 148.64 (C-4″), 150.07 (C-4′*), 150.30 (C-3′*), 165.03 (C=O); HRMS (ESI-) calcd. for: C_16_H_8_F_3_N_4_O_4_^−^ [M-H]^−^: 377.0492, found: 377.0506.

*N*-[4-(3-Nitrophenyl)-1,2,5-oxadiazol-3-yl]benzamide (**41**): The reaction of compound **8** (0.21 g (1.00 mmol)), benzoyl chloride (0.32 g (2.25 mmol)) and NaH (60% dispersion in mineral oil) (0.08 g (3.30 mmol)) in dry dimethylformamide (13 mL) gave the crude product. It was recrystallized from dichloromethane, yielding compound **41** as light yellow solid (0.14 g (46%)). m.P.: 179 °C. IR = 3427, 3222, 3091, 1670, 1595, 1596, 1530, 1508, 1483, 1378, 1351, 1306, 1270, 1104, 1008, 901, 821, 734, 714, 649; ^1^H NMR (DMSO-d_6_, 400 MHz) δ = 7.57 (t, *J* = 7.5 Hz, 2H, 3-H, 5-H), 7.68 (t, *J* = 7.4 Hz, 1H, 4-H), 7.84 (t, *J* = 8.1 Hz, 1H, 5″-H), 7.97 (d, *J* = 7.1 Hz, 2H, 2-H, 6-H), 8.21 (ddd, *J* = 7.8, 2.0, 1.0 Hz, 1H, 6″-H), 8.39 (ddd, *J* = 8.0, 2.0, 1.0 Hz, 1H, 4″-H), 8.55 (t, *J* = 2.0 Hz, 1H, 2″-H), 11.59 (s, 1H, NH); ^13^C NMR (DMSO-d_6_, 100 MHz) δ = 122.01 (C-2″), 125.37 (C-4″), 126.98 (C-1″), 128.11 (C-2, C-6), 128.70 (C-3, C-5), 130.90 (C-5″), 132.04 (C-1), 132.94 (C-4), 133.75 (C-6″), 147.93 (C-3″), 149.93 (C-4′), 150.26 (C-3′), 166.58 (C=O); HRMS (ESI-) calcd. for: C_15_H_9_N_4_O_4_^−^ [M-H]^−^: 309.0618, found: 309.0632.

3-Methyl-*N*-[4-(3-nitrophenyl)-1,2,5-oxadiazol-3-yl]benzamide (**42**): The reaction of compound **8** (0.41 g (2.00 mmol)), 3-methylbenzoyl chloride (0.54 g (3.50 mol)) and NaH (60% dispersion in mineral oil) (0.16 g (4.00 mmol)) in dry dimethylformamide (26 mL) gave the crude product. Recrystallization from dichloromethane yielded compound **42** as a light yellow solid (0.43 g (66%)). m.P.: 158 °C. IR = 3223, 1671, 1536, 1490, 1369, 1349, 1277, 1199, 1009, 892, 813, 754, 719, 678; ^1^H NMR (DMSO-d_6_, 400 MHz) δ = 2.39 (s, 3H, CH_3_), 7.44 (t, *J* = 7.5 Hz, 1H, 5-H), 7.48 (d, *J* = 7.5 Hz, 1H, 4-H), 7.76 (d, *J* = 7.4 Hz, 1H, 6-H), 7.78 (s, 1H, 2-H), 7.83 (t, *J* = 8.0 Hz, 1H, 5″-H), 8.20 (br d, *J* = 7.7 Hz, 1H, 6″-H), 8.38 (dd, *J* = 8.2, 2.3 Hz, 1H, 4″-H), 8.55 (t, *J* = 2.0 Hz, 1H, 2″-H), 11.52 (s, 1H, NH); ^13^C NMR (DMSO-d_6_, 100 MHz) δ = 20.86 (CH_3_), 122.01 (C-2″), 125.27 (C-6), 125.37 (C-4″), 126.99 (C-1″), 128.60 (C-2, C-5), 130.91 (C-5″), 132.06 (C-1), 133.51 (C-4), 133.76 (C-6″), 138.11 (C-3), 147.94 (C-3″), 149.93 (C-4′), 150.29 (C-3′), 166.67 (C=O); HRMS (ESI-) calcd. for: C_16_H_11_N_4_O_4_^−^ [M-H]^−^: 323.0775, found: 323.0787.

3-Fluoro-*N*-[4-(3-nitrophenyl)-1,2,5-oxadiazol-3-yl]benzamide (**43**): The reaction of compound **8** (0.41 g (2.00 mmol)), 3-fluorobenzoyl chloride (0.56 g (3.50 mol)) and NaH (60% dispersion in mineral oil) (0.16 g (4.00 mmol)) in dry dimethylformamide (26 mL) gave the crude product. It was recrystallized from dichloromethane, yielding compound **43** as a yellow solid (0.32 g (49%)). m.P.: 174 °C. IR = 3213, 1673, 1590, 1567, 1530, 1442, 1374, 1352, 1206, 1011, 904, 862, 817, 753, 676; ^1^H NMR (DMSO-d_6_, 400 MHz) δ = 7.51 (td, *J* = 8.6, 2.7 Hz, 1H, 4-H), 7.61 (td, *J* = 8.0, 5.7 Hz, 1H, 5-H), 7.78 (dt, *J* = 9.6, 2.0 Hz, 1H, 2-H), 7.81–7.85 (m, 1H, 5″-H, 6-H), 8.23 (dd, *J* = 8.2, 2.0 Hz, 1H, 6″-H), 8.38 (dd, *J* = 8.2, 2.3 Hz, 1H, 4″-H), 8.61 (t, *J* = 2.0 Hz, 1H, 2″-H), 11.80 (br, 1H, NH); ^13^C NMR (DMSO-d_6_, 100 MHz) δ = 114.98 (d, *J* = 23.1 Hz, C-2), 119.66 (d, *J* = 21.1 Hz, C-4), 122.08 (C-2″), 124.37 (d, *J* = 2.9 Hz, C-6), 125.32 (C-4″), 127.04 (C-1″), 130.87 (C-5″), 130.88 (d, *J* = 8.1 Hz, C-5), 133.79 (C-6″), 134.83 (d, *J* = 7.0 Hz, C-1), 147.94 (C-3″), 149.74 (C-4′), 150.53 (C-3′), 161.88 (d, *J* = 245 Hz, C-3), 165.42 (d, *J* = 2.7 Hz, C=O); HRMS (ESI-) calcd. for: C_15_H_8_FN_4_O_4_^−^ [M-H]^−^: 327.0524, found: 327.0537.

*N*-[4-(3-Nitrophenyl)-1,2,5-oxadiazol-3-yl]-3-(trifluoromethyl)benzamide (**44**): The reaction of compound **8** (0.21 g (1.00 mmol)), 3-(trifluoromethyl)benzoyl chloride (0.47 g (2.25 mol)) and NaH (60% dispersion in mineral oil) (0.08 g (3.30 mmol)) in dry dimethylformamide (13 mL) gave the crude product. It was purified by column chromatography (silica gel, CH_2_Cl_2_/EtAc 49:1), yielding compound **44** as a yellow amorphous solid (0.19 g (49%)). IR = 3215, 1674, 1572, 1539, 1513, 1461, 1435, 1373, 1351, 1335, 1281, 1259, 1171, 1074, 924, 821, 758, 726, 685; ^1^H NMR (DMSO-d_6_, 400 MHz) δ = 7.82 (t, *J* = 7.9 Hz, 1H, 5-H), 7.83 (t, *J* = 8.1 Hz, 1H, 5″-H), 8.02–8.07 (m, 1H, 4-H), 8.20–8.26 (m, 2H, 6-H, 6″-H), 8.32 (t, *J* = 2.0 Hz, 1H, 2-H), 8.38 (ddd, *J* = 8.3, 2.3, 1.0 Hz, 1H, 4″-H), 8.56 (t, *J* = 2.0 Hz, 1H, 2″-H), 11.88 (s, 1H, NH); ^13^C NMR (DMSO-d_6_, 100 MHz) δ = 122.08 (C-2″), 123.76 (q, *J* = 273 Hz, CF_3_), 124.79 (q, *J* = 3.9 Hz, C-2), 125.38 (C-4″), 126.91 (C-1″), 129.41 (q, *J* = 32.3 Hz, C-3), 129.43 (q, *J* = 3.7 Hz, C-4), 130.08 (C-5), 130.90 (C-5″), 132.29 (C-6), 133.01 (C-1), 133.86 (C-6″), 147.93 (C-3″), 149.77 (C-4′), 150.02 (C-3′), 165.19 (C=O); HRMS (ESI-) calcd. for: C_16_H_8_F_3_N_4_O_4_^−^ [M-H]^−^: 377.0492, found: 377.0507.

3-Fluoro-*N*-[4-(4-fluorophenyl)-1,2,5-oxadiazol-3-yl]benzamide (**45**): The reaction of compound **7** (0.1 g (0.53 mmol)), 3-fluorobenzoyl chloride (0.11 g (0.69 mmol)) and NaH (60% dispersion in mineral oil) (0.04 g (1.06 mmol)) in dry dimethylformamide (8 mL) gave the crude product. Recrystallization from chloroform yielded compound **45** as a white solid (0.065 mg (42%)). m.P.: 169 °C. IR = 3218, 1675, 1589, 1573, 1503, 1480, 1306, 1224, 1036, 995, 850; ^1^H NMR (CDCl_3_, 400 MHz) δ = 7.38 (br, t, *J* = 8.9 Hz, 2H, 3″-H, 5″-H), 7.53 (td, *J* = 8.4, 2.2 Hz, 1H, 4-H), 7.62 (td, *J* = 7.9, 6.0 Hz, 1H, 5-H), 7.76–7.84 (m, 4H, 2-H, 2″-H, 6-H, 6″-H), 11.46 (br, 1H, NH); ^13^C NMR (CDCl_3_, 100 MHz) δ = 14.96 (d, *J* = 23.0 Hz, C-2), 116.35 (d, *J* = 22.2 Hz, C-3″, C-5″), 119.83 (d, *J* = 20.7 Hz, C-4), 121.62 (d, *J* = 3.1 Hz, C-1″), 124.34 (d, *J* = 3.1 Hz, C-6), 129.84 (d, *J* = 9.2 Hz, C-2″, C-6″), 130.99 (d, *J* = 8.4 Hz, C-5), 134.32 (d, *J* = 6.9 Hz, C-1), 149.87 (C-3′), 150.69 (C-4′), 161.93 (d, *J* = 245 Hz, C-3), 163.45 (d, *J* = 248 Hz, C-4″), 165.20 (d, *J* = 2.3 Hz, CO); HRMS (MALDI) calcd. for: C_15_H_9_F_2_N_3_O_2_: 301.0663; found: 301.0655.

*N*-[4-(4-Chlorophenyl)-1,2,5-oxadiazol-3-yl]-3-fluorobenzamide (**46**): The reaction of compound **6** (0.18 g (0.93 mmol)), 3-fluorobenzoyl chloride (0.19 g (1.21 mmol)) and NaH (60% dispersion in mineral oil) (0.08 g (1.86 mmol)) in dry dimethylformamide (12 mL) gave the crude product. It was recrystallized from dichloromethane, yielding compound **46** as a white solid (0.18 g (62%)). m.P.: 180 °C. IR = 3214, 1669, 1587, 1560, 1501, 1477, 1369, 1307, 1270, 1205, 1093, 995, 955, 903, 845, 821, 801; ^1^H NMR (CDCl_3_, 400 MHz) δ = 7.50–7.56 (m, 1H, 4-H), 7.58–7.66 (m, 3H, 3″-H, 5-H, 5″-H), 7.75–7.83 (m, 4H, 2-H, 2″-H, 6-H, 6″-H), 11.50 (s, 1H, NH); ^13^C NMR (CDCl_3_, 100 MHz) δ = 114.98 (d, *J* = 23.0 Hz, C-2), 119.85 (d, *J* = 20.8 Hz, C-4), 124.08 (C-1″), 124.37 (d, *J* = 2.3 Hz, C-6), 129.15 (C-2″, C-6″), 129.30 (C-3″, C-5″), 130.99 (d, *J* = 7.7 Hz, C-5), 134.28 (d, *J* = 6.9 Hz, C-1), 135.67 (C-4″), 149.93 (C-3′), 150.61 (C-4′), 161.92 (d, *J* = 245 Hz, C-3), 165.16 (d, *J* = 2.3 Hz, CO); HRMS (MALDI) calcd. for: C_15_H_9_ClFN_3_O_2_: 317.0367; found: 317.0363.

*N*-[4-(4-Chlorophenyl)-1,2,5-oxadiazol-3-yl]-3-methylbenzamide (**47**): The reaction of compound **6** (0.09 g (0.47 mmol)), 3-methylbenzoyl chloride (0.09 g (0.61 mmol)) and NaH (60% dispersion in mineral oil) (0.04 g (0.94 mmol)) in dry dimethylformamide (8 mL) gave the crude product. It was recrystallized from dichloromethane, yielding compound **47** as a white solid (0.035 g (23%)). m.P.: 165 °C. IR = 3244, 1677, 1585, 1561, 1478, 1374, 1292, 1092, 994, 839, 821; ^1^H NMR (CDCl_3_, 400 MHz) δ = 2.40 (s, 3H, CH_3_), 7.45 (t, *J* = 1H, 5-H), 7.49 (d, *J* = 7.5 Hz, 1H, 4-H), 7.60 (d, *J* = 8.3 Hz, 2H, 3″-H, 5″-H), 7.75–7.80 (m, 4H, 2-H, 6-H, 2″-H, 6″-H), 11.34 (br, 1H, NH); ^13^C NMR (CDCl_3_, 100 MHz) δ = 20.86 (CH_3_), 124.22 (C-1″), 125.26 (C-6), 128.59 (C-5), 128.61 (C-2), 129.09 (C-2″, C-6″), 129.28 (C-3″, C-5″), 132.04 (C1), 133.47 (C-4), 135.62 (C-4″), 138.16 (C-3), 150.23 (C-3′), 150.74 (C-4′), 166.54 (CO); HRMS (MALDI) calcd. for: C_16_H_12_ClN_3_O_2_: 313.0618; found: 313.0596.

*N*-[4-(4-Chlorophenyl)-1,2,5-oxadiazol-3-yl]-3-(trifluoromethyl)benzamide (**48**): The reaction of compound **6** (0.09 g (0.47 mmol)), 3-(trifluoromethyl)benzoyl chloride (0.13 g (0.61 mmol)) and NaH (60% dispersion in mineral oil) (0.04 g (0.94 mmol)) in dry dimethylformamide (8 mL) gave the crude product. It was recrystallized from dichloromethane, yielding compound **48** as a white solid (0.015 g (9%)). NMR data were in accordance with the literature data [34].

*N*-[4-(4-Fluorophenyl)-1,2,5-oxadiazol-3-yl]-3-methylbenzamide (**49**): The reaction of compound **7** (0.17 g (0.97 mmol)), 3-methylbenzoyl chloride (0.20 g (1.26 mmol)) and NaH (60% dispersion in mineral oil) (0.08 g (1.94 mmol)) in dry dimethylformamide (12 mL) gave the crude product. It was purified by column chromatography (silica gel, CH_2_Cl_2_), yielding compound **49** as a white amorphous solid (0.059 g (21%)). IR = 3281, 1672, 1600, 1572, 1530, 1484, 1414, 1377, 1283, 1235, 994, 888, 840; ^1^H NMR (CDCl_3_, 400 MHz) δ = 2.43 (s, 3H, CH_3_), 7.14–7.20 (m, 2H, 3″-H, 5″-H), 7.40 (t, *J* = 7.7 Hz, 1H, 5-H), 7.44 (br, d, *J* = 7.7 Hz, 1H, 4-H), 7.64 (br, d, *J* = 7.7 Hz, 1H, 6-H), 7.69 (br, d, 1H, 2-H), 7.70–7.73 (m, 2H, 2″-H, 6″-H), 8.21 (br, s, 1H, NH); ^13^C NMR (CDCl_3_, 100 MHz) δ = 1.32 (CH_3_), 116.49 (d, *J* = 22.2 Hz, C-3″, C-5″), 121.67 (C-1″), 124.51 (C-6), 128.40 (C-2), 128.95 (C-5), 129.67 (d, *J* = 8.4 Hz, C-2″, C-6″), 131.79 (C-1), 134.11 (C-4), 139.20 (C-3), 148.89 (C-3′), 149.63 (C-4′), 164.08 (d, *J* = 252 Hz, C-4″), 165.70 (CO); HRMS (MALDI) calcd. for C_16_H_12_FN_3_O_2_: 297.0914; found: 297.0922.

*N*-[4-(4-Fluorophenyl)-3-(trifluoromethyl)-1,2,5-oxadiazol-3-yl]benzamide (**50**): The reaction of compound **7** (0.17 g (0.97 mmol)), 3-(trifluoromethyl)benzoyl chloride (0.26 g (1.26 mmol)) and NaH (60% dispersion in mineral oil) (0.08 g (1.94 mmol)) in dry dimethylformamide (12 mL) gave the crude product. It was recrystallized from dichloromethane, yielding compound **50** as a light yellow solid (0.081 g (24%)). m.P.: 251 °C. IR = 3435, 1611, 1541, 1506, 1477, 1370, 1323, 1261, 1127, 841; ^1^H NMR (CDCl_3_, 400 MHz) δ = 7.32 (br, t, *J* = 8.6 Hz, 2H, 3″-H, 5″-H), 7.60 (t, *J* = 7.5 Hz, 1H, 5-H), 7.71 (d, *J* = 7.3 Hz, 1H, 4-H), 8.33–8.41 (m, 4H, 2-H, 2″-H, 6-H, 6″-H); ^13^C NMR (CDCl_3_, 100 MHz) δ = 115.45 (d, *J* = 21.5 Hz, C-3″, C-5″), 124.55 (q, *J* = 272 Hz, CF3), 124.56 (d, *J* = 3.0 Hz, C-1″), 124.73 (q, *J* = 3.8 Hz, C-2), 125.46 (q, *J* = 3.8 Hz, C-4), 128.31 (q, *J* = 30.7 Hz, C-3), 128.66 (C-5), 130.13 (C-2″, C-6″), 132.11 (C-6), 142.00 (C-1), 149.46 (C-4′), 158.99 (C-3′), 162.80 (d, *J* = 247 Hz, C-4″), 167.70 (CO); HRMS (MALDI) calcd. for C_16_H_9_F_4_N_3_O_2_: 351.0631; found: 351.0631.

*N*-[4-(3-Ethoxy-4-methoxyphenyl)-1,2,5-oxadiazol-3-yl]-3-methylbenzamide (**51**): The reaction of compound **25** (0.24 g (1.00 mmol)) with 3-methylbenzoyl chloride (0.31 g (2.00 mmol)) and NaH (60% dispersion in mineral oil) (0.16 g (4.00 mmol)) in dry dimethylformamide (25 mL) for 48 h gave the crude product. It was recrystallized from dichloromethane, yielding compound **51** as a yellow solid (0.13 g (35%)). m.P.: 280 °C. IR = 2977, 1592, 1507, 1470, 1443, 1361, 1273, 1251, 1221, 1182, 1143, 1022, 937, 876, 858, 810; ^1^H NMR (DMSO-d_6_, 400 MHz) δ = 1.34 (t, *J* = 7.0 Hz, 3H, CH_3_), 2.35 (s, 3H, ArCH_3_), 3.82 (s, 3H, OCH_3_), 4.07 (q, *J* = 7.0 Hz, 2H, OCH_2_), 7.08 (d, *J* = 8.6 Hz, 1H, 5″-H), 7.19 (d, *J* = 7.4 Hz, 1H, 4-H), 7.24 (t, *J* = 7.4, 1H, 5-H), 7.89 (dd, *J* = 8.6, 1.2 Hz, 1H, 6″-H), 7.97 (d, *J* = 7.4 Hz, 1H, 6-H), 8.01 (s, 1H, 2-H), 8.26 (d, *J* = 1.2 Hz, 1H, 2″-H); ^13^C NMR (DMSO-d_6_, 100 MHz) δ = 14.78 (CH_3_), 21.16 (ArCH_3_), 55.47 (OCH_3_), 63.62 (OCH_2_), 111.71 (C-5″), 112.34 (C-2″), 120.60 (C-1″), 120.65 (C-6″), 125.60 (C-6), 127.18 (C-5), 128.97 (C-2), 129.65 (C-4), 136.12 (C-3), 140.98 (C-1), 147.47 (C-3″), 149.62 (C-4′), 149.85 (C-4″), 158.80 (C-3′), 169.69 (C=O); HRMS (EI+) calcd. for C_19_H_19_N_3_O_4_: 353.1375; found: 353.1375.

*N*-[4-(4-Ethoxy-3-methoxyphenyl)-1,2,5-oxadiazol-3-yl]-3-methylbenzamide (**52**): The reaction of compound **26** (0.22 g (0.91 mmol)) with 3-methylbenzoyl chloride (0.28 g (1.82 mmol)) and NaH (60% dispersion in mineral oil) (0.15 g (3.64 mmol)) in dry dimethylformamide (23 mL) for 48 h gave the crude product. It was recrystallized from dichloromethane, yielding compound **52** as a light yellow solid (0.13 g (39%)). m.P.: 260 °C. IR = 2979, 1663, 1590, 1540, 1470, 1414, 1364, 1275, 1247, 1222, 1181, 1143, 1086, 1032, 995, 925, 860, 809; ^1^H NMR (DMSO-d_6_, 400 MHz) δ = 1.35 (t, *J* = 7.0 Hz, 3H, CH_3_), 2.35 (s, 3H, ArCH_3_), 3.80 (s, 3H, OCH_3_), 4.07 (q, *J* = 7.0 Hz, 2H, OCH_2_), 7.07 (d, *J* = 8.4 Hz, 1H, 5″-H), 7.22 (d, *J* = 7.4 Hz, 1H, 4-H), 7.27 (t, *J* = 7.4, 1H, 5-H), 7.82 (dd, *J* = 8.4, 1.7 Hz, 1H, 6″-H), 7.95 (d, *J* = 7.4 Hz, 1H, 6-H), 7.99 (s, 1H, 2-H), 8.12 (d, *J* = 1.7 Hz, 1H, 2″-H), 11.33 (br, 1H, NH); ^13^C NMR (DMSO-d_6_, 100 MHz) δ = 14.69 (CH_3_), 21.09 (ArCH_3_), 55.27 (OCH_3_), 63.67 (OCH_2_), 111.22 (C-2″), 112.53 (C-5″), 120.04 (C-1″), 120.63 (C6″), 125.52 (C-6), 127.42 (C-5), 128.91 (C-2), 130.17 (C-4), 136.41 (C-3), 139.74 (C-1), 148.43 (C-3″), 149.12 (C-4″), 149.87 (C-4′), 157.54 (C-3′), 169.28 (C=O); HRMS (EI+) calcd. for C_19_H_19_N_3_O_4_: 353.1375; found: 353.1376.

##### General Procedure for the Preparation of Compounds **53** and **54**

3-Chloro-4-methoxybenzoic acid, *N*-hydroxy succinimide and DCC were dissolved in dry tetrahydrofuran. The reaction mixture was stirred at room temperature overnight. After that, dimethylformamide was added and the mixture was stirred for 15 min. The precipitate was filtered and washed with tetrahydrofuran. The filtrate was evaporated in vacuo and the residue was dissolved in dichloromethane. The organic phase was washed three times with aqueous NaHCO_3_. The aqueous phases were combined and extracted twice with dichloromethane. The combined organic phases were dried over anhydrous sodium sulfate, filtered and the solvent was evaporated in vacuo, yielding the *N*-(aroyloxy)succinimide. NaH (60% dispersion in mineral oil) was suspended in dry dimethylformamide and cooled in an ice bath to 0 °C. The corresponding 3-aminofurazan was added and the reaction mixture was stirred at 0 °C for 20 min. After that, a solution of the *N*-(aroyloxy)succinimide in dry dimethylformamide was added via a dropping funnel. The reaction mixture was heated to 60 °C overnight. After completion, the mixture was cooled to 0 °C and water was added. The aqueous phase was extracted three times with dichloromethane. The organic phases were combined, dried over anhydrous sodium sulfate and filtered. The solvent was evaporated in vacuo, yielding the crude products, which were recrystallized.

3-Chloro-4-methoxy-*N*-[4-(4-nitrophenyl)-1,2,5-oxadiazol-3-yl]benzamide (**53**): The reaction of 3-chloro-4-methoxybenzoic acid (0.4 g (2.15 mmol)) with *N*-hydroxy succinimide (0.26 g (2.26 mmol)) and DCC (0.44 g (2.15 mmol)) in dry tetrahydrofuran (15 mL) gave the *N*-(aroyloxy)succinimide. A suspension of NaH (60% dispersion in mineral oil) (0.08 g (3.30 mmol)) in dry dimethylformamide (13 mL) was prepared. Reaction of the latter with the *N*-(aroyloxy)succinimide and the aminofurazan **21** (0.21 g (1.00 mmol)) gave the crude product. The latter was purified by recrystallization from dichloromethane, yielding compound **53** as a yellow solid (0.2 g (52%)). m.P.: 261 °C. IR = 3218, 1671, 1601, 1524, 1466, 1417, 1350, 1280, 1058, 1012, 856, 764, 690; ^1^H NMR (DMSO-d_6_, 400 MHz) δ = 3.93 (s, 3H, OCH_3_), 7.26 (t, *J* = 8.6 Hz, 1H, 5-H), 7.99 (dd, *J* = 8.6, 1.9 Hz, 1H, 6-H), 8.07 (d, *J* = 1.9 Hz, 1H, 2-H), 8.19 (d, *J* = 8.7 Hz, 2H, 2″-H, 6″-H), 8.34 (d, *J* = 8.7 Hz, 2H, 3″-H, 5″-H), 11.57 (br, 1H, NH); ^13^C NMR (DMSO-d_6_, 100 MHz) δ = 56.46 (OCH_3_), 112.27 (C-5), 120.72 (C-1), 123.97 (C-3″, C-5″), 127.88 (C-3), 128.77 (C-2″, C-6″), 129.01 (C-6), 129.87 (C-2), 132.77 (C-1″), 148.29 (C-4″), 149.74 (C-4′), 153.21 (C-3′), 157.15 (C-4), 165.62 (C=O); HRMS (ESI-) calcd. for: C_16_H_10_ClN_4_O_5_^−^ [M-H]^−^: 373.0334, found: 373.0347.

3-Chloro-4-methoxy-*N*-[4-(3-nitrophenyl)-1,2,5-oxadiazol-3-yl]benzamide (**54**): The reaction of 3-chloro-4-methoxybenzoic acid (0.4 g (2.15 mmol)) with *N*-hydroxy succinimide (0.26 g (2.26 mmol)) and DCC (0.44 g (2.15 mmol)) in dry tetrahydrofuran (15 mL) gave the *N*-(aroyloxy)succinimide. A suspension of NaH (60% dispersion in mineral oil) (0.08 g (3.30 mmol)) in dry dimethylformamide (13 mL) was prepared. Reaction of the latter with the *N*-(aroyloxy)succinimide and the aminofurazan **8** (0.21 g (1.00 mmol)) gave the crude product. The latter was purified by recrystallization from dichloromethane, yielding compound **54** as a yellow solid (0.14 g (38%)). m.P.: 248 °C. IR = 3444, 1670, 1601, 1516, 1461, 1378, 1351, 1308, 1273, 1063, 1019, 815, 755, 731, 679; ^1^H NMR (DMSO-d_6_, 400 MHz) δ = 3.94 (s, 3H, OCH_3_), 7.25 (t, *J* = 8.7 Hz, 1H, 5-H), 7.81 (t, *J* = 8.0 Hz, 1H, 5″-H), 8.02 (dd, *J* = 8.7, 2.2 Hz, 1H, 6-H), 8.12 (d, *J* = 2.2 Hz, 1H, 2-H), 8.31–8.37 (m, 2H, 4″-H, 6″-H), 8.96 (t, *J* = 2.0 Hz, 1H, 2″-H), 11.56 (br, 1H, NH); ^13^C NMR (DMSO-d_6_, 100 MHz) δ = 56.45 (OCH_3_), 112.20 (C-5), 120.75 (C-1), 122.19 (C-2″), 124.90 (C-4″), 127.98 (C-1″), 128.16 (C-3), 128.92 (C-6), 129.89 (C-2), 130.61 (C-5″), 133.68 (C-6″), 147.87 (C-3″), 149.23 (C-4′), 153.20 (C-3′), 157.10 (C-4), 165.96 (C=O); HRMS (ESI-) calcd. for: C_16_H_10_ClN_4_O_5_^−^ [M-H]^−^: 373.0334, found: 373.0351.

#### 3.2.10. Preparation of Aniline Derivatives

##### General Procedure for the Preparation of Compounds **55**–**63**

Tin powder was purified before use. Therefore, it was mixed with 10% aqueous sodium hydroxide and stirred intensely for 10 min. The precipitate was filtered and washed with water to neutralize the pH. Then, the tin powder was dried in a desiccator. The corresponding amide, Sn(0), 6*N* HCl and ethanol were added to a round-bottom flask and heated to 70 °C for 1 h. After completion, the reaction mixture was cooled to room temperature and filtered with celite. The filtrate was poured into a saturated aqueous NaHCO_3_ solution. The aqueous phase was extracted three times with ethyl acetate. The organic phases were combined, dried over anhydrous sodium sulfate and filtered, and the solvent was evaporated in vacuo, yielding the crude products, which were either recrystallized or purified by column chromatography.

*N*-[4-(4-Aminophenyl)-1,2,5-oxadiazol-3-yl]benzamide (**55**): The reaction of compound **37** (0.08 g (0.25 mmol)) with Sn(0) (0.15 g (1.25 mmol)) in 6*N* HCl (1 mL) and ethanol (1 mL) gave the crude product. It was recrystallized from dichloromethane, yielding compound **55** as a pale brown solid (0.056 g (76%)). m.P.: 169 °C. IR = 3423, 3239, 1659, 1609, 1535, 1484, 1381, 1302, 1184, 994, 837, 716, 657; ^1^H NMR (DMSO-d_6_, 400 MHz) δ = 5.67 (s, 2H, NH_2_), 6.60 (d, *J* = 8.5 Hz, 2H, 3″-H, 5″-H), 7.49 (d, *J* = 8.5 Hz, 2H, 2″-H, 6″-H), 7.56 (t, *J* = 7.5 Hz, 2H, 3-H, 5-H), 7.65 (t, *J* = 7.2 Hz, 1H, 4-H), 8.01 (d, *J* = 7.2 Hz, 2H, 2-H, 6-H), 11.11 (br, 1H, NH); ^13^C NMR (DMSO-d_6_, 100 MHz) δ = 111.23 (C-1″), 113.61 (C-3″, C-5″), 127.99 (C-2, C-6), 128.32 (C-2″, C-6″), 128.66 (C-3, C-5), 132.49 (C-4), 132.85 (C-1), 150.15 (C-3′), 151.22 (C-4″), 151.53 (C-4′), 166.75 (C=O); HRMS (ESI+) calcd. for C_15_H_13_N_4_O_2_^+^ [M+H]^+^: 281.1039; found: 281.1028.

*N*-[4-(4-Aminophenyl)-1,2,5-oxadiazol-3-yl]-3-methylbenzamide (**56**): The reaction of compound **38** (0.32 g (1.00 mmol)) with Sn(0) (0.59 g (5.00 mmol)) in 6*N* HCl (4 mL) and ethanol (4 mL) gave the crude product. It was recrystallized from dichloromethane, yielding compound **56** as a white solid (0.12 g (41%)). m.P.: 159 °C. IR = 3409, 3340, 3234, 1661, 1609, 1566, 1486, 1382, 1302, 1183, 992, 938, 884, 835, 743, 660; ^1^H NMR (DMSO-d_6_, 400 MHz) δ = 2.41 (s, 3H, CH_3_), 5.68 (s, 2H, NH_2_), 6.60 (d, *J* = 8.7 Hz, 2H, 3″-H, 5″-H), 7.43–7.50 (m, 4H, 2″-H, 4-H, 5-H, 6″-H), 7.78–7.81 (m, 2H, 2-H, 6-H), 11.01 (s, 1H, NH); ^13^C NMR (DMSO-d_6_, 100 MHz) δ = 20.90 (CH_3_), 111.01 (C-1″), 113.63 (C-3″, C-5″), 125.07 (C-6), 128.27 (C-2″, C-6″), 128.46 (C-2), 128.63 (C-5), 132.27 (C-1), 133.32 (C-4), 138.16 (C-3), 149.62 (C-3′), 151.29 (C-4″), 151.60 (C-4′), 166.66 (C=O); HRMS (ESI+) calcd. for C_16_H_13_N_4_O_2_^+^ [M+H]^+^: 295.1195; found: 295.1185.

*N*-[4-(4-Aminophenyl)-1,2,5-oxadiazol-3-yl]-3-fluorobenzamide (**57**): The reaction of compound **39** (0.4 g (1.23 mmol)) with Sn(0) (0.73 g (6.13 mmol)) in 6*N* HCl (4.9 mL) and ethanol (4.9 mL) gave the crude product. It was recrystallized from dichloromethane, yielding compound **57** as a white solid (0.19 g (52%)). m.P.: 189 °C. IR = 3410, 3239, 1661, 1610, 1588, 1484, 1381, 1302, 1184, 994, 887, 836, 754, 656; ^1^H NMR (DMSO-d_6_, 400 MHz) δ = 5.69 (s, 2H, NH_2_), 6.61 (d, *J* = 8.6 Hz, 2H, 3″-H, 5″-H), 7.45 (d, *J* = 8.6 Hz, 2H, 2″-H, 6″-H), 7.54 (td, *J* = 8.3, 2.4 Hz, 1H, 4-H), 7.64 (td, *J* = 8.0, 5.8 Hz, 1H, 5-H), 7.79 (dt, *J* = 9.8, 2.1 Hz, 1H, 2-H), 7.84 (d, *J* = 7.8 Hz, 1H, 6-H), 11.19 (s, 1H, NH); ^13^C NMR (DMSO-d_6_, 100 MHz) δ = 110.90 (C-1″), 113.68 (C-3″, C-5″), 114.83 (d, *J* = 23.1 Hz, C-2), 119.73 (d, *J* = 21.1 Hz, C-4), 124.21 (d, *J* = 2.9 Hz, C-6), 128.32 (C-2″, C-6″), 131.04 (d, *J* = 8.0 Hz, C-5), 134.57 (d, *J* = 6.9 Hz, C-1), 149.38 (C-3′), 151.34 (C-4″), 151.52 (C-4′), 161.99 (d, *J* = 245 Hz, C-3), 165.29 (d, *J* = 2.6 Hz, C=O); HRMS (ESI+) calcd. for C_15_H_12_FN_4_O_2_^+^ [M+H]^+^: 299.0944; found: 299.0936.

*N*-[4-(4-Aminophenyl)-1,2,5-oxadiazol-3-yl]-3-(trifluoromethyl)benzamide (**58**): The reaction of compound **40** (0.79 g (2.08 mmol)) with Sn(0) (1.236 g (10.40 mmol)) in 6*N* HCl (4.2 mL) and ethanol (4.2 mL) gave the crude product. It was recrystallized from dichloromethane, yielding compound **58** as a light yellow solid (0.32 g (43%)). m.P.: 154 °C. IR = 3252, 1668, 1619, 1537, 1492, 1437, 1336, 1260, 1183, 1136, 1074, 994, 837, 756, 677; ^1^H NMR (DMSO-d_6_, 400 MHz) δ = 5.70 (s, 2H, NH_2_), 6.61 (d, *J* = 8.6 Hz, 2H, 3″-H, 5″-H), 7.46 (d, *J* = 8.6 Hz, 2H, 2″-H, 6″-H), 7.84 (t, *J* = 7.8 Hz, 1H, 5-H), 8.05 (d, *J* = 7.7 Hz, 1H, 4-H), 8.29 (d, *J* = 7.9 Hz, 1H, 6-H), 8.33 (s, 1H, 2-H), 11.37 (s, 1H, NH); ^13^C NMR (DMSO-d_6_, 100 MHz) δ = 110.86 (C-1″), 113.66 (C-3″, C-5″), 123.81 (q, *J* = 273 Hz, CF_3_), 124.60 (q, *J* = 4.0 Hz, C-2), 128.35 (C-2″, C-6″), 129.29 (q, *J* = 3.8 Hz, C-4), 129.48 (q, *J* = 32.3 Hz, C-3), 130.16 (C-5), 132.15 (C-6), 133.20 (C-1), 149.28 (C-3′), 151.36 (C-4″), 151.44 (C-4′), 165.15 (C=O); HRMS (ESI+) calcd. for C_16_H_12_F_3_N_4_O_2_^+^ [M+H]^+^: 349.0912; found: 349.0902.

*N*-[4-(4-Aminophenyl)-1,2,5-oxadiazol-3-yl]-3-chloro-4-methoxybenzamide (**59**): The reaction of compound **53** (0.8 g (2.13 mmol)) with Sn(0) (1.27 g (10.65 mmol)) in 6*N* HCl (8.5 mL) and ethanol (8.5 mL) gave the crude product. It was recrystallized from dichloromethane, yielding compound **59** as a white solid (0.12 g (16%)). m.P.: 250 °C. IR = 3456, 3370, 3212, 1663, 1635, 1608, 1487, 1382, 1310, 1275, 1063, 935, 831, 672; ^1^H NMR (DMSO-d_6_, 400 MHz) δ = 3.97 (s, 3H, OCH_3_), 5.68 (s, 2H, NH_2_), 6.59 (d, *J* = 8.7 Hz, 2H, 3″-H, 5″-H), 7.34 (d, *J* = 8.8 Hz, 1H, 5-H), 7.43 (d, *J* = 8.7 Hz, 2H, 2″-H, 6″-H), 7.99 (dd, *J* = 8.7, 2.2 Hz, 1H, 6-H), 8.07 (d, *J* = 2.2 Hz, 1H, 2-H), 11.02 (s, 1H, NH); ^13^C NMR (DMSO-d_6_, 100 MHz) δ = 56.61 (OCH_3_), 111.04 (C-1″), 112.68 (C-5), 113.63 (C-3″, C-5″), 121.21 (C-1), 125.23 (C-3), 128.27 (C-2″, C-6″), 128.90 (C-6), 129.61 (C-2), 149.61 (C-3′), 151.28 (C-4″), 151.51 (C-4′), 157.89 (C-4), 164.82 (C=O); HRMS (ESI+) calcd. for C_16_H_14_ClN_4_O_3_^+^ [M+H]^+^: 345.0754; found: 345.0744.

*N*-[4-(3-Aminophenyl)-1,2,5-oxadiazol-3-yl]benzamide (**60**): The reaction of compound **41** (0.33 g (1.06 mmol)) with Sn(0) (0.63 g (5.30 mmol)) in 6*N* HCl (4.2 mL) and ethanol (4.2 mL) gave the crude product. It was recrystallized from dichloromethane, yielding compound **60** as a beige solid (0.19 g (64%)). m.P.: 178 °C. IR = 3401, 1674, 1600, 1562, 1519, 1476, 1383, 1275, 1008, 875, 801, 707, 691, 653; ^1^H NMR (DMSO-d_6_, 400 MHz) δ = 5.38 (s, 2H, NH_2_), 8.69 (br dd, *J* = 8.1, 2.4 Hz, 1H, 4″-H), 6.84 (br dd, *J* = 7.7 Hz, 1H, 6″-H), 6.98 (t, *J* = 2.0 Hz, 1H, 2″-H), 7.10 (t, *J* = 7.8 Hz, 1H, 5″-H), 7.57 (t, *J* = 7.5 Hz, 2H, 3-H, 5-H), 7.66 (t, *J* = 7.4 Hz, 1H, 4-H), 7.95–8.00 (m, 2H, 2-H, 6-H), 11.15 (s, 1H, NH); ^13^C NMR (DMSO-d_6_, 100 MHz) δ = 112.13 (C-2″), 114.07 (C-6″), 116.14 (C-4″), 125.35 (C-1″), 128.02 (C-2, C-6), 128.70 (C-3, C-5), 129.61 (C-5″), 132.29 (C-1), 132.72 (C-4), 149.34 (C-3″), 150.03 (C-3′), 152.08 (C-4′), 166.50 (C=O); HRMS (ESI+) calcd. for C_15_H_13_N_4_O_2_^+^ [M+H]^+^: 281.1039; found: 281.1028.

*N*-[4-(3-Aminophenyl)-1,2,5-oxadiazol-3-yl]-3-methylbenzamide (**61**): The reaction of compound **42** (0.43 g (1.31 mmol)) with Sn(0) (0.78 g (6.55 mmol)) in 6*N* HCl (5.2 mL) and ethanol (5.2 mL) gave the crude product. It was recrystallized from dichloromethane, yielding compound **61** as a pale brown solid (0.15 g (38%)). m.P.: 172 °C. IR = 3402, 3319, 1673, 1602, 1568, 1522, 1473, 1384, 1285, 1198, 1007, 889, 801, 742, 696, 655; ^1^H NMR (DMSO-d_6_, 400 MHz) δ = 2.39 (s, 3H, CH_3_), 5.38 (s, 2H, NH_2_), 6.69 (dd, *J* = 8.1, 2.3, 1.0 Hz, 1H, 4″-H), 6.83 (dt, *J* = 7.7, 1.3 Hz, 1H, 6″-H), 6.98 (t, *J* = 2.0 Hz, 1H, 2″-H), 7.10 (t, *J* = 7.9 Hz, 1H, 5″-H), 7.44 (t, *J* = 7.5 Hz, 1H, 5-H), 7.46–7.49 (m, 1H, 4-H), 7.77 (dt, *J* = 7.0, 2.0 Hz, 1H, 6-H), 7.80 (br s, 1H, 2-H), 11.09 (s, 1H, NH); ^13^C NMR (DMSO-d_6_, 100 MHz) δ = 20.89 (CH_3_), 112.14 (C-2″), 114.08 (C-6″), 116.13 (C-4″), 125.16 (C-6), 125.39 (C-1″), 128.52 (C-2), 128.58 (C-5), 129.60 (C-5″), 132.26 (C-1), 133.30 (C-4), 138.10 (C-3), 149.33 (C-3″), 150.07 (C-3′), 152.10 (C-4′), 166.56 (C=O); HRMS (ESI+) calcd. for C_16_H_15_N_4_O_2_^+^ [M+H]^+^: 295.1195; found: 295.1185.

*N*-[4-(3-Aminophenyl)-1,2,5-oxadiazol-3-yl]-3-fluorobenzamide (**62**): The reaction of compound **43** (0.32 g (0.98 mmol)) with Sn(0) (0.58 g (4.90 mmol)) in 6*N* HCl (3.9 mL) and ethanol (3.9 mL) gave the crude product. It was recrystallized from dichloromethane, yielding compound **62** as a pale brown solid (0.1 g (36%)). m.P.: 163 °C. IR = 3391, 3326, 1677, 1590, 1563, 1521, 1477, 1384, 1323, 1289, 1205, 1007, 951, 860, 801, 746, 692, 657; ^1^H NMR (DMSO-d_6_, 400 MHz) δ = 5.38 (s, 2H, NH_2_), 6.69 (br dd, *J* = 8.1, 2.3 Hz, 1H, 4″-H), 6.86 (br d, *J* = 7.7 Hz, 1H, 6″-H), 6.99 (t, *J* = 2.0 Hz, 1H, 2″-H), 7.11 (t, *J* = 7.8 Hz, 1H, 5″-H), 7.52 (td, *J* = 8.1, 2.4 Hz, 1H, 4-H), 7.62 (td, *J* = 8.0, 5.8 Hz, 1H, 5-H), 7.78 (dt, *J* = 9.8, 2.1 Hz, 1H, 2-H), 7.84 (br d, *J* = 7.7 Hz, 1H, 6-H), 11.31 (br, 1H, NH); ^13^C NMR (DMSO-d_6_, 100 MHz) δ = 112.13 (C-2″), 114.16 (C-6″), 114.89 (d, *J* = 23.2 Hz, C-2), 116.12 (C-4″), 119.58 (d, *J* = 21.1 Hz, C-4), 124.29 (d, *J* = 2.9 Hz, C-6), 125.35 (C-1″), 129.62 (C-5″), 130.91 (d, *J* = 8.0 Hz, C-5), 134.80 (d, *J* = 7.1 Hz, C-1), 149.34 (C-3″), 150.08 (C-3′), 151.97 (C-4′), 161.94 (d, *J* = 245 Hz, C-3), 165.29 (d, *J* = 2.7 Hz, C=O); HRMS (ESI+) calcd. for C_15_H_12_FN_4_O_2_^+^ [M+H]^+^: 299.0944; found: 299.0935.

*N*-[4-(3-Aminophenyl)-1,2,5-oxadiazol-3-yl]-3-(trifluoromethyl)benzamide (**63**): The reaction of compound **44** (0.26 g (0.69 mmol)) with Sn(0) (0.41 g (3.43 mmol)) in 6*N* HCl (2.7 mL) and ethanol (2.7 mL) gave the crude product. It was recrystallized from dichloromethane, yielding compound **63** as an amorphous white solid (0.03 g (12%)). IR = 3420, 1703, 1616, 1541, 1470, 1369, 1325, 1251, 1174, 1126, 1069, 868, 756, 688; ^1^H NMR (DMSO-d_6_, 400 MHz) δ = 5.27 (s, 2H, NH_2_), 6.67 (br d, *J* = 7.9 Hz, 1H, 4″-H), 7.10 (br d, *J* = 7.9 Hz, 1H, 5″-H), 7.23 (br s, 2H, 2″-H, 6″-H), 7.71 (br t, *J* = 8.0 Hz, 1H, 5-H), 7.86 (br d, *J* = 7.9 Hz, 1H, 4-H), 8.35 (br d, *J* = 8.0 Hz, 1H, 6-H), 8.39 (s, 1H, 2-H), 11.53 (br, 1H, NH); ^13^C NMR (DMSO-d_6_, 100 MHz) δ = 112.67 (C-2″), 114.98 (C-6″), 115.60 (C-4″), 124.21 (q, *J* = 273 Hz, CF_3_), 124.73 (q, *J* = 3.5 Hz, C-2), 126.95 (C-1″), 127.13 (q, *J* = 3.5 Hz, C-4), 128.76 (q, *J* = 32.2 Hz, C-3), 129.13 (C-5″), 129.26 (C-5), 132.18 (C-6), 137.97 (C-1), 148.94 (C-3″), 151.32 (C-3′), 154.68 (C-4′), 166.25 (C=O); HRMS (ESI+) calcd. for C_16_H_12_F_3_N_4_O_2_^+^ [M+H]^+^: 349.0912; found: 349.0902.

### 3.3. Biological Tests

#### 3.3.1. In Vitro Microplate Assay against *P. falciparum*

In vitro activity against the erythrocytic stages of *P. falciparum* was determined using a ^3^H-hypoxanthine incorporation assay [35,36], using the drug sensitive NF54 strain [37]. Compounds were dissolved in DMSO at 10 mg/mL and added to parasite cultures incubated in RPMI 1640 medium without hypoxanthine, supplemented with HEPES (5.94 g/L), NaHCO_3_ (2.1 g/L, neomycin (100 U/mL), Albumax (5 g/L) and washed human red blood cells A+ at 2.5% hematocrit (0.3% parasitemia). Serial drug dilutions of 11 3-fold dilution steps covering a range from 100 to 0.002 µg/mL were prepared. The 96-well plates were incubated in a humidified atmosphere at 37 °C, 4% CO_2_, 3% O_2_ and 93% N_2_. After 48 h, 0.05 mL of ^3^H-hypoxanthine (=0.5 µCi) was added to each well of the plate. The plates were incubated for a further 24 h under the same conditions. The plates were then harvested with a Betaplate cell harvester (Wallac, Zurich, Switzerland). The red blood cells were transferred onto a glass fiber filter and washed with distilled water. The dried filters were inserted into a plastic foil with 10 mL of scintillation fluid and counted in a Betaplate liquid scintillation counter (Wallac, Zurich, Switzerland). The IC_50_ values were calculated from sigmoidal inhibition curves by linear regression [38] using Microsoft Excel. Chloroquine (Sigma C6628) was used as control.

#### 3.3.2. In Vitro Cytotoxicity with L-6 Cells

Assays were performed in 96-well microtiter plates, each well containing 0.1 mL of RPMI 1640 medium supplemented with 1% L-glutamine (200 mM) and 10% fetal bovine serum and 4000 L-6 cells (a primary cell line derived from rat skeletal myoblasts, ATCC CRL-1458™) [39,40]. Serial drug dilutions of 11 3-fold dilution steps covering a range from 100 to 0.002 μg/mL were prepared. After 70 h of incubation, the plates were inspected under an inverted microscope to assure the growth of the controls and sterile conditions. Then, 0.01 mL resazurin solution (resazurin, 12.5 mg in 100 mL double-distilled water) was added to each well, and the plates were incubated for another 2 h. The plates were read with a Spectramax Gemini XS microplate fluorometer (Molecular Devices Cooperation, Sunnyvale, CA, USA) using an excitation wavelength of 536 nm and an emission wavelength of 588 nm. The IC_50_ values were calculated by linear regression [38] from the sigmoidal dose inhibition curves using SoftmaxPro software Version 8.2.1 (Molecular Devices Cooperation, Sunnyvale, CA, USA). Podophyllotoxin (Sigma P4405) was used as control.

#### 3.3.3. Cytochrome P450 3A4 Inhibition

The CYP3A4 inhibition assay was performed using 96-well white plates (Greiner Bio-One) at a pH of 7.4. Stock solutions (4 mM) of test compounds were prepared in DMSO, and stock solution of the standard ketoconazole (5 mM) was prepared in acetonitrile. Stock solutions were further diluted to a final concentration of 20 µM using water (HPLC grade). The luciferin IPA stock solution (3 mM) was diluted to a final concentration of 0.3 mM using water (HPLC grade). The CYP3A4 reaction mixture was prepared by mixing water (HPLC grade) with potassium phosphate buffer (1 M), luciferin IPA (0.3 mM) and CYP3A4 membrane (1 pmol/µL). The control reaction mixture was prepared using water (HPLC grade), potassium phosphate buffer (1 M), luciferin IPA (0.3 mM) and membrane without CYP activity (1 pmol/µL). Solutions A and B of the NADPH regeneration system were mixed and HPLC grade water was added. The reconstituted luciferin detection reagent was prepared by mixing the reconstituted buffer with esterase with the luciferin detection reagent. Then, solutions of test compounds and standard were added to the wells of the 96-well white plate; each was tested in triplicate. The CYP3A4 reaction mixture was added to each well and the plate was incubated for 10 min at room temperature. After that, the NADPH regeneration system was added, inducing the reaction followed by an incubation time of 10 min at ambient temperature. By adding the reconstituted luciferin detection reagent, the reaction was terminated and a luminescent signal was formed. Luminescence was measured by a SpectraMax M3 UV plate reader (Molecular Devices). The relative light units (RLU) were received from a calibration curve with beetle luciferin. Ketoconazole (100% enzyme inhibition) was used as standard [41]. The CYP3A4 inhibition (%) was calculated from the RLU.

#### 3.3.4. Aqueous Solubility (Commissioned Work Performed by Bienta, Kiew, Ukraine)

To determine the solubility of compounds in PBS pH 7.4 with 1% of DMSO at room temperature, DMSO (DMSO Chromasolv Plus, HPLC grade ≥ 99.7% Sigma Aldrich, St. Louis, MO, USA) stock solution of the reference compounds, highly soluble 2′-deoxy-5-fluorouridine (Enamine Ltd., Kyiv, Ukraine) and water insoluble raloxifene hydrochloride (Enamine Ltd., Kyiv, Ukraine) and test compounds at a concentration of 20 mM were prepared. To perform the screening, 2.5 µL aliquots of the DMSO stocks of test and reference compounds were added to the wells of a 96-well microplate (Wallac, USA) with clear flat bottom. Then, 250 µL of PBS pH 7.4 (Sigma Aldrich, St. Louis, MO, USA) was added to each well. The final concentration of compounds was 200 µM and the final concentration of DMSO was 1%. After the addition of the buffer, precipitate formation was immediately scanned for each well by measuring the light scattering with a laser nephelometer (Nephelostar, BMG LabTech, Germany). The solubility of each test compound normalized to highly soluble 2′-deoxy-5-fluorouridine and water insoluble raloxifene hydrochloride was calculated. Relative solubility was determined using selected rages: high solubility >0.8, mid solubility: 0.6–0.8 and low solubility <0.6 [42].

#### 3.3.5. Ligand Efficiency

Ligand efficiency was calculated as shown in the following Equation (1) [23]:

(1) $$LE=\frac{1.37}{HA}*{pIC}_{50}$$

where *LE* is ligand efficiency; *HA* is number of heavy atoms; *pIC*_50_ is negative logarithm of *IC*_50_.

## 4. Conclusions

This paper deals with the synthesis and antiplasmodial activities of novel 4-substituted (1,2,5-oxadiazol-3-yl)benzamido derivatives of MMV´s Malaria Box compound **1**. The first series of derivatives focused on the acyl moiety of compounds, whereby the favorable impact of a benzoyl group was confirmed. Substitution of the phenyl ring strongly influenced the antiplasmodial activity as well as the cytotoxicity, providing a number of structure–activity relationships. Activities in sub-micromolar concentrations were observed for all tested 3,4-dialkoxy substituted derivatives and selected 4-nitro analogs. However, all nitrophenyl derivatives showed high cytotoxicity. The most promising new compound **51** had a 4-(3-ethoxy-4-methoxyphenyl) substitution. It showed not only good activity but also low cytotoxicity, resulting in by far the highest selectivity of the new compounds. This strongly indicates the importance of a 3,4-dialkoxy substituted phenyl ring. Whether its 3-methylbenzamido moiety is the ideal substituent in ring position 3 of the furazan should be evaluated in further studies.

## Figures and Tables

**Table 1:** Activities of compounds **37–63** against *P. falciparum* NF54 and L-6 cells, expressed as IC_50_ (µM) ^a^.

Compound	3-Benzamido- Substituent	4-Phenyl- Substituent	*P.f.* NF54 ^b^ IC_50_ (µM)	S.I. = IC_50_ (Cyt.)/IC_50_ (*P.f.*NF54)	Cytotoxicity L-6 Cells IC_50_ (µM)
**37**	H	4-NO_2_	1.815	6.694	12.15
**38**	3-*Me*	4-NO_2_	0.990	3.504	3.469
**39**	3-F	4-NO_2_	5.331	0.809	4.311
**40**	3-CF_3_	4-NO_2_	1.977	0.473	0.936
**53**	3-Cl, 4-O*Me*	4-NO_2_	0.323	10.37	3.349
**41**	H	3-NO_2_	9.105	0.666	6.067
**42**	3-*Me*	3-NO_2_	11.47	0.464	5.319
**43**	3-F	3-NO_2_	26.69	0.104	2.772
**44**	3-CF_3_	3-NO_2_	8.645	0.198	1.713
**54**	3-Cl, 4-O*Me*	3-NO_2_	5.924	0.932	5.524
**49**	3-*Me*	4-F	8.107	4.328	35.09
**45**	3-F	4-F	11.19	1.000	11.19
**50**	3-CF_3_	4-F	8.683	0.852	7.402
**47**	3-*Me*	4-Cl	8.733	15.54	135.7
**46**	3-F	4-Cl	10.09	1.155	11.65
**48**	3-CF_3_	4-Cl	8.444	0.599	5.058
**55**	H	4-NH_2_	7.421	16.62	123.3
**56**	3-*Me*	4-NH_2_	6.834	15.70	107.2
**57**	3-F	4-NH_2_	10.49	18.90	198.1
**58**	3-CF_3_	4-NH_2_	9.260	3.548	32.85
**59**	3-Cl, 4-O*Me*	4-NH_2_	3.136	31.54	98.91
**60**	H	3-NH_2_	53.70	4.039	216.9
**61**	3-*Me*	3-NH_2_	30.17	6.182	186.5
**62**	3-F	3-NH_2_	31.98	6.107	195.3
**63**	3-CF_3_	3-NH_2_	17.69	1.201	21.25
**52**	3-*Me*	4-O*Et*, 3-O*Me*	0.275	86.47	23.78
**51**	3-*Me*	3-O*Et*, 4-O*Me*	0.034	1526	51.87
**1**	3-*Me*	3-O*Et*, 4-O*Et*	0.011	14,483	159.3
**2**	H	3-O*Et*, 4-O*Et*	0.076	1463	111.2
**3**	3-F	3-O*Et*, 4-O*Et*	0.049	839.6	41.09
**4**	3-CF_3_	3-O*Et*, 4-O*Et*	0.019	524.6	9.968
**5**	3-Cl, 4-O*Me*	3-O*Et*, 4-O*Et*	0.014	1431	20.03
**CQ**			0.009	9672	90.92
**POD**					0.012

CQ—chloroquine; POD—podophyllotoxin. ^a^ Values represent the average of four determinations (two determinations of two independent experiments); ^b^ sensitive to chloroquine.

**Table 2:** Key physicochemical parameters, CYP3A4 inhibition and aqueous solubility of compounds **37–63**.

Compound	log P ^a^	LE (kcal/mol/HA)	CYP3A4 Inhibition ^b^ (%)	Relative Solubility ^c^
**37**	3.22	0.342	46	0.73
**38**	3.73	0.343	68	−0.60
**39**	3.36	0.301	43	0.05
**40**	4.10	0.289	82	−0.43
**53**	3.67	0.342		0.05
**41**	3.22	0.300	82	0.98
**42**	3.73	0.282	87	−0.53
**43**	3.36	0.261	79	0.84
**44**	4.10	0.257	94	−0.49
**54**	3.67	0.275		−0.81
**45**	3.57	0.308	60	0.96
**46**	4.03	0.311		0.32
**47**	4.40	0.315	57	−0.47
**48**	4.47	0.278		−1.64
**49**	3.94	0.317	84	0.54
**50**	4.30	0.277	86	−0.46
**51**	3.84	0.394	66	−0.42
**52**	3.84	0.346	52	−0.38
**55**	2.45	0.335		0.96
**56**	2.97	0.322		0.98
**57**	2.60	0.310		0.55
**58**	3.33	0.276		−0.04
**59**	2.90	0.314		0.49
**60**	2.45	0.279		1.00
**61**	2.97	0.281	99	0.98
**62**	2.60	0.280		1.00
**63**	3.33	0.260		1.00
**1**	4.19	0.404	45	

^a^ log P was calculated using the ChemAxon software JChem for Excel 14.9.1500.912 (2014) (Chemaxon, Budapest, Hungary); ^b^ determined by Cytochrom P450 3A4 inhibition assay; ^c^ values represent solubility of each compound in PBS pH 7.4 with 1% of DMSO at room temperature normalized to 2′-deoxy-5-fluorouridine (100% soluble) and raloxifene hydrochloride (very low solubility).

## Data Availability

The data presented in this study are available in this article.

## Supplementary Materials

The supporting information can be downloaded at: https://www.mdpi.com/article/10.3390/ijms241914480/s1.
