# Supplementary material for: Structure–Activity Relationships and Antiplasmodial Potencies of Novel 3,4-Disubstituted 1,2,5-Oxadiazoles
Source: Int J Mol Sci. 2023 Sep 23;24(19):14480. doi: 10.3390/ijms241914480 (PMC10572347; doi:10.3390/ijms241914480)

Supplementary Information

Structure–Activity Relationships and Antiplasmodial Potencies of Novel 3,4-Disubstituted 1,2,5-Oxadiazoles

Patrick Hochegger [1], Theresa Hermann [1,*], Johanna Dolensky [1], Werner Seebacher [1], Robert Saf [2], Eva-Maria Pferschy-Wenzig [3], Marcel Kaiser [4,5], Pascal Mäser [4,5] and Robert Weis [1]

[1] Institute of Pharmaceutical Sciences, Pharmaceutical Chemistry, University of Graz, Schubertstraße 1, A-8010 Graz, Austria; patrick.hochegger@yahoo.de (P.H.); johanna.faist@uni-graz.at (J.D.); we.seebacher@uni-graz.at (W.S.); robert.weis@uni-graz.at (R.W.)

[2] Institute for Chemistry and Technology of Materials (ICTM), Graz University of Technology, Stremayrgasse 9, A-8010 Graz, Austria; robert.saf@tugraz.at

[3] Institute of Pharmaceutical Sciences, Pharmacognosy, University of Graz, Beethovenstraße 8, A-8010 Graz, Austria; eva-maria.wenzig@uni-graz.at

[4] Swiss Tropical and Public Health Institute, Kreuzstraße 2, CH-4123 Allschwil, Switzerland; marcel.kaiser@swisstph.ch (M.K.); pascal.maeser@swisstph.ch (P.M.)

[5] Faculty of Philosophy and Natural Sciences, University of Basel, Swiss TPH, Petersplatz 1, CH-4003 Basel, Switzerland

* Correspondence: theresa.hermann@uni-graz.at; Tel.: +43-316-380-5381

NMR spectra data of compounds 25-27, 29-47 and 49-63

Figure S1. ^{1}H NMR at 400 MHz and ^{13}C NMR at 100 MHz spectra for compound **25**.

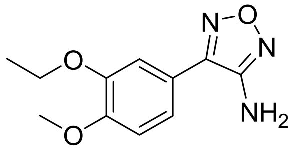

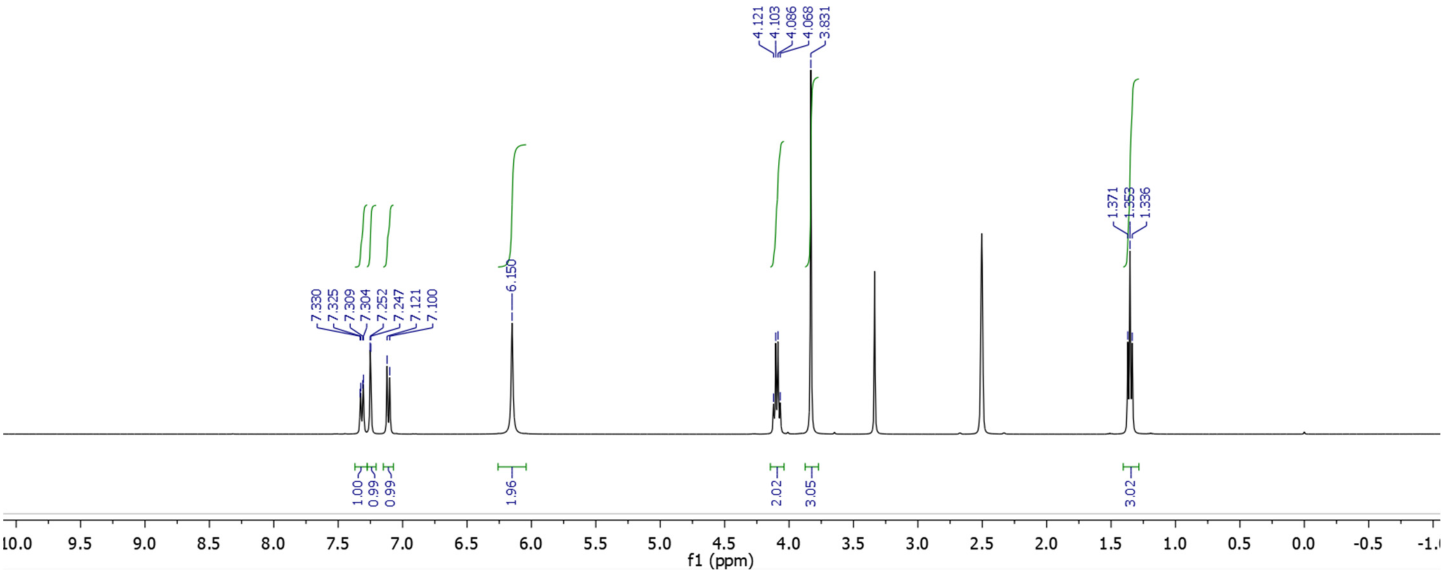

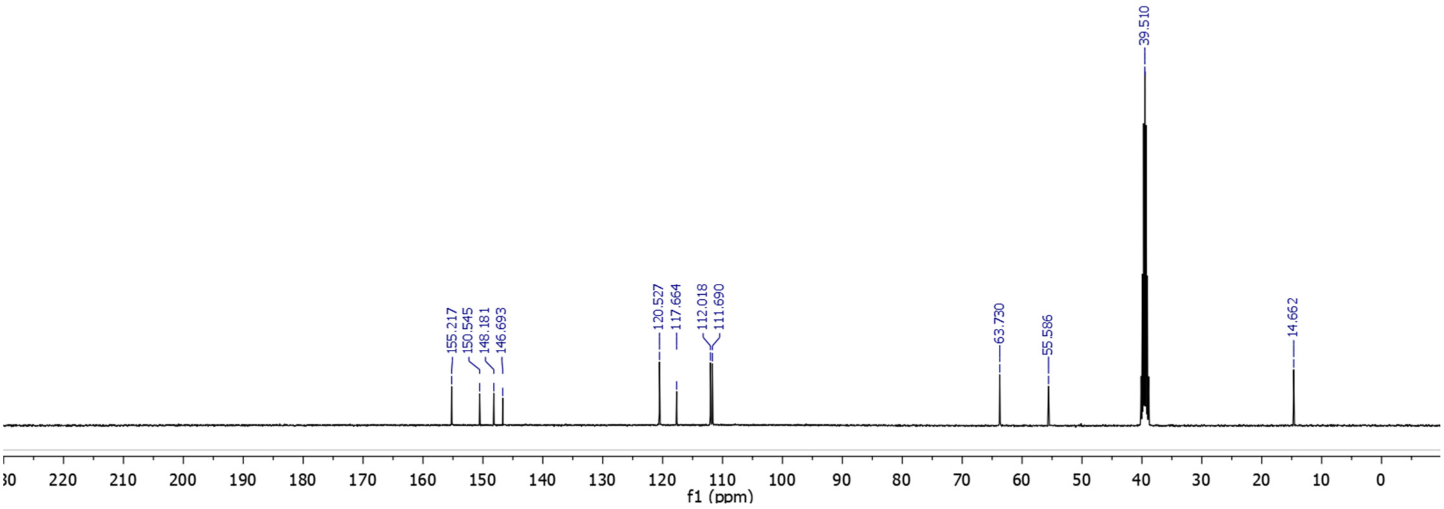

Figure S2. ^{1}H NMR at 400 MHz and ^{13}C NMR at 100 MHz spectra for compound **26**.

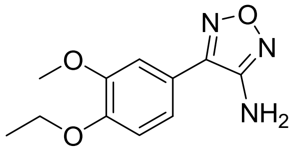

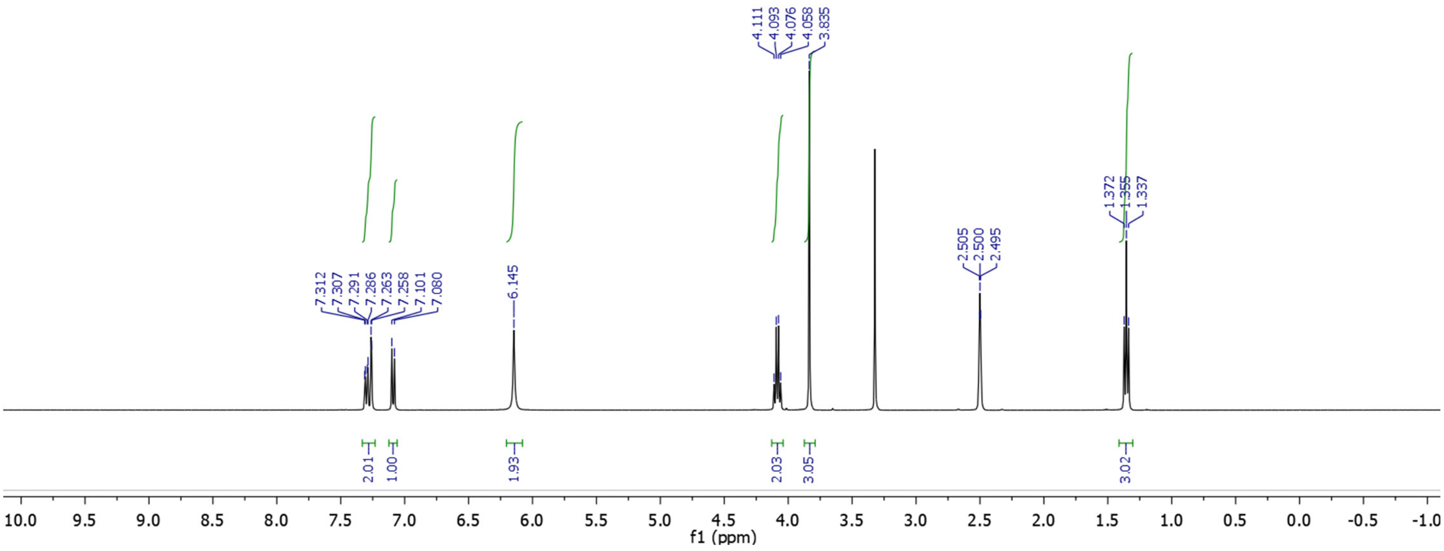

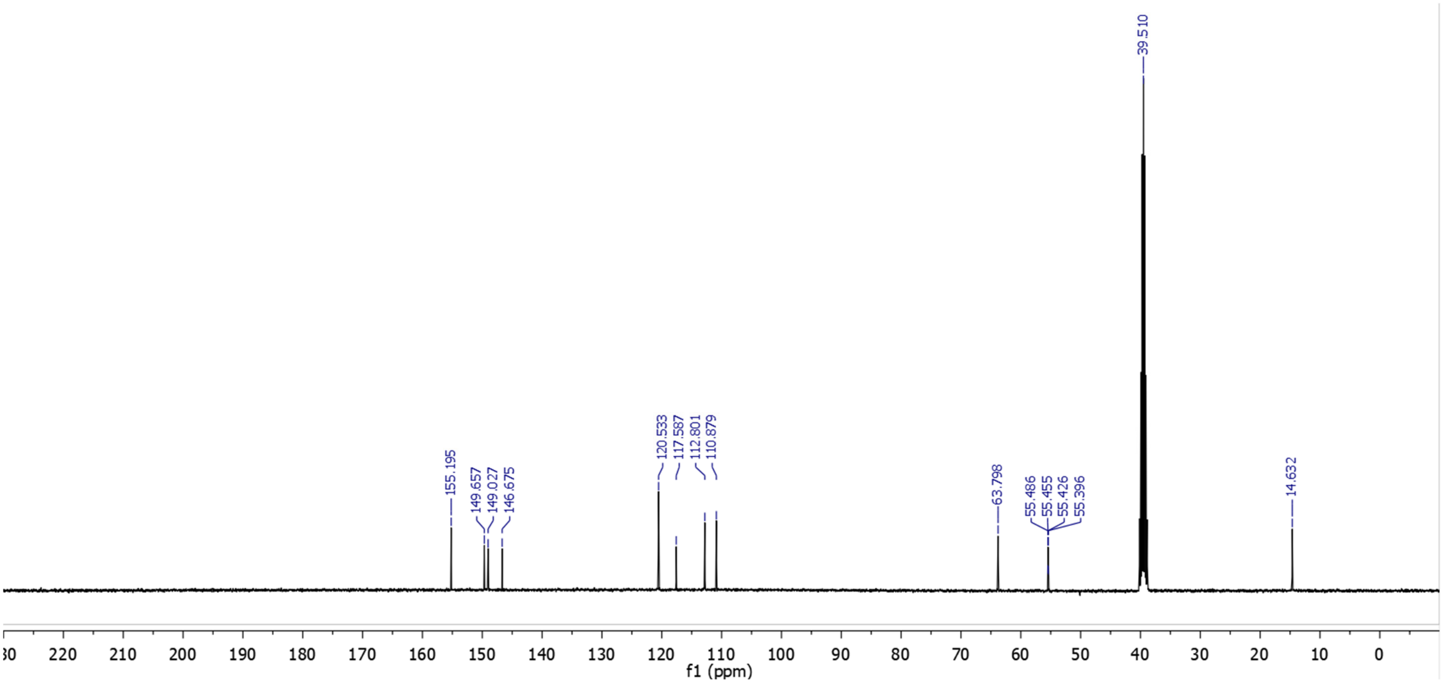

Figure S3. ^{1}H NMR at 400 MHz and ^{13}C NMR at 100 MHz spectra for compound **27**.

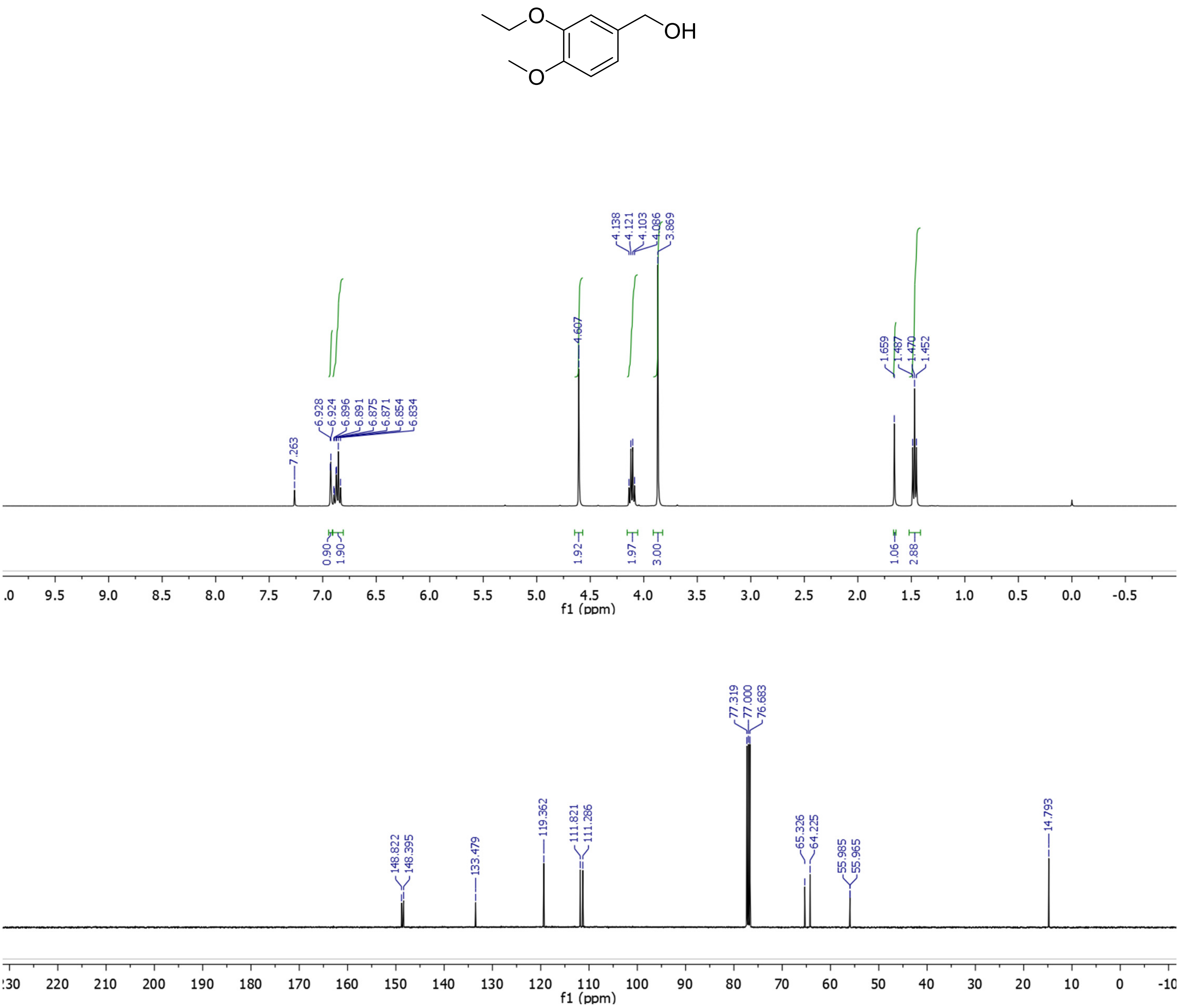

Figure S4. ^{1}H NMR at 400 MHz and ^{13}C NMR at 100 MHz spectra for compound **29**.

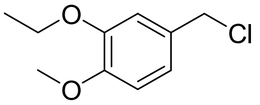

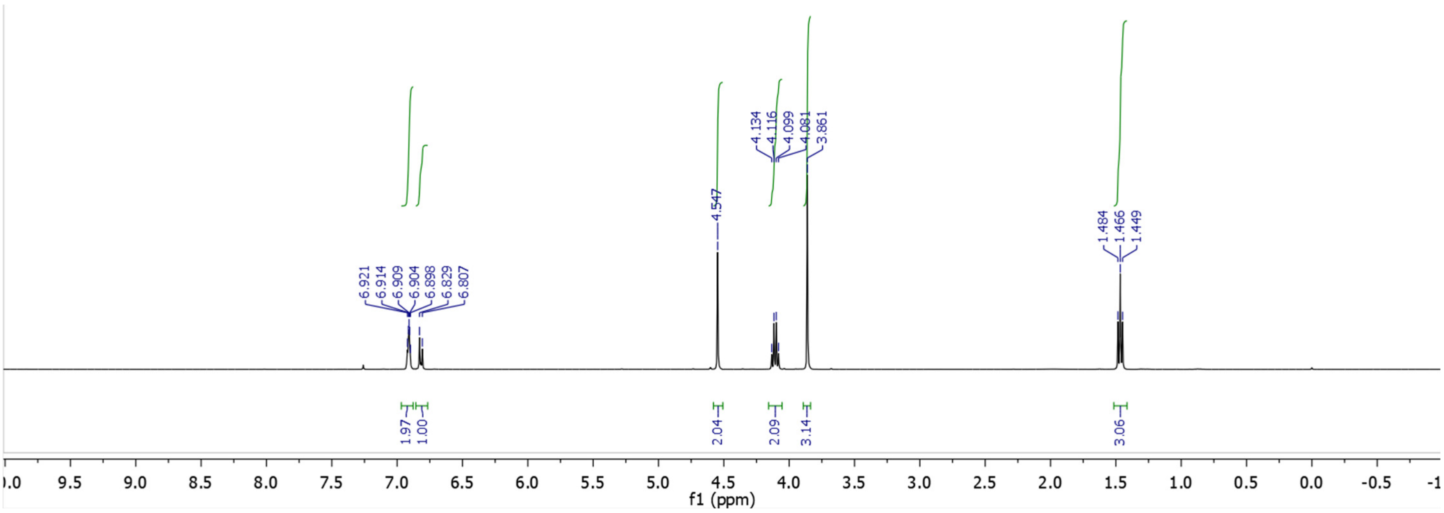

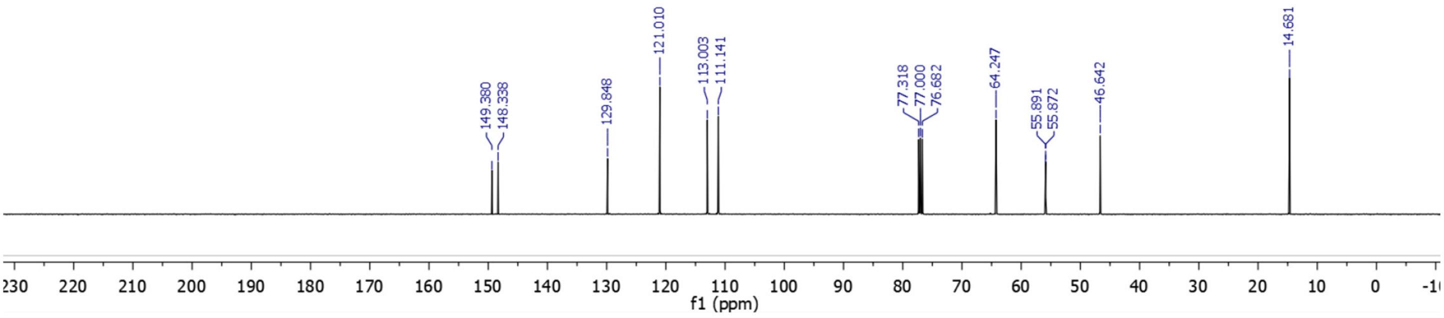

Figure S5. ^{1}H NMR at 400 MHz and ^{13}C NMR at 100 MHz spectra for compound **30**.

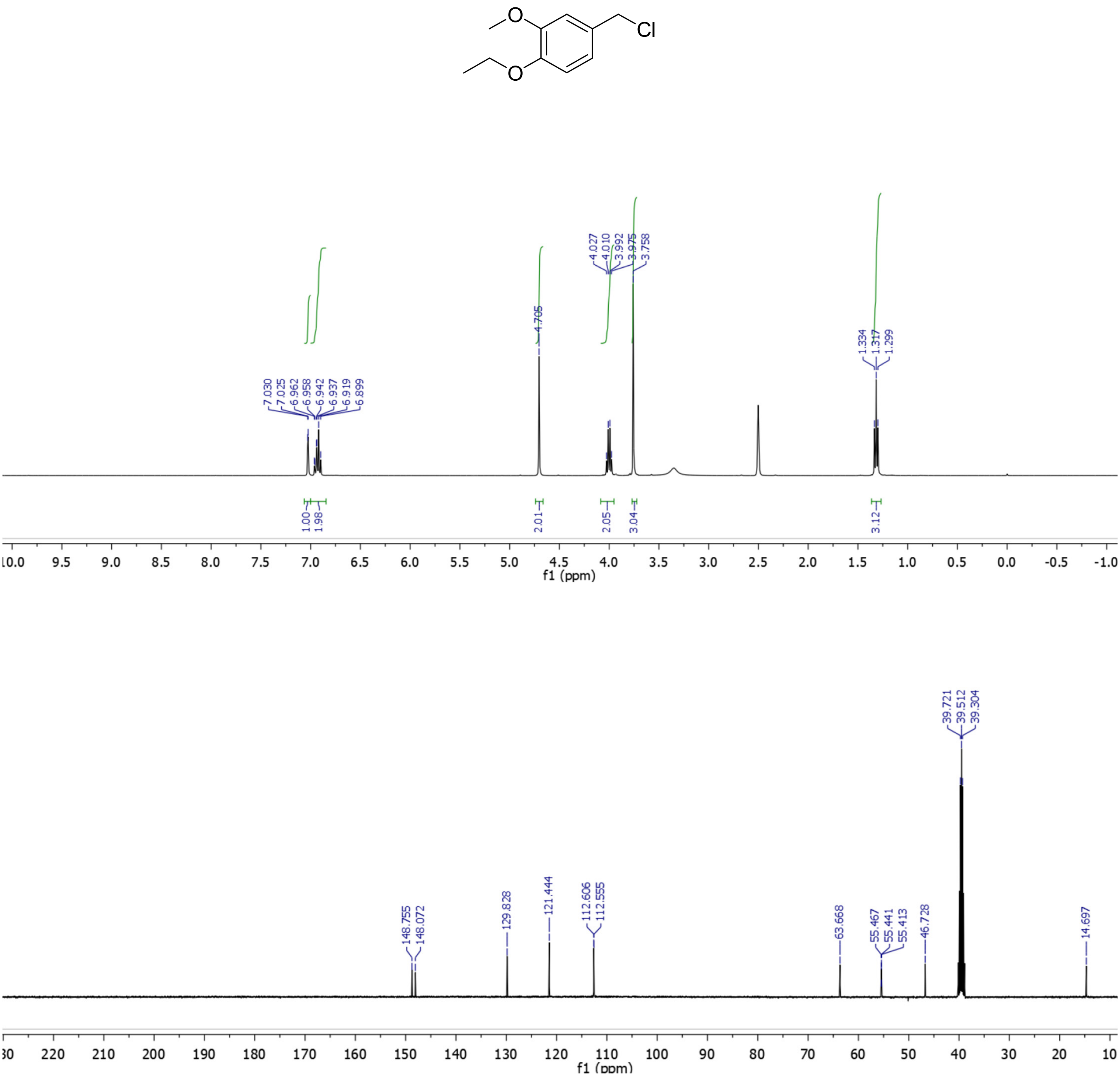

Figure S6. ^{1}H NMR at 400 MHz and ^{13}C NMR at 100 MHz spectra for compound **31**.

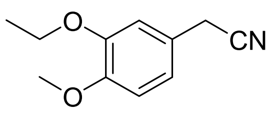

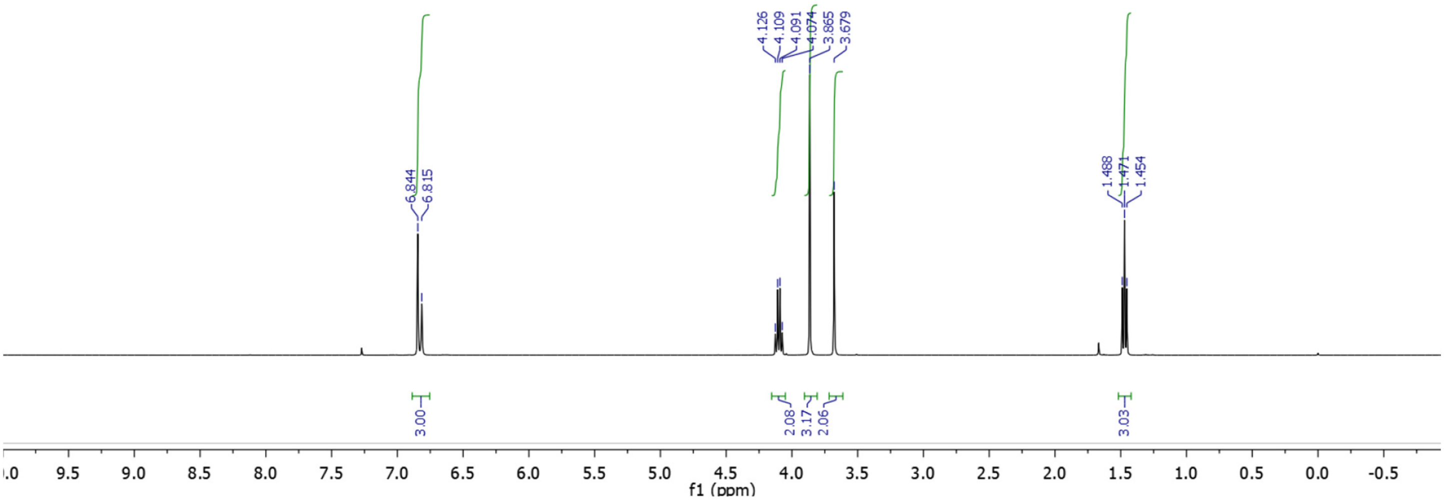

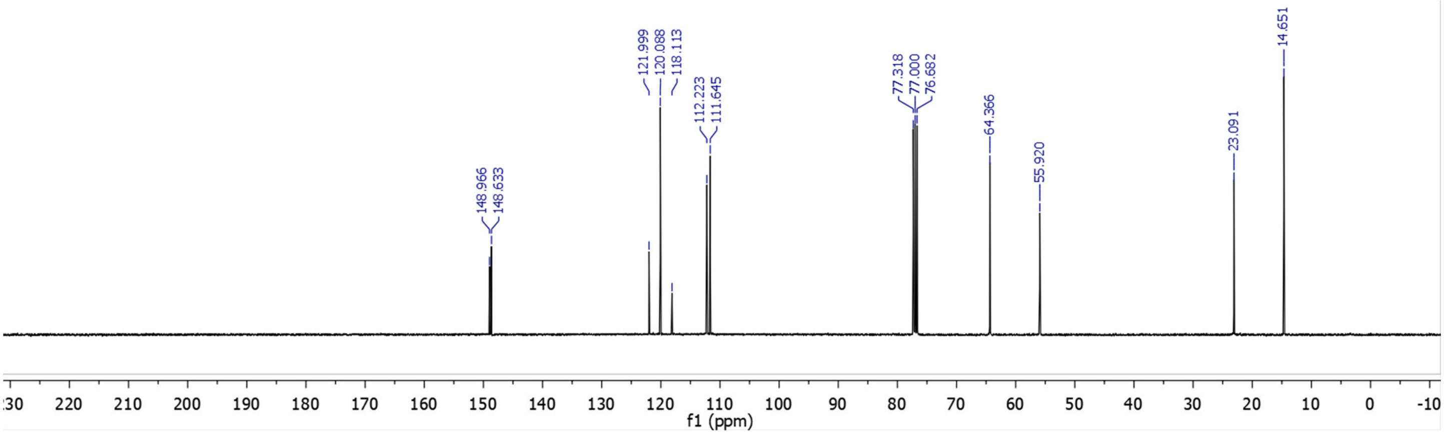

Figure S7. ^{1}H NMR at 400 MHz and ^{13}C NMR at 100 MHz spectra for compound **32**.

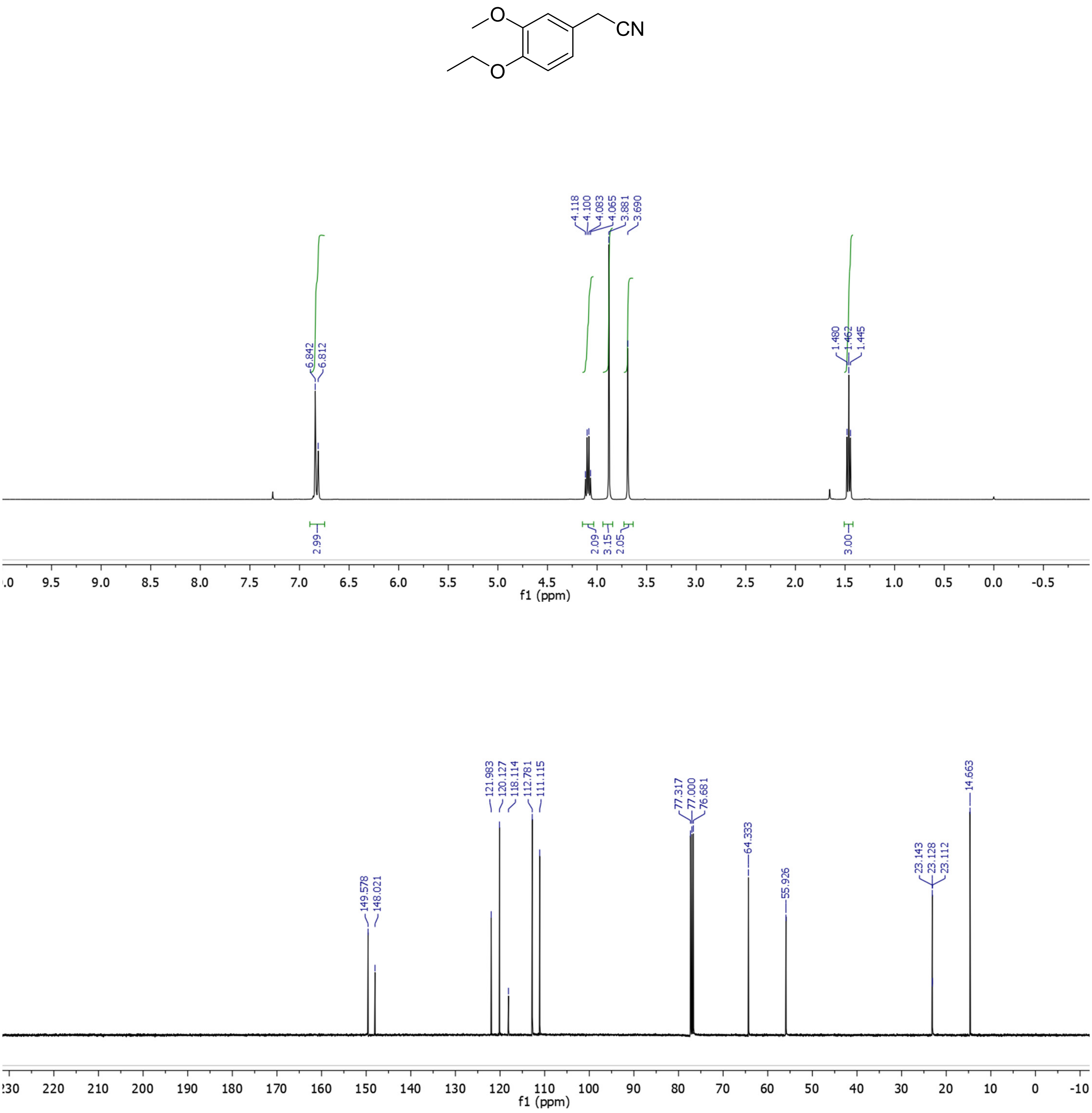

Figure S8. ^{1}H NMR at 400 MHz and ^{13}C NMR at 100 MHz spectra for compound **33**.

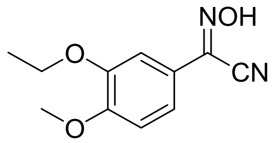

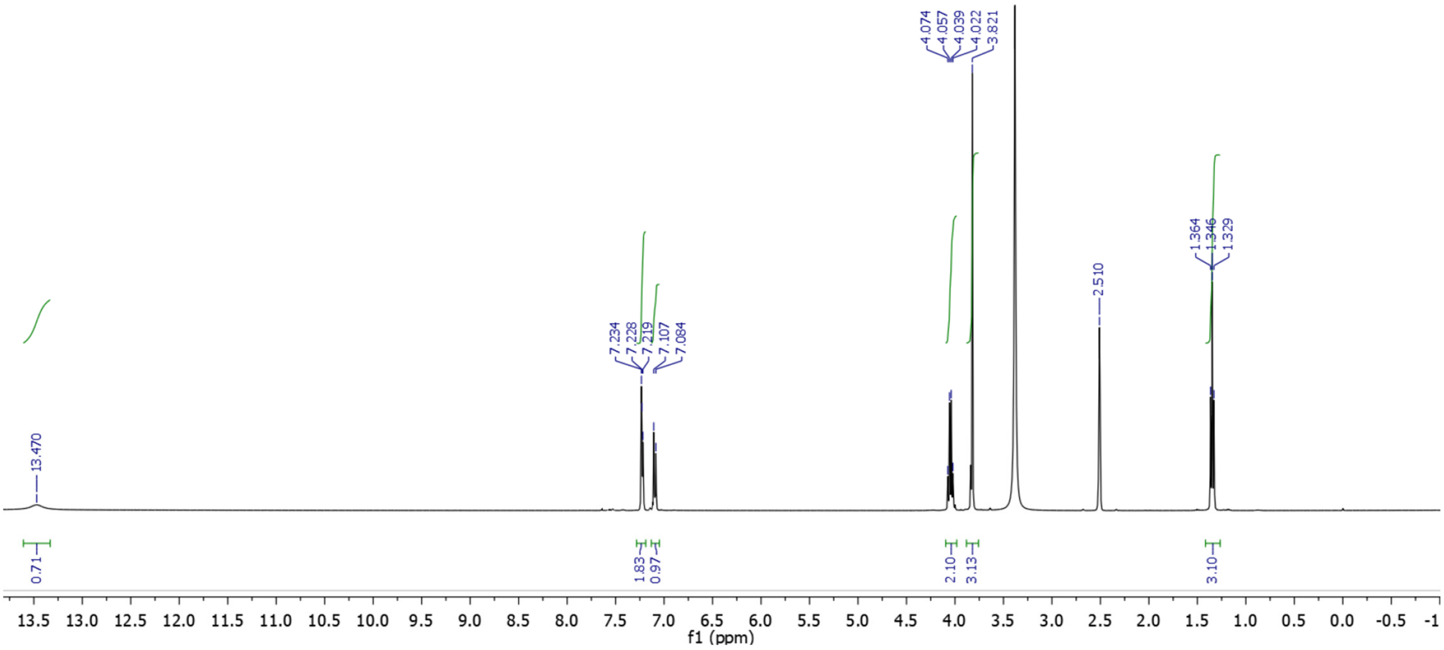

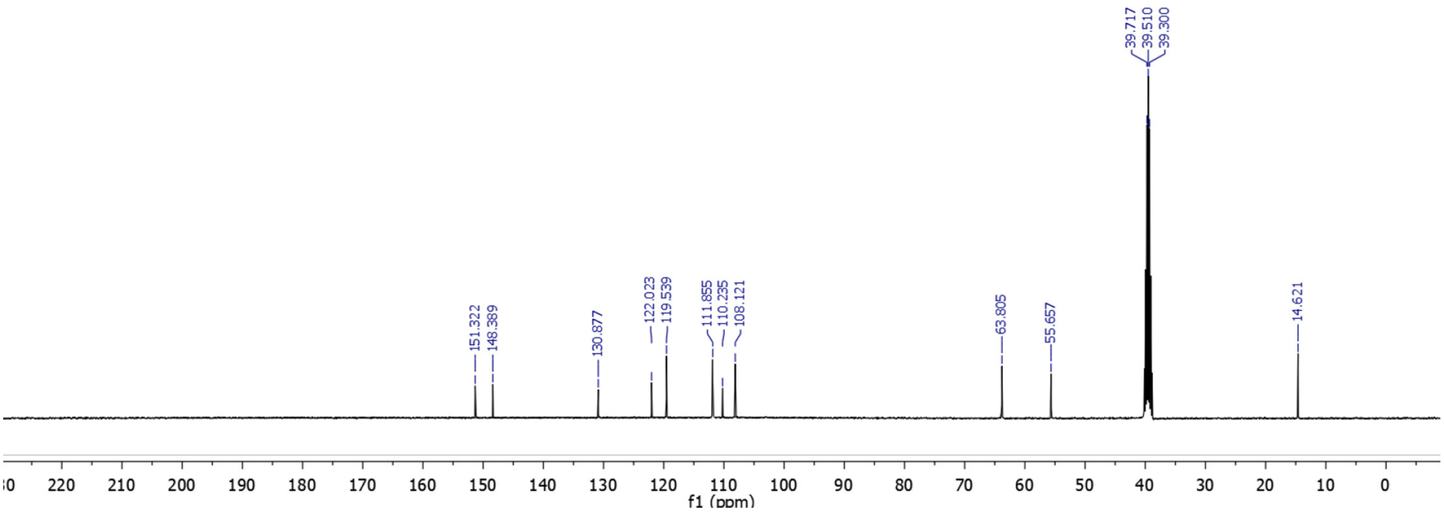

Figure S9. ^{1}H NMR at 400 MHz and ^{13}C NMR at 100 MHz spectra for compound **34**.

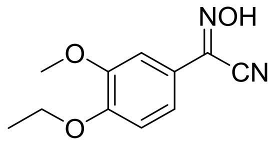

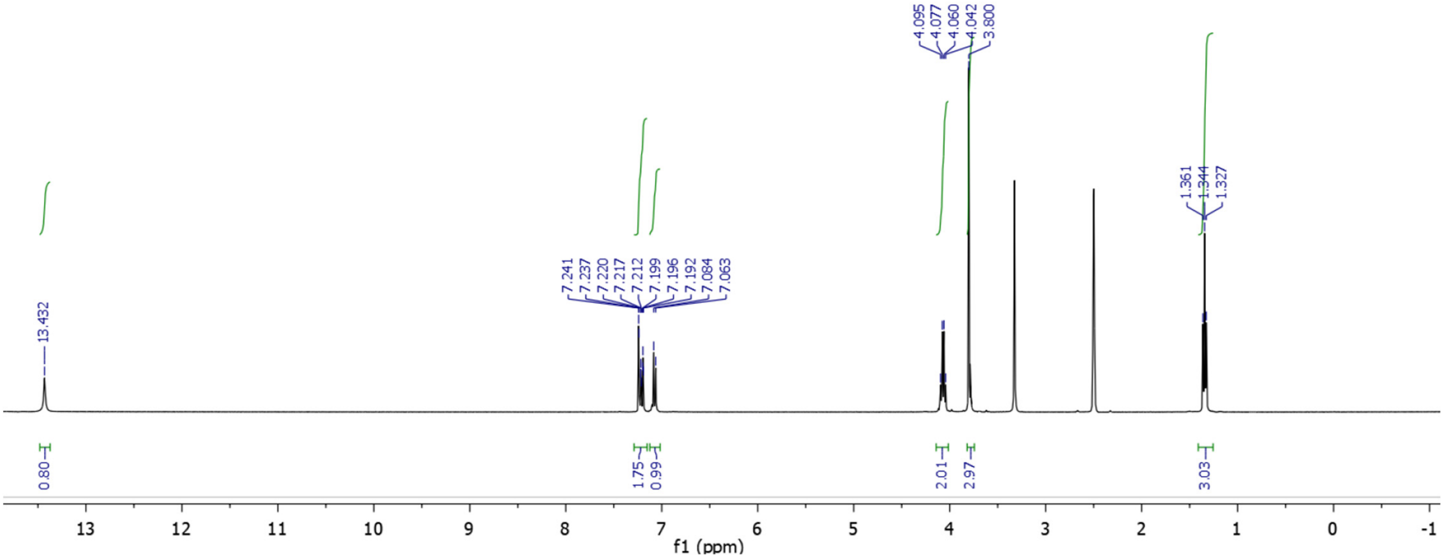

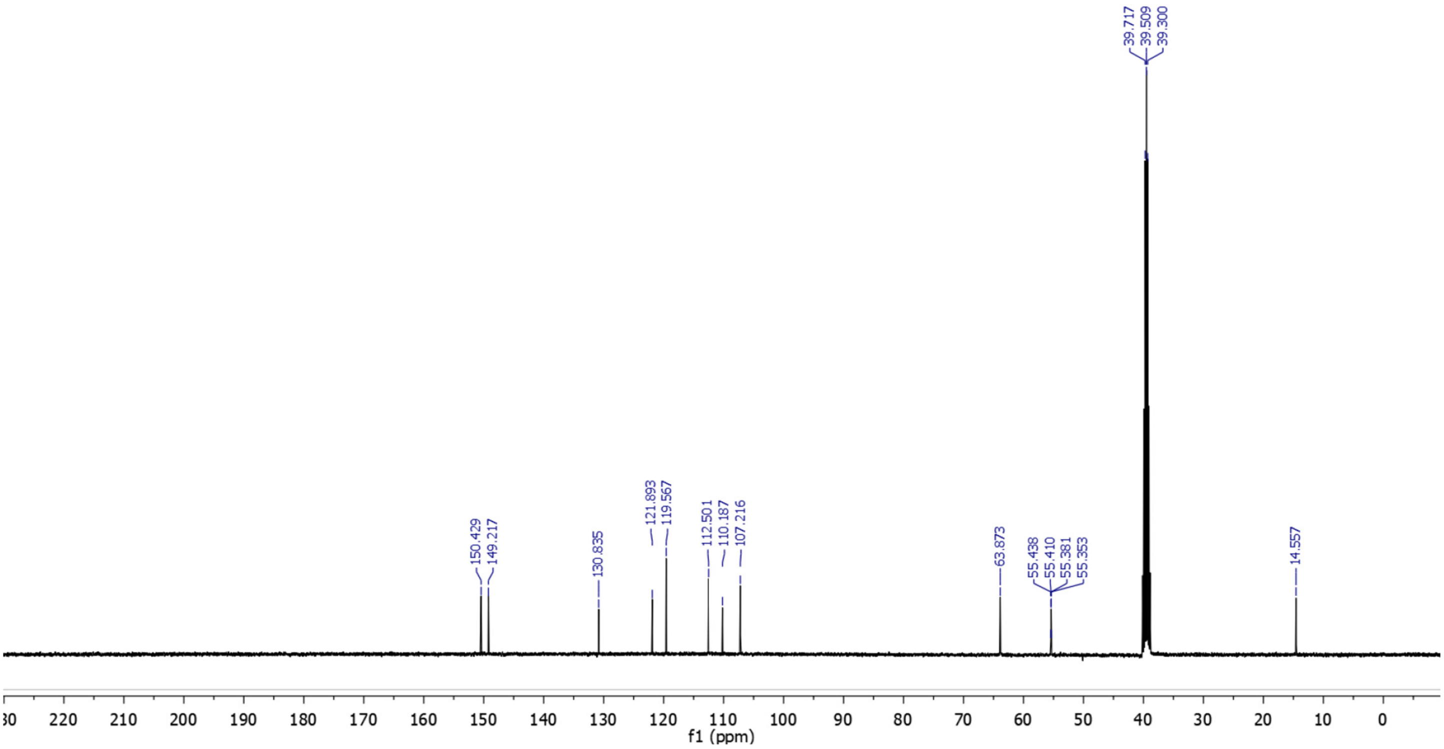

Figure S10. ^{1}H NMR at 400 MHz and ^{13}C NMR at 100 MHz spectra for compound **35**.

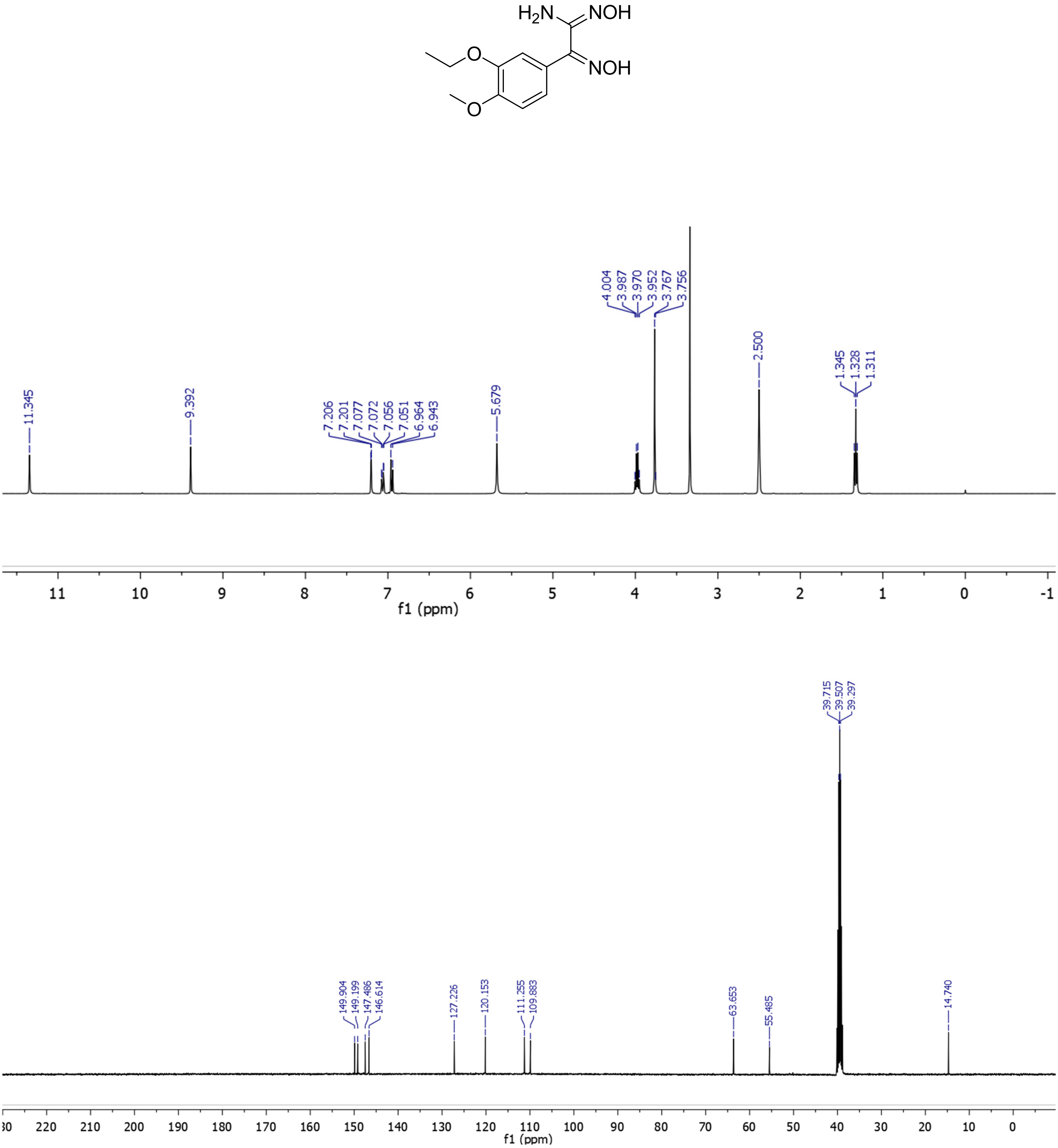

Figure S11. ^{1}H NMR at 400 MHz and ^{13}C NMR at 100 MHz spectra for compound **36**.

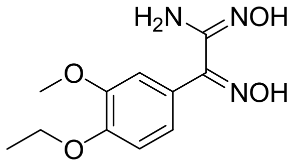

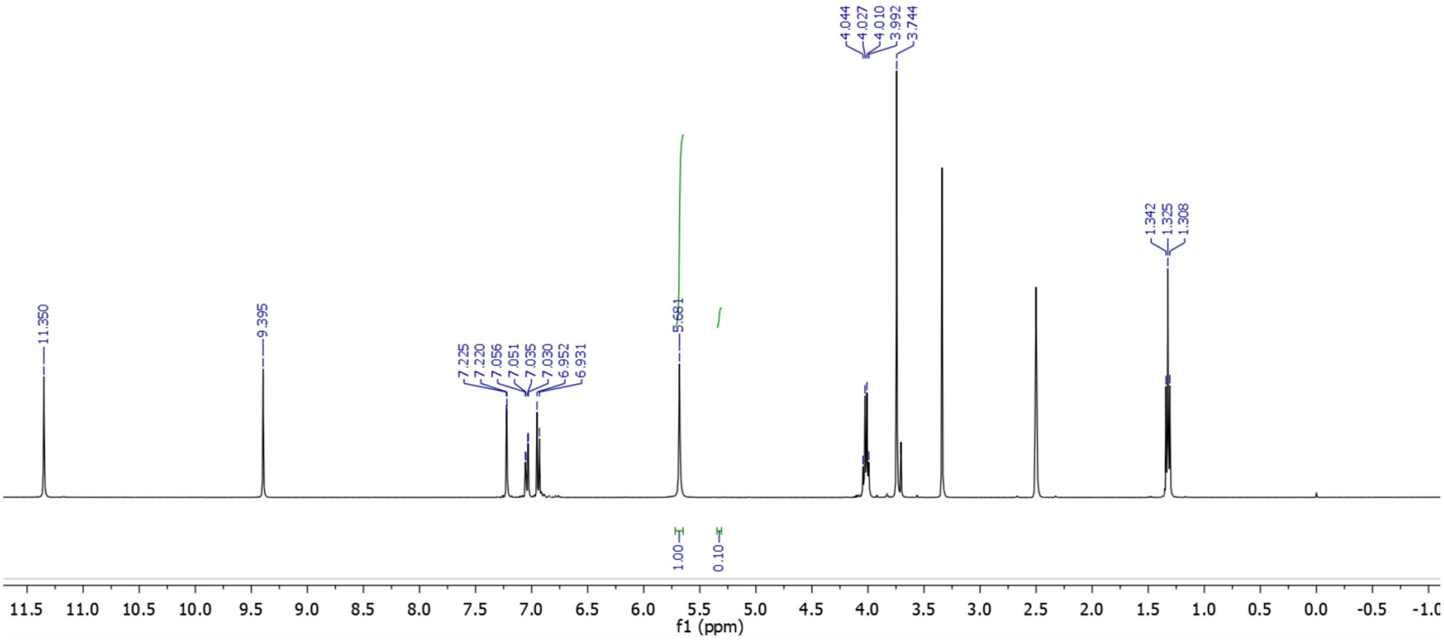

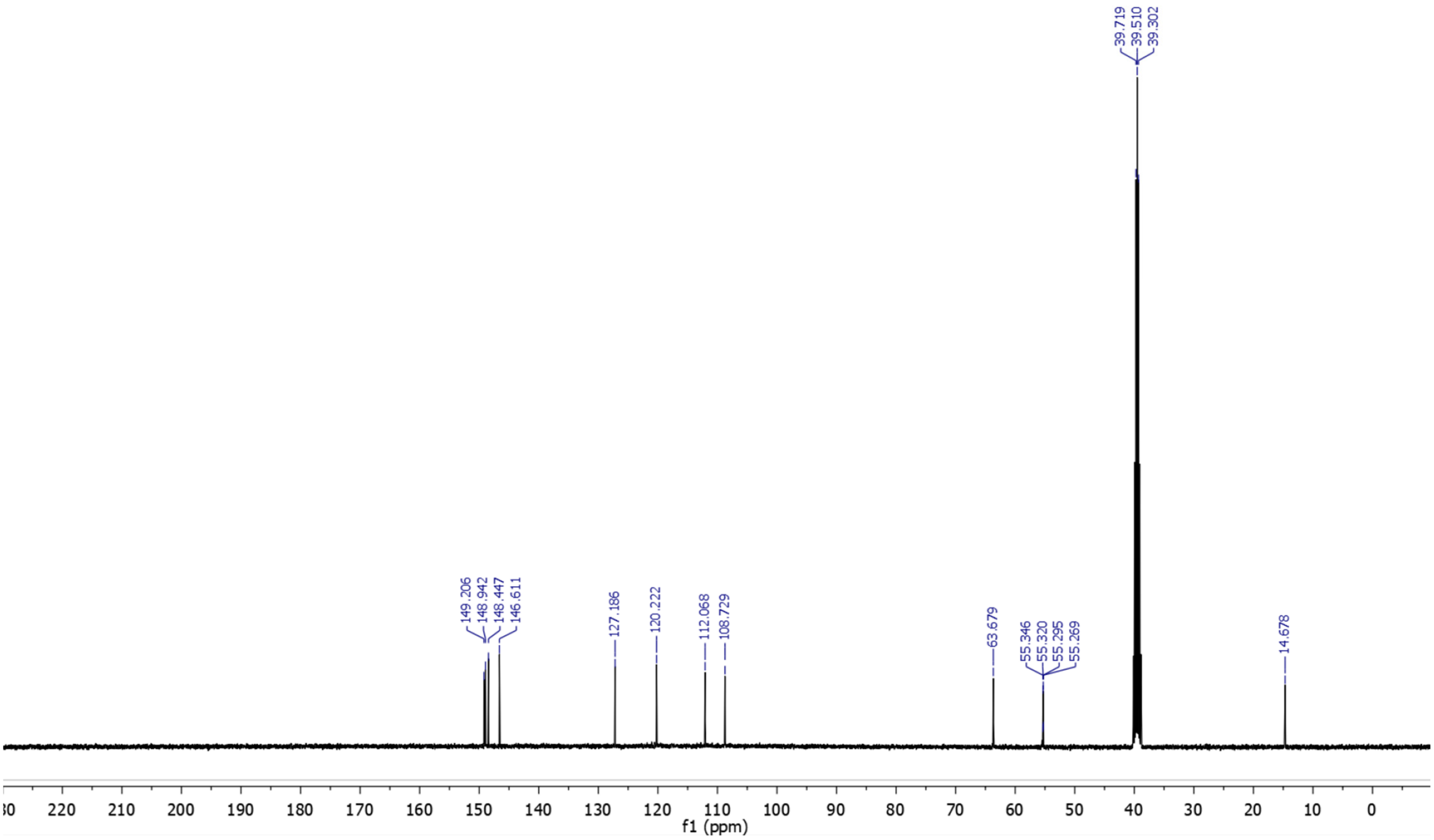

Figure S12. ^{1}H NMR at 400 MHz and ^{13}C NMR at 100 MHz spectra for compound **37**.

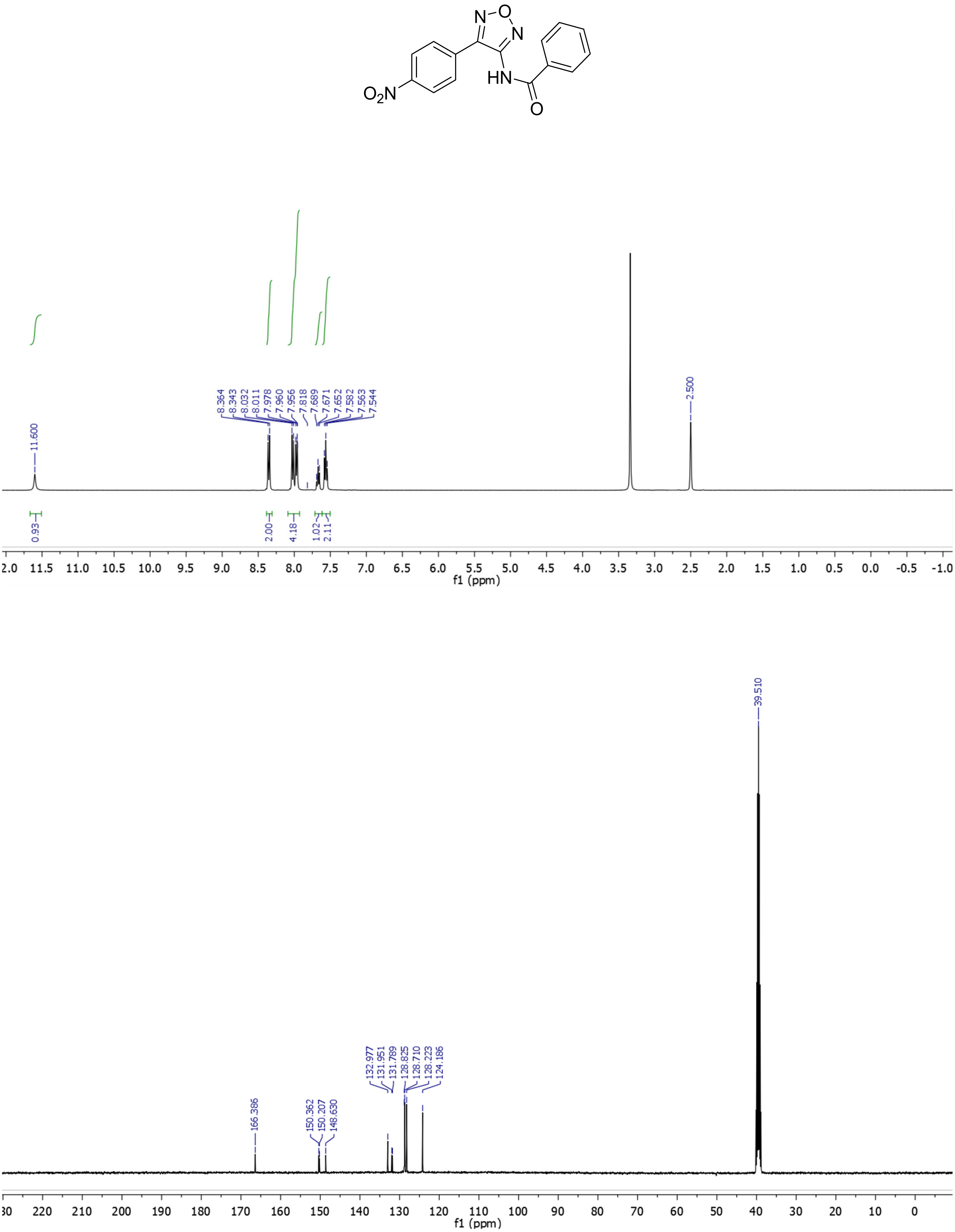

Figure S13. ^{1}H NMR at 400 MHz and ^{13}C NMR at 100 MHz spectra for compound **38**.

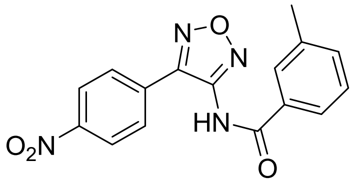

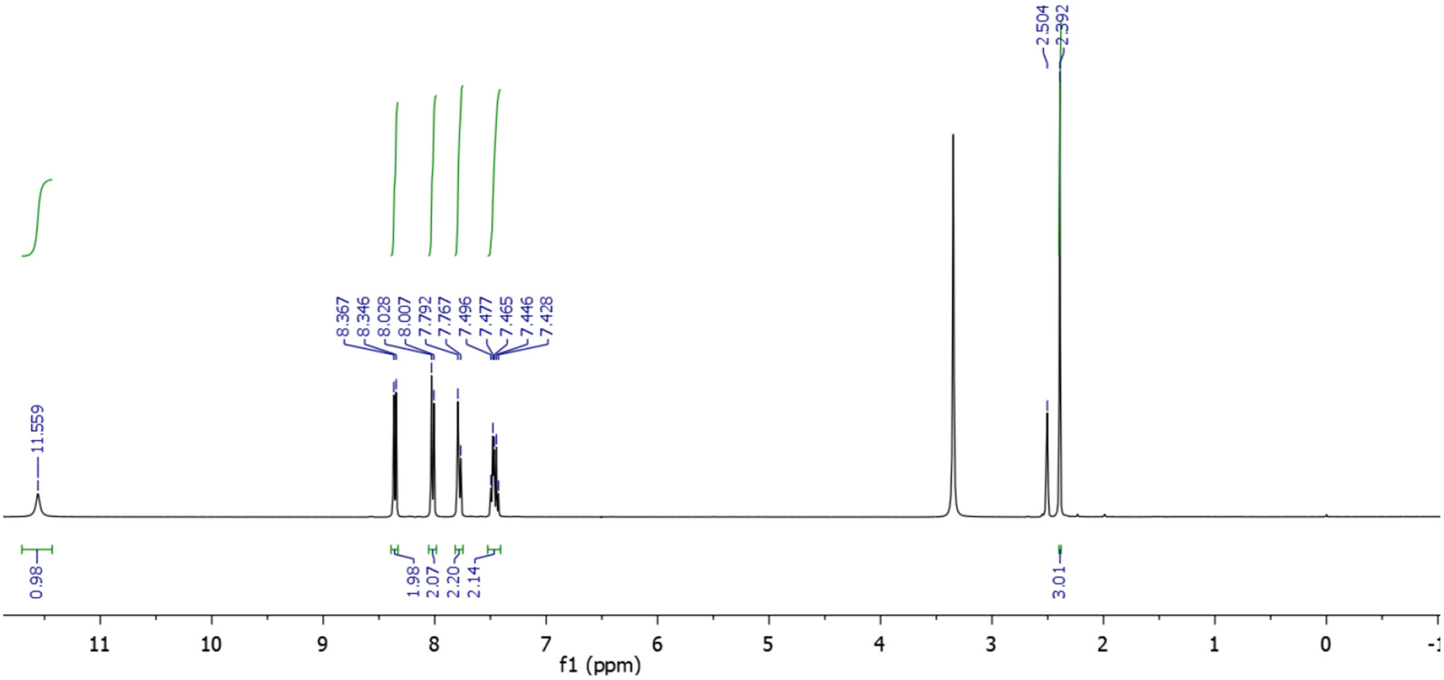

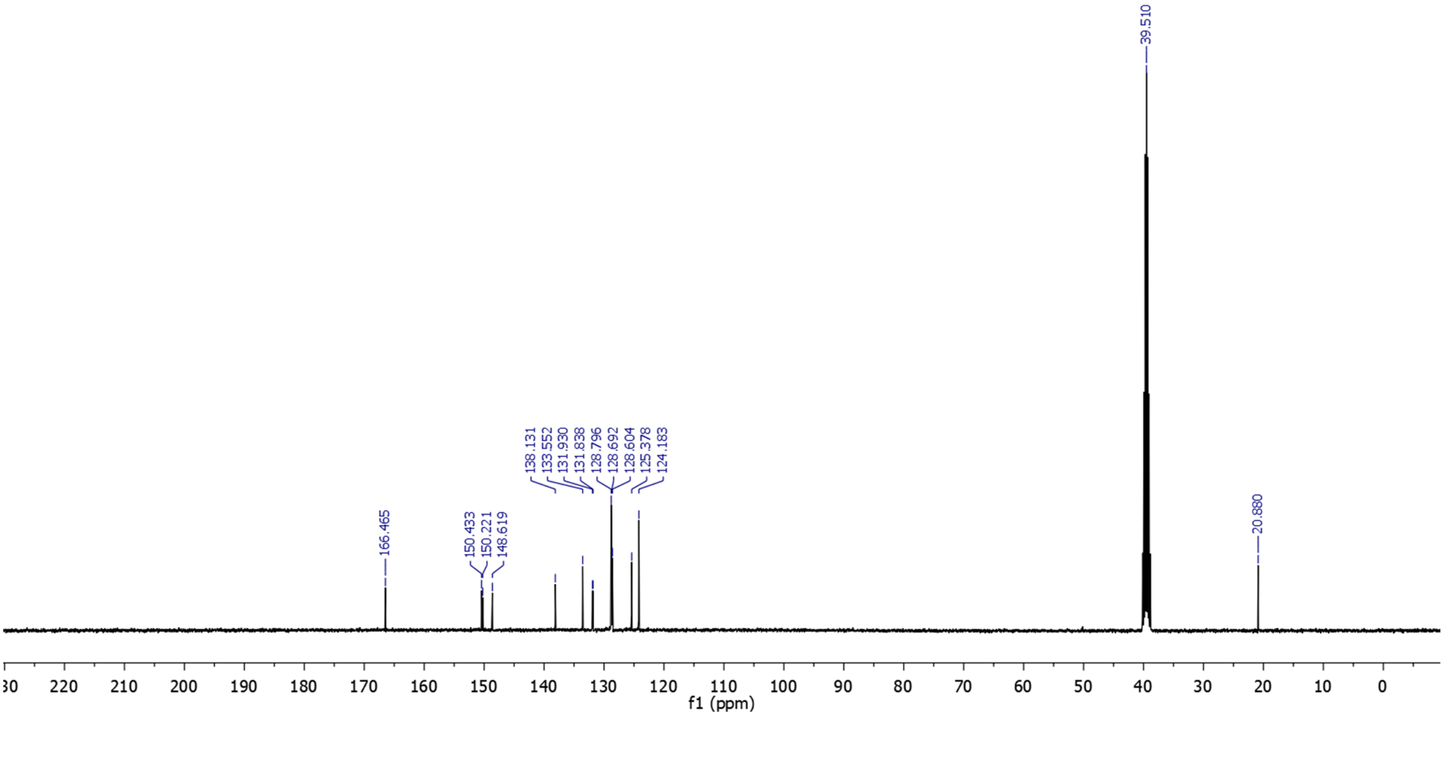

Figure S14. ^{1}H NMR at 400 MHz and ^{13}C NMR at 100 MHz spectra for compound **39**.

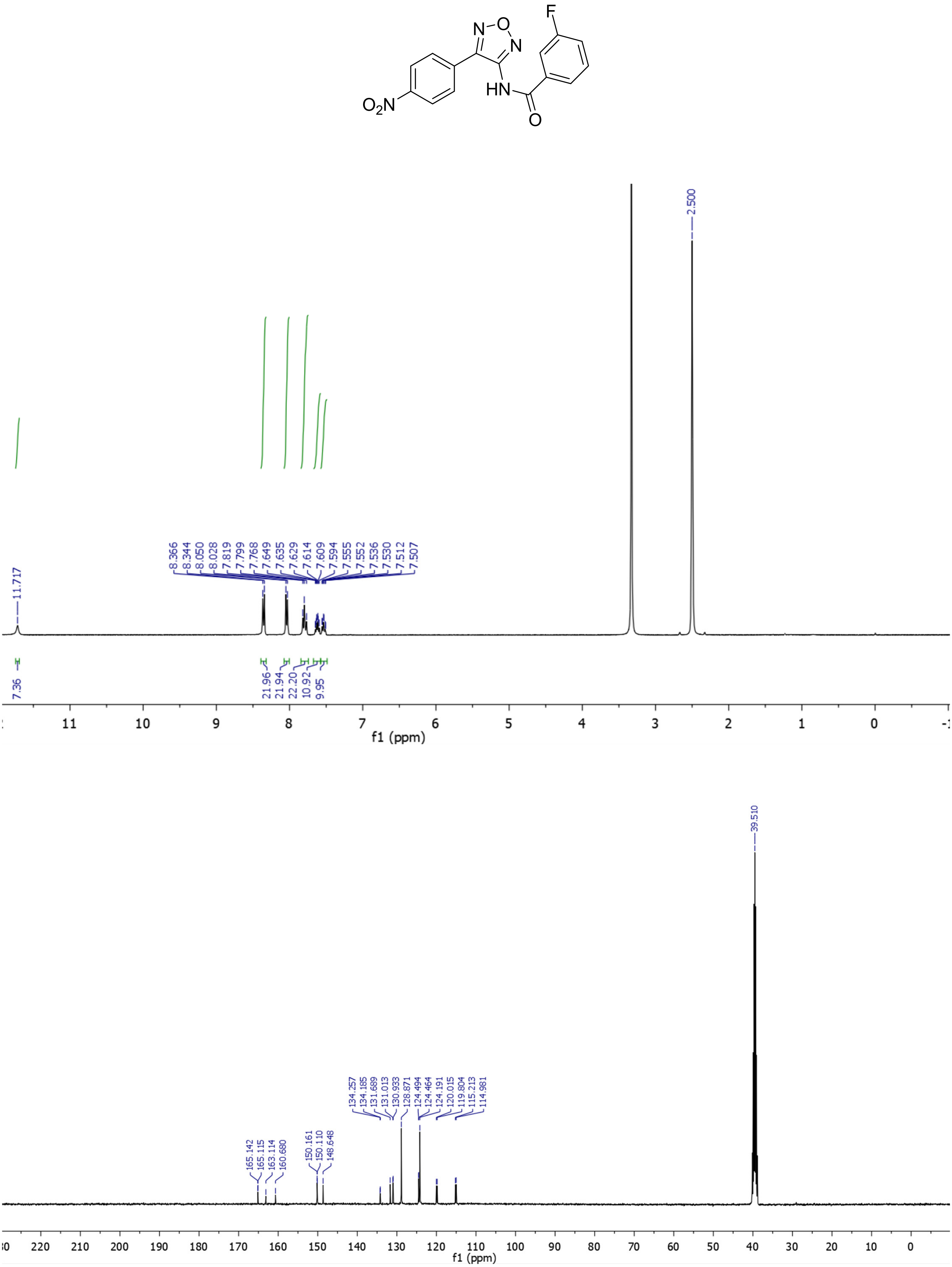

Figure S15. ^{1}H NMR at 400 MHz and ^{13}C NMR at 100 MHz spectra for compound **40**.

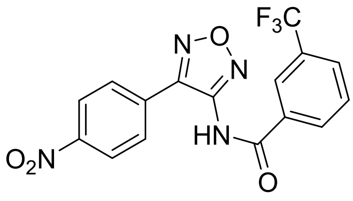

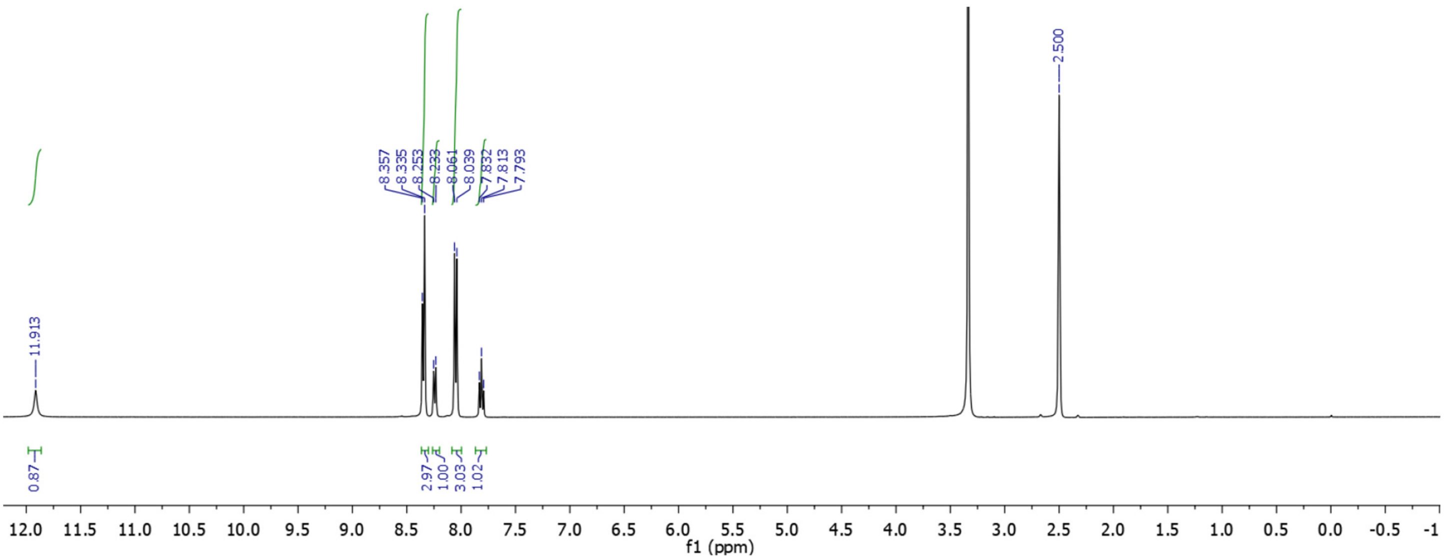

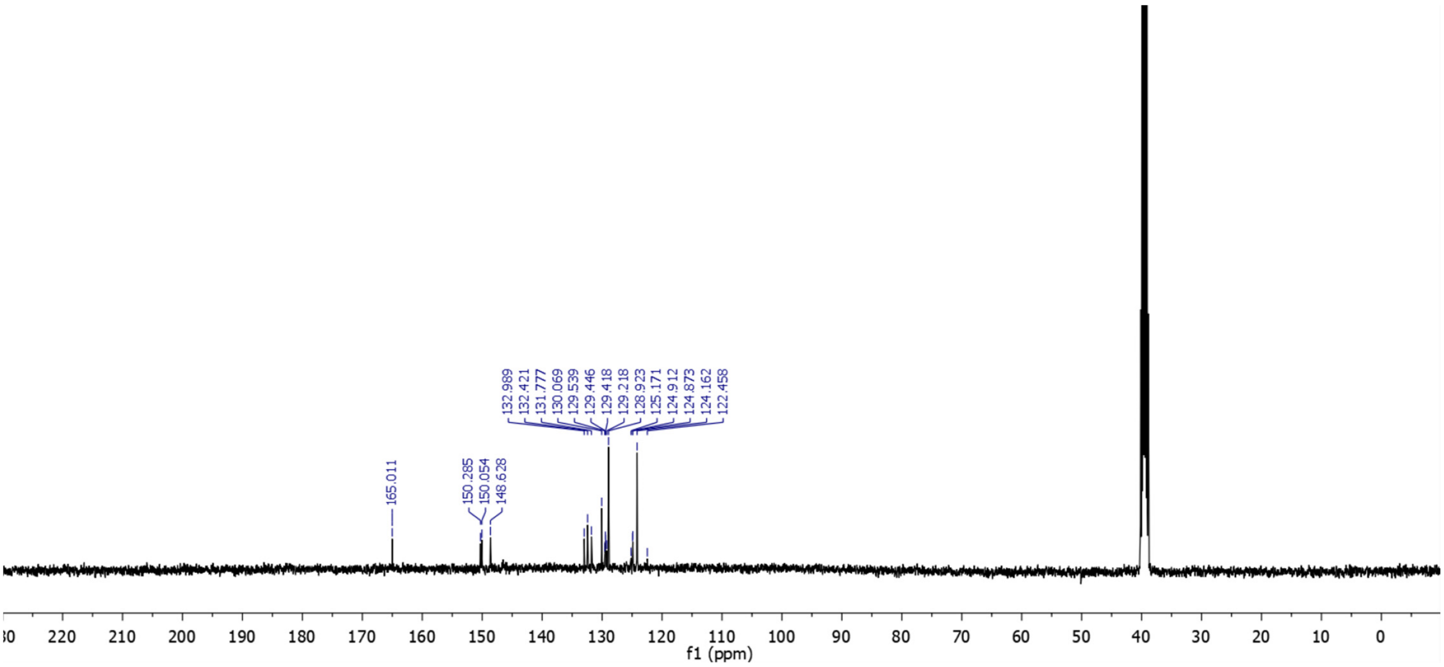

Figure S16. ^{1}H NMR at 400 MHz and ^{13}C NMR at 100 MHz spectra for compound **41**.

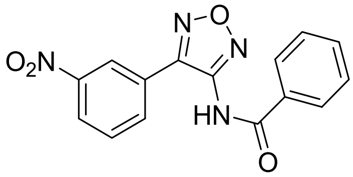

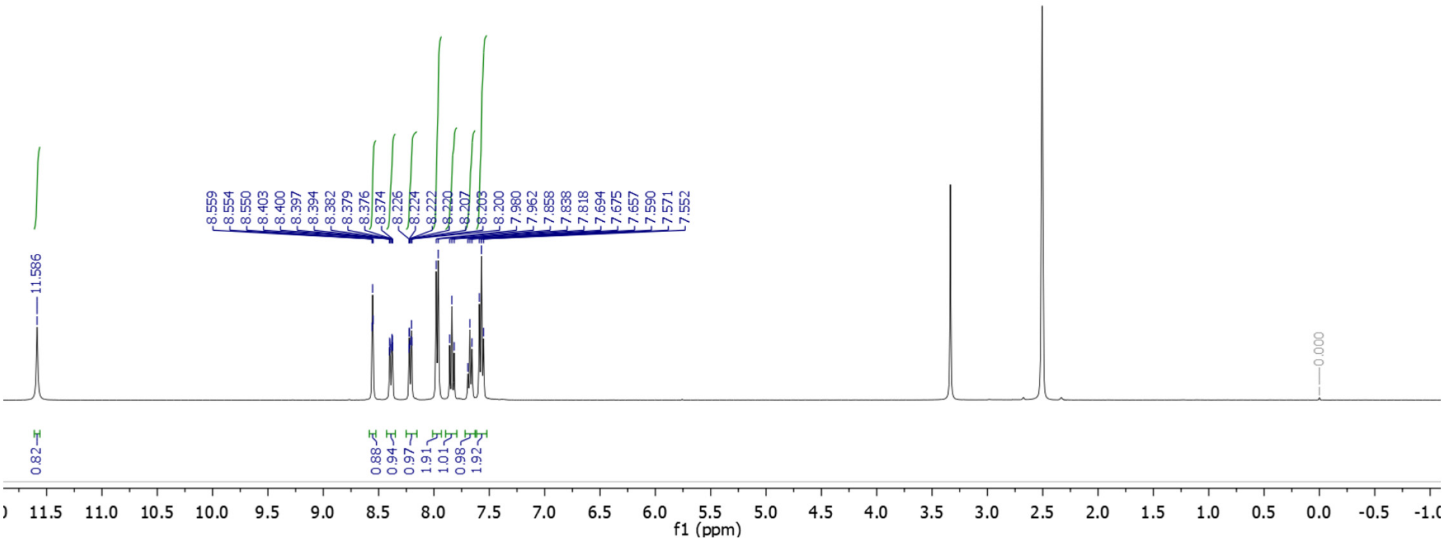

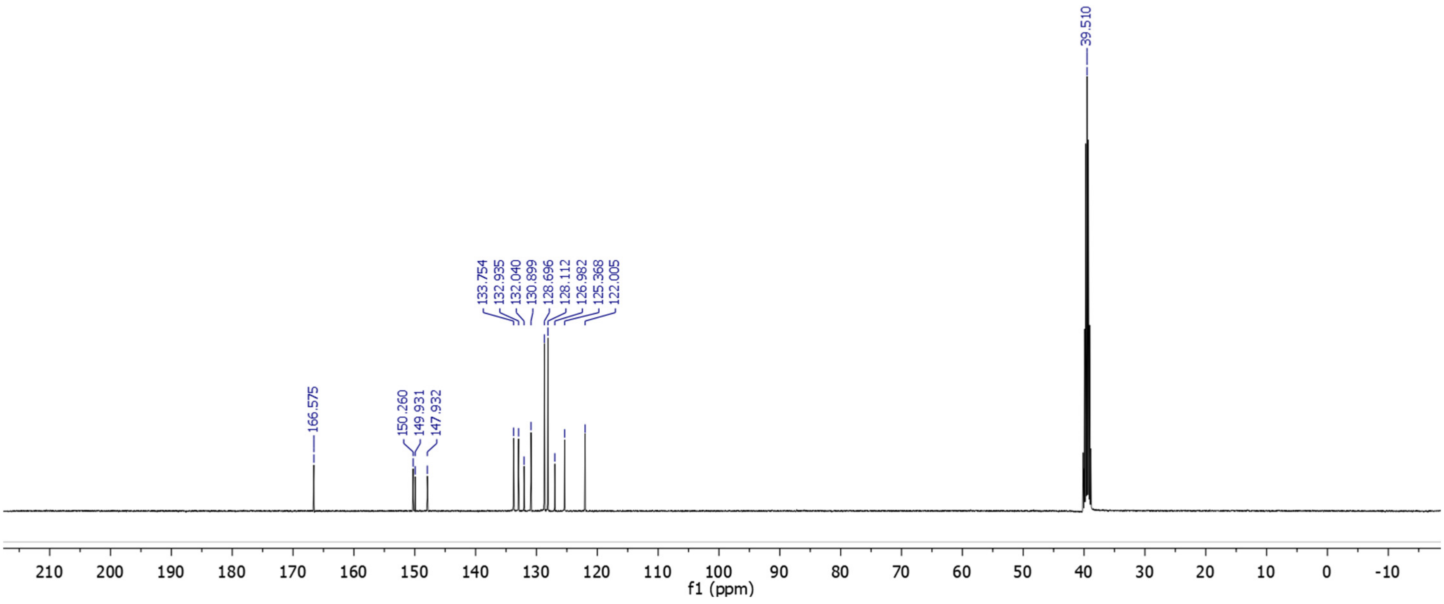

Figure S17. ^{1}H NMR at 400 MHz and ^{13}C NMR at 100 MHz spectra for compound **42**.

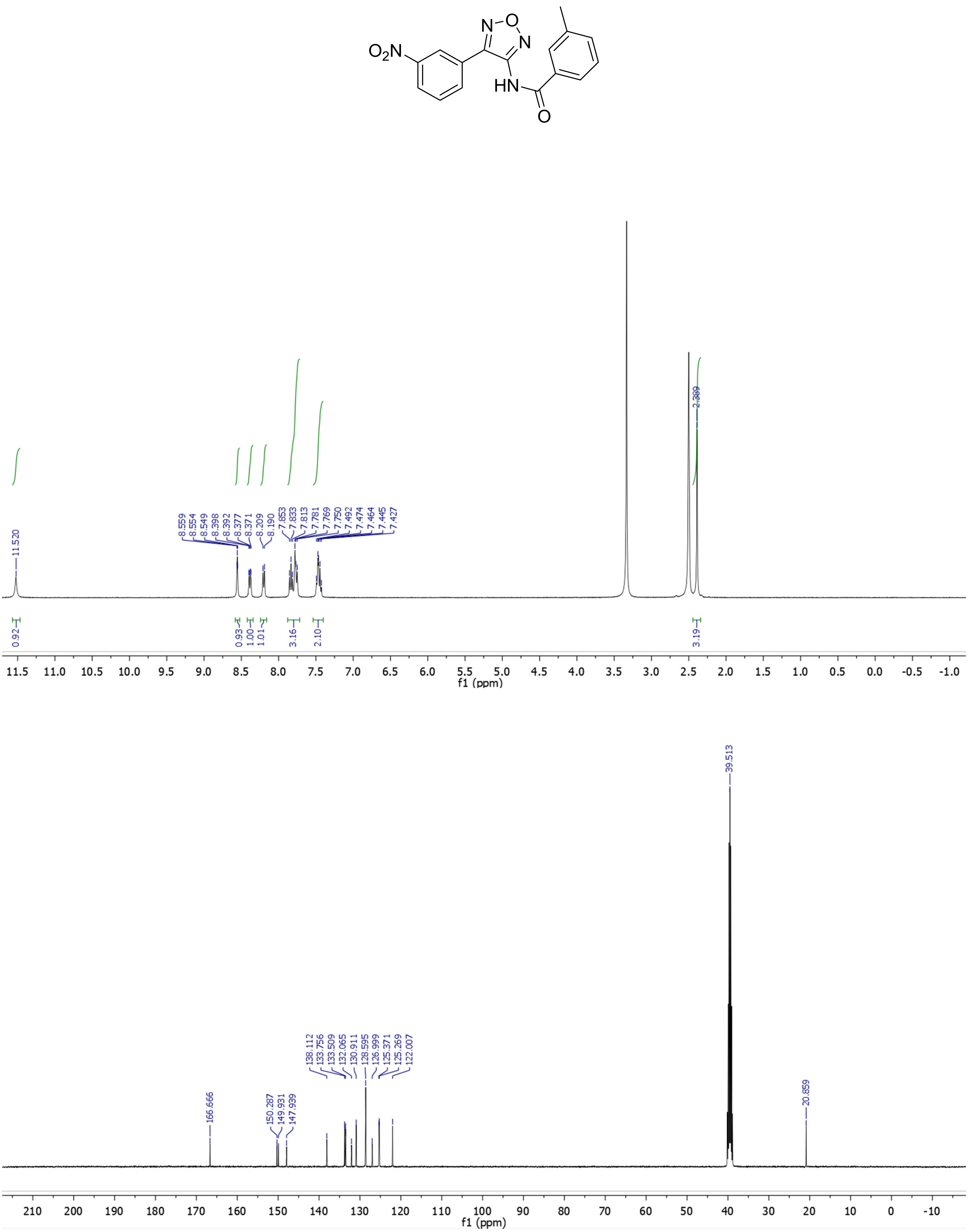

Figure S18. ^{1}H NMR at 400 MHz and ^{13}C NMR at 100 MHz spectra for compound **43**.

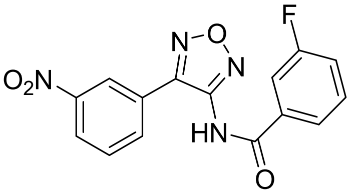

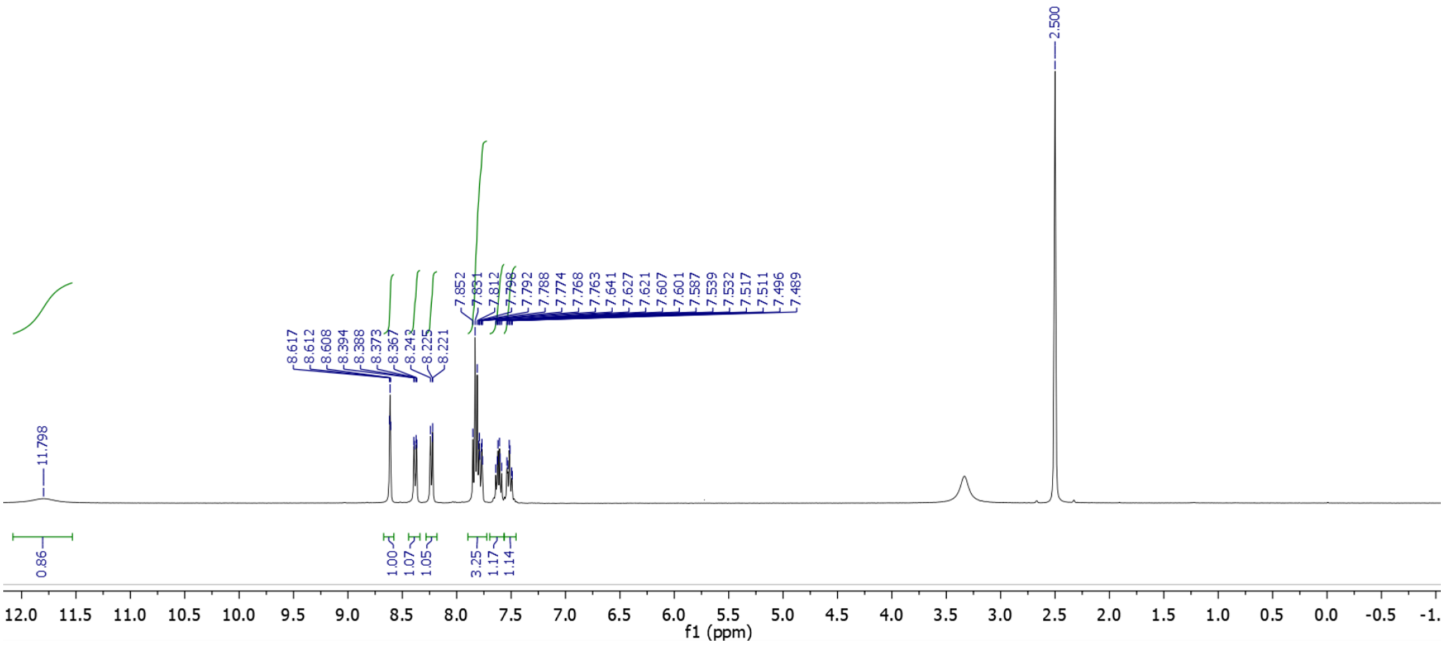

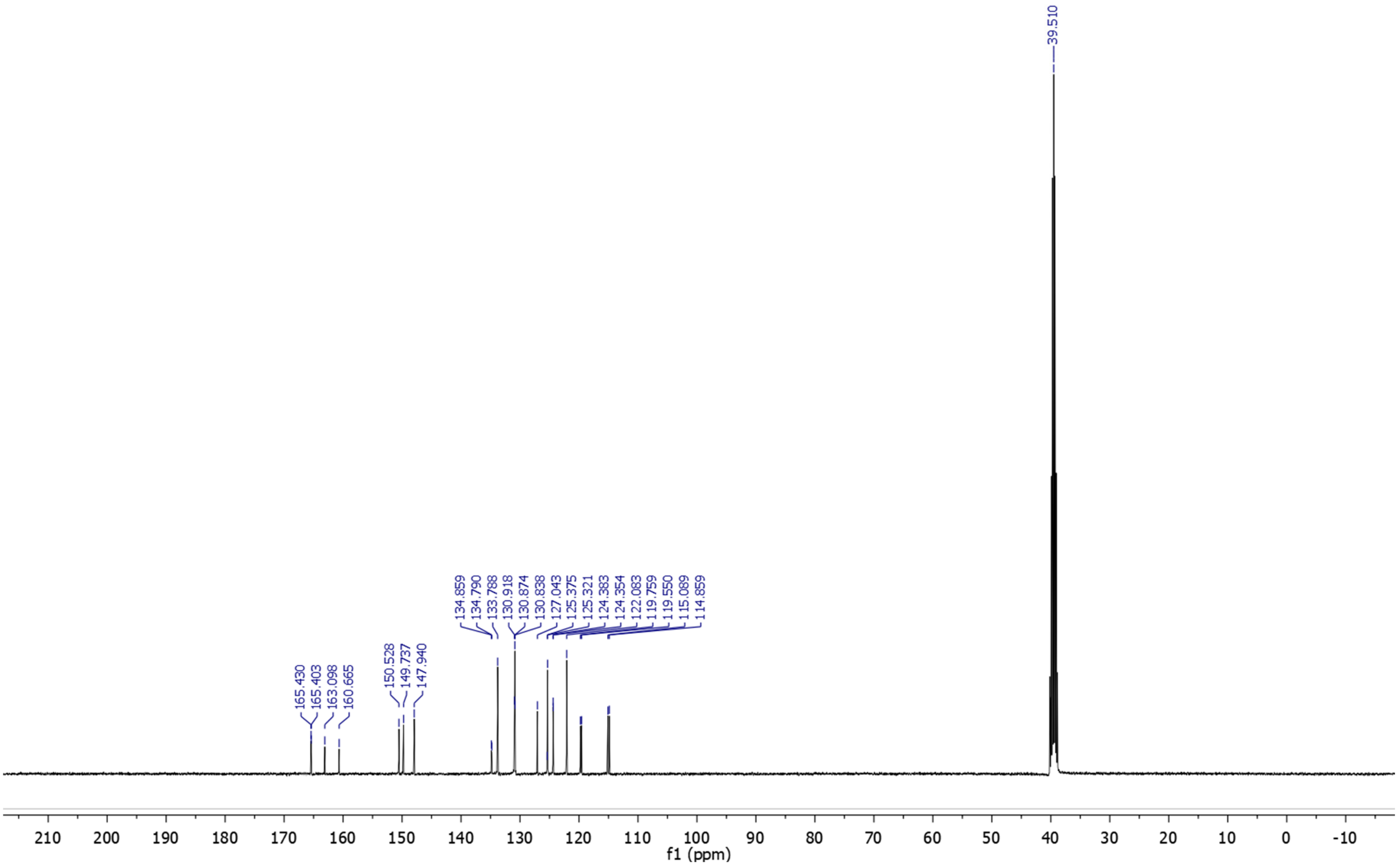

Figure S19. ^{1}H NMR at 400 MHz and ^{13}C NMR at 100 MHz spectra for compound **44**.

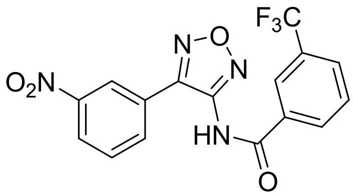

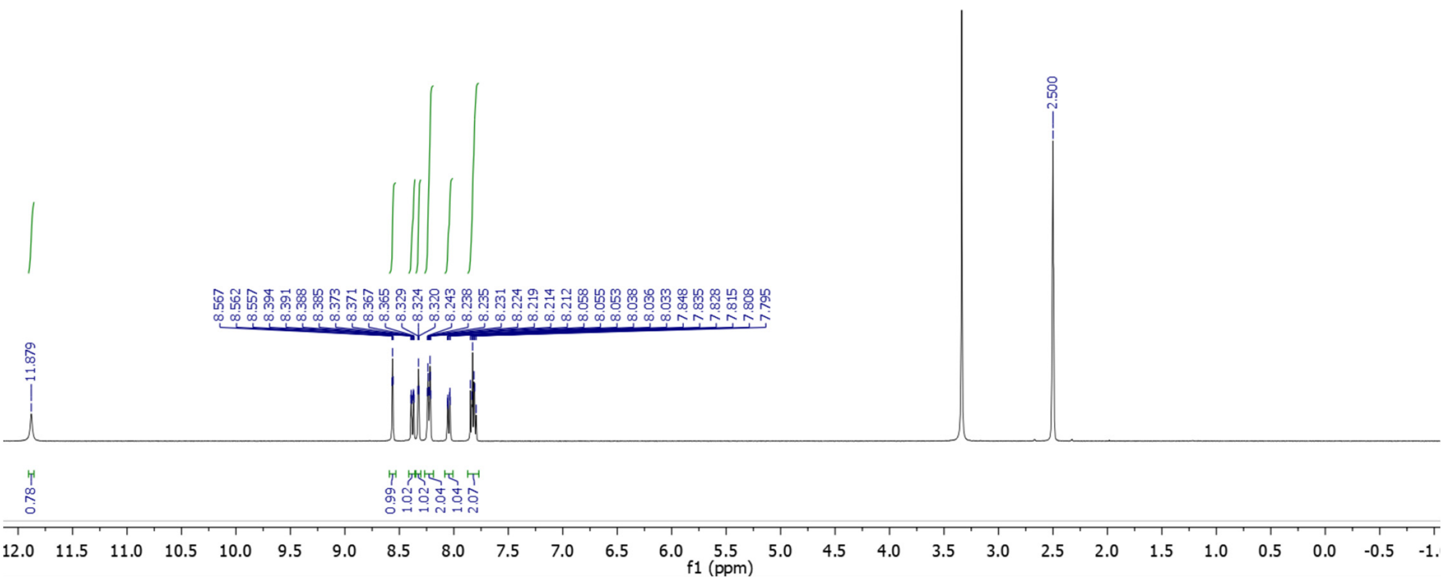

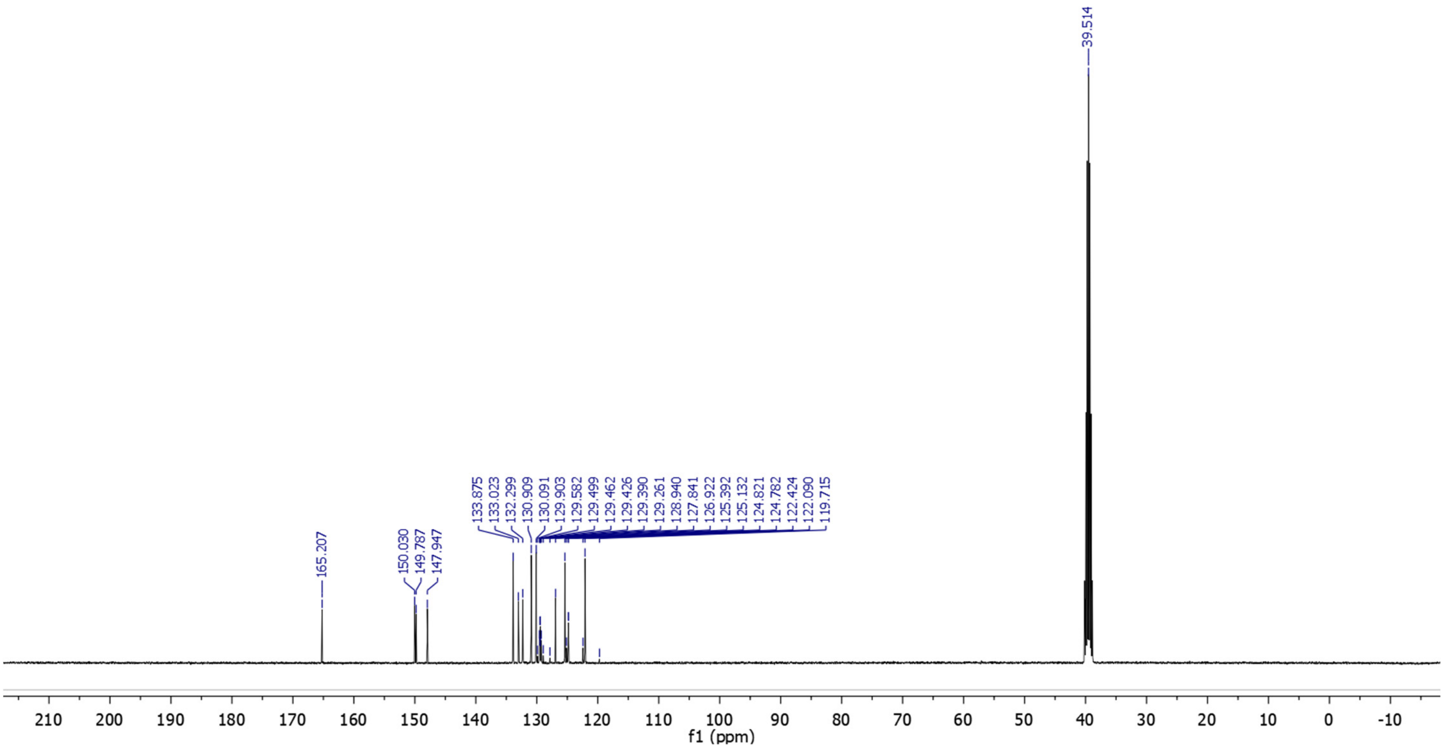

Figure S20. ^{1}H NMR at 400 MHz and ^{13}C NMR at 100 MHz spectra for compound **45**.

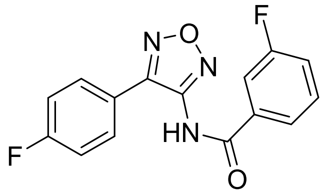

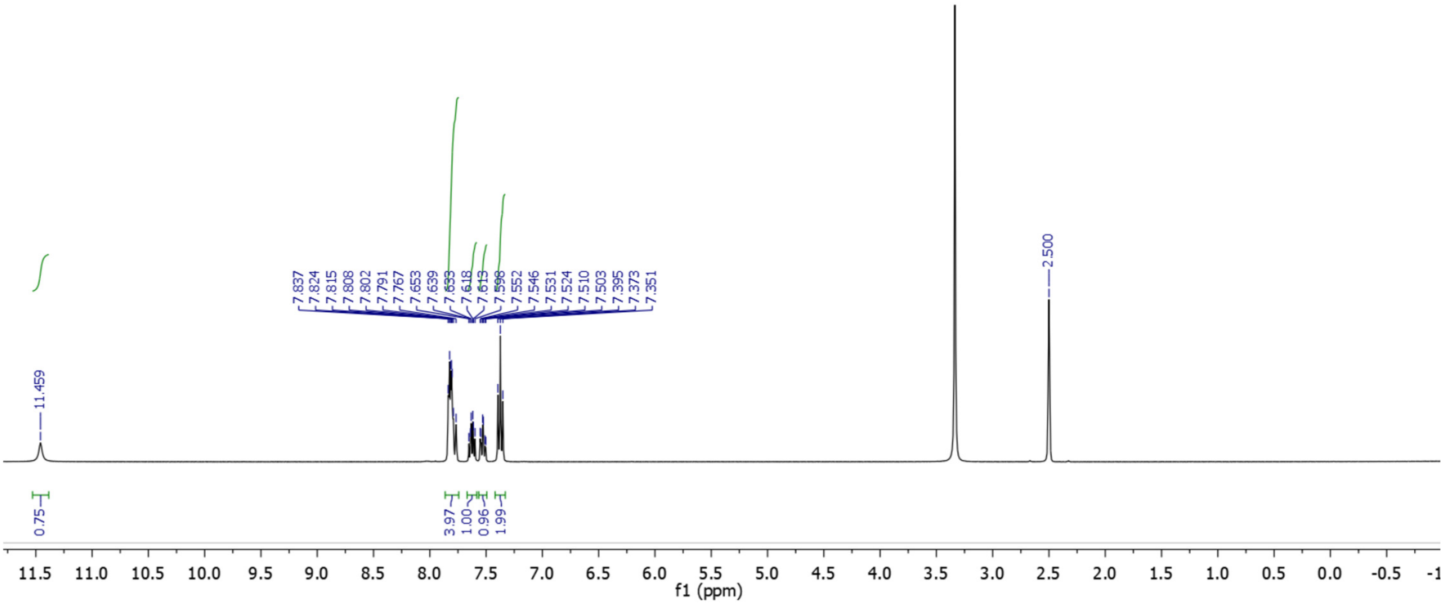

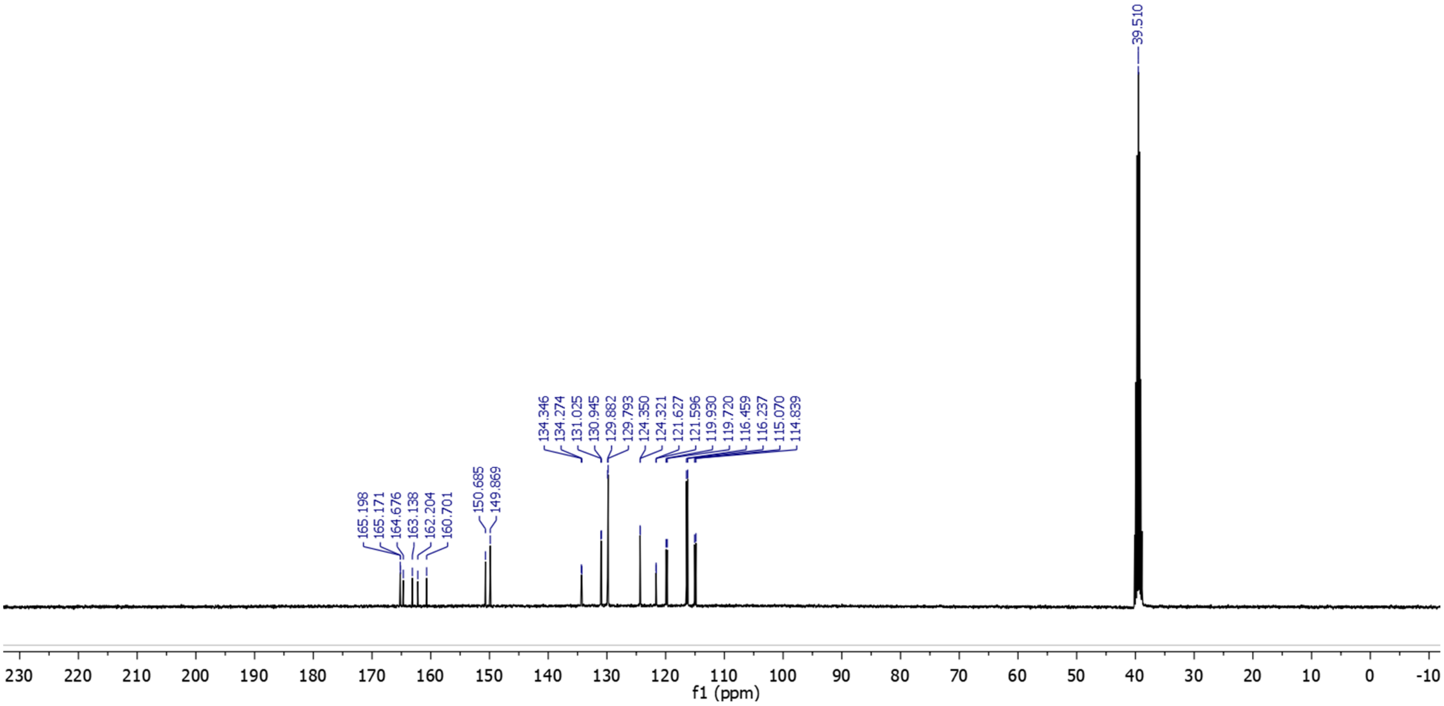

Figure S21. ^{1}H NMR at 400 MHz and ^{13}C NMR at 100 MHz spectra for compound **46**.

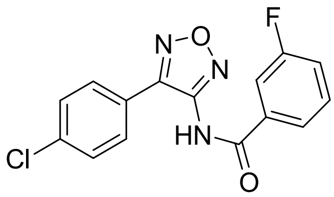

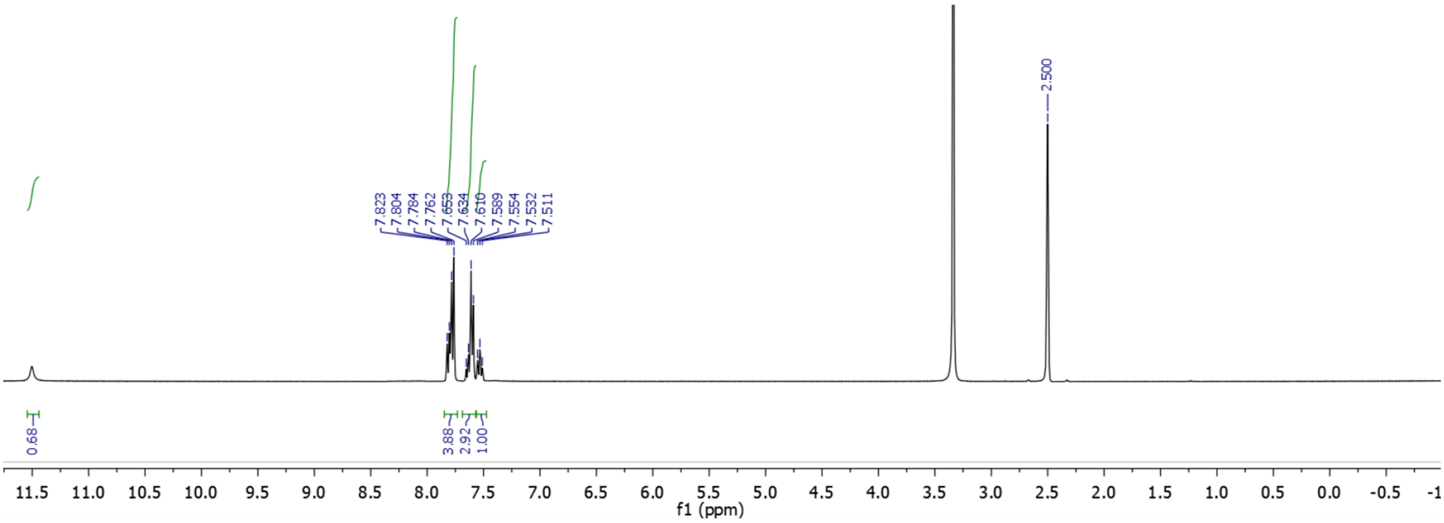

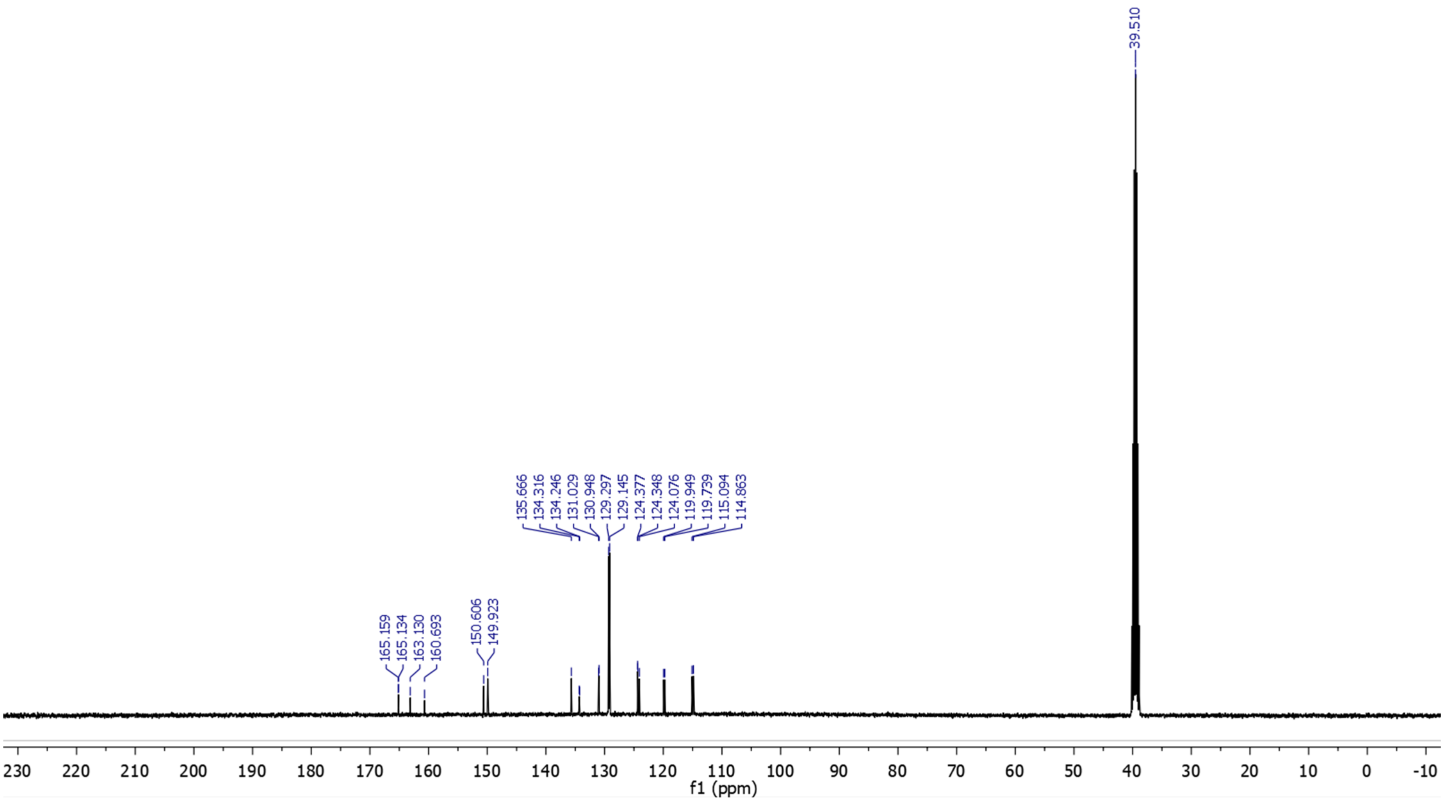

Figure S22. ^{1}H NMR at 400 MHz and ^{13}C NMR at 100 MHz spectra for compound **47**.

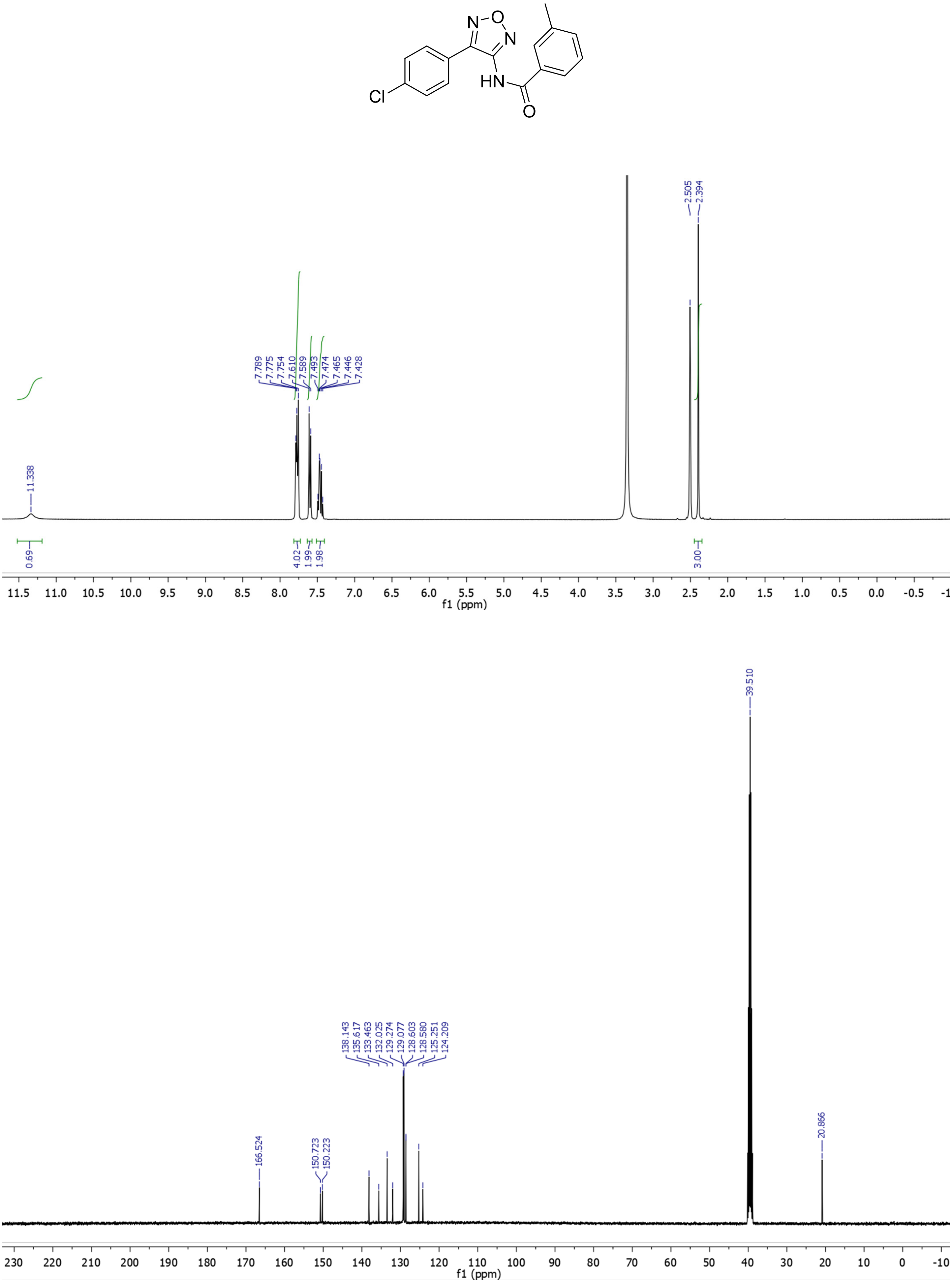

Figure S23. ^{1}H NMR at 400 MHz and ^{13}C NMR at 100 MHz spectra for compound **49**.

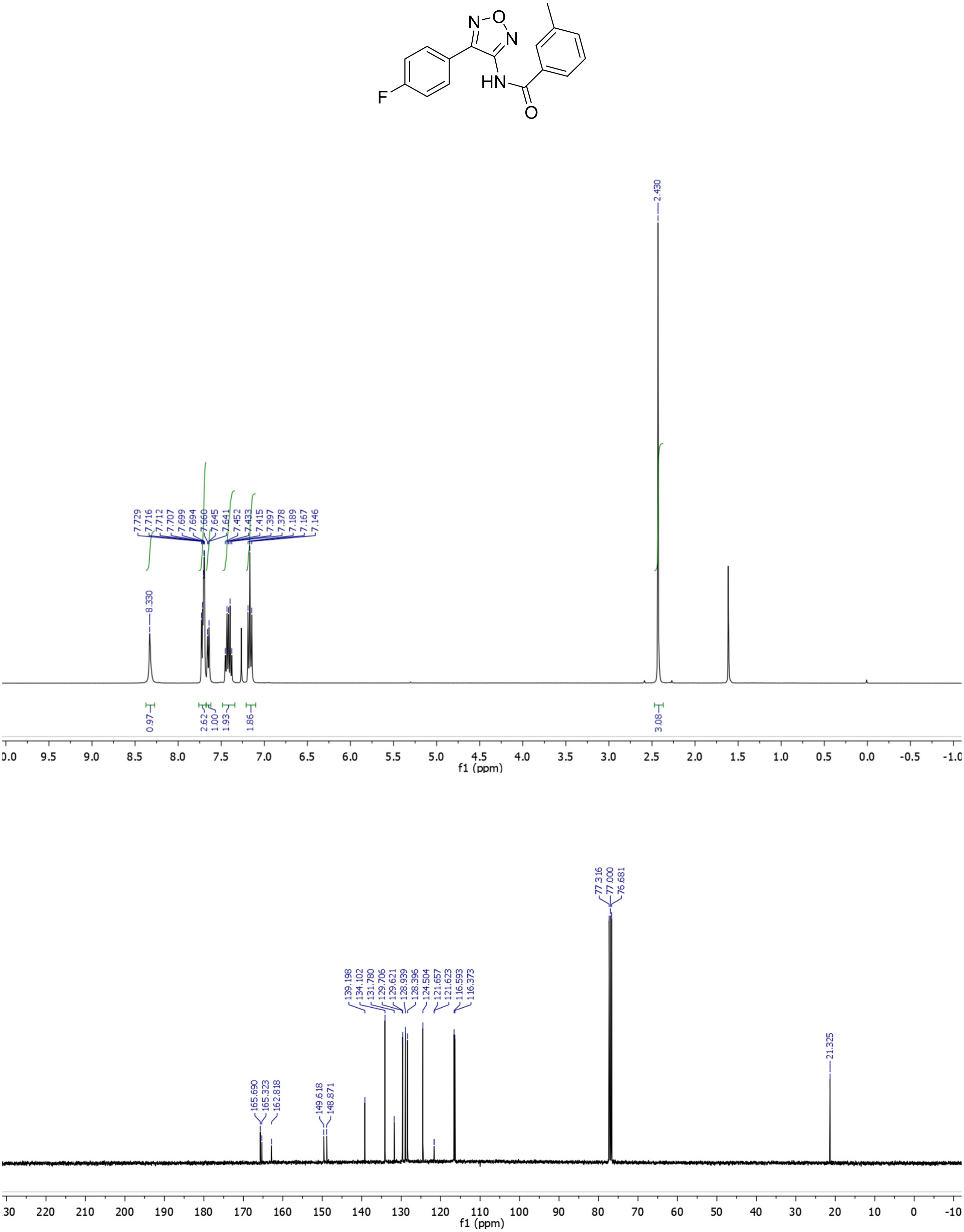

Figure S24. ^{1}H NMR at 400 MHz and ^{13}C NMR at 100 MHz spectra for compound **50**.

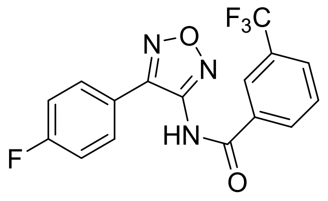

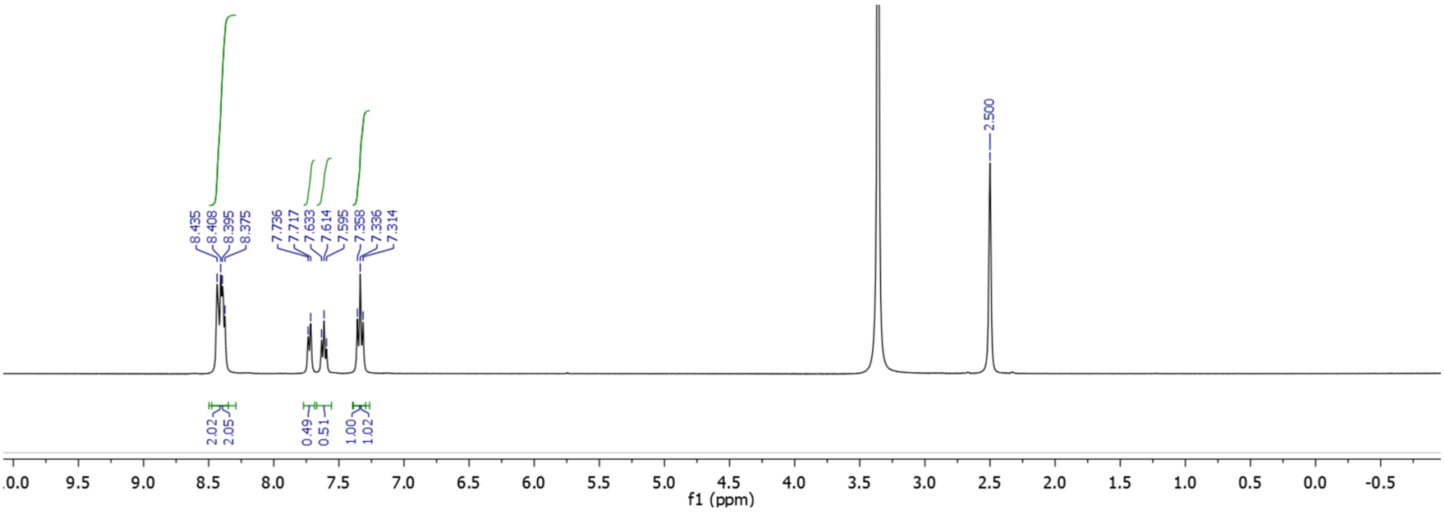

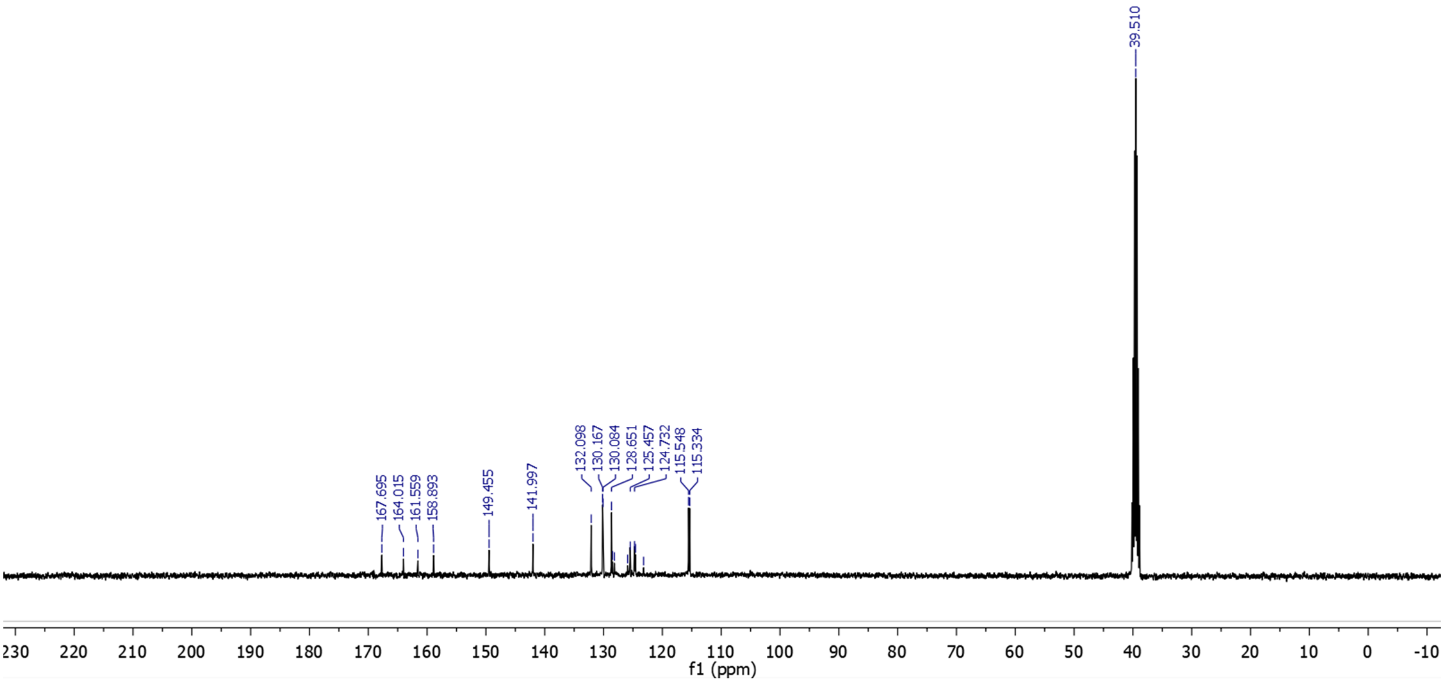

Figure S25. ^{1}H NMR at 400 MHz and ^{13}C NMR at 100 MHz spectra for compound **51**.

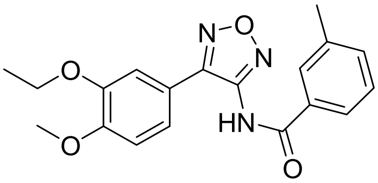

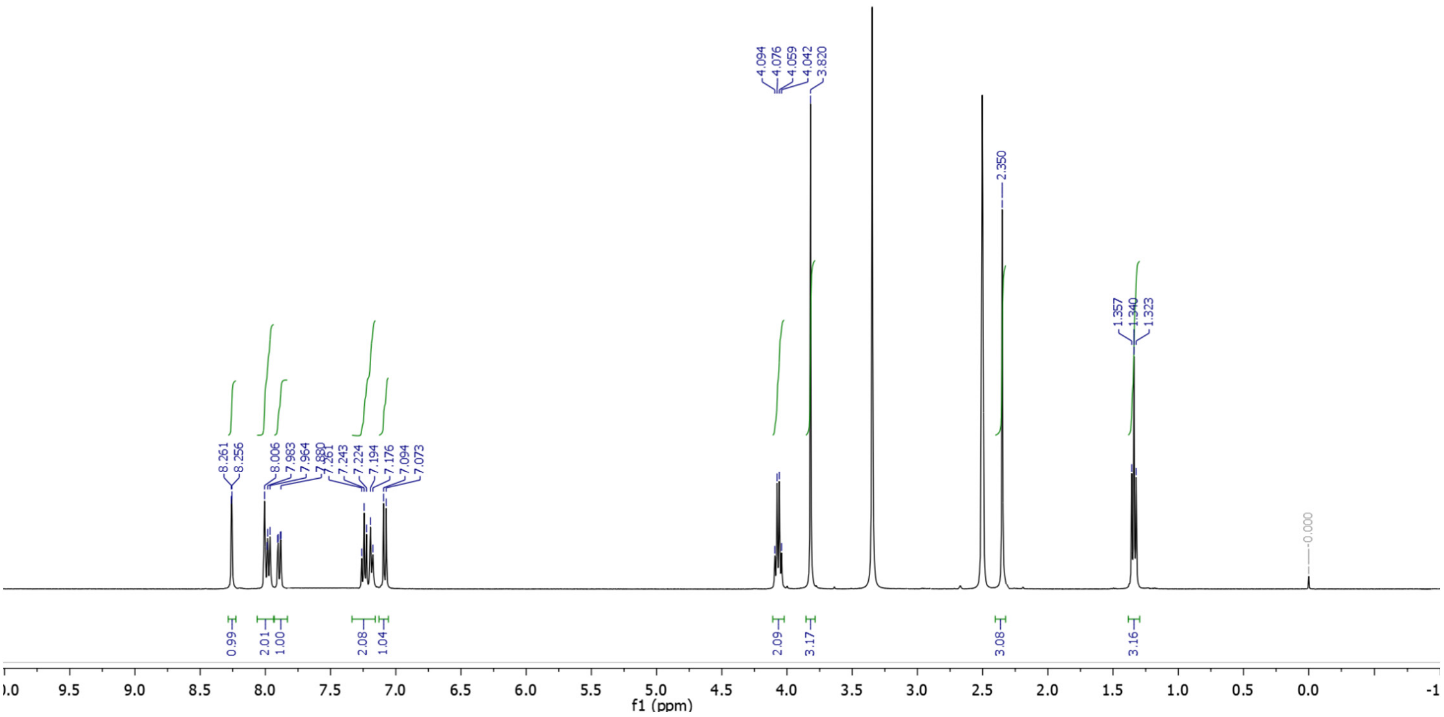

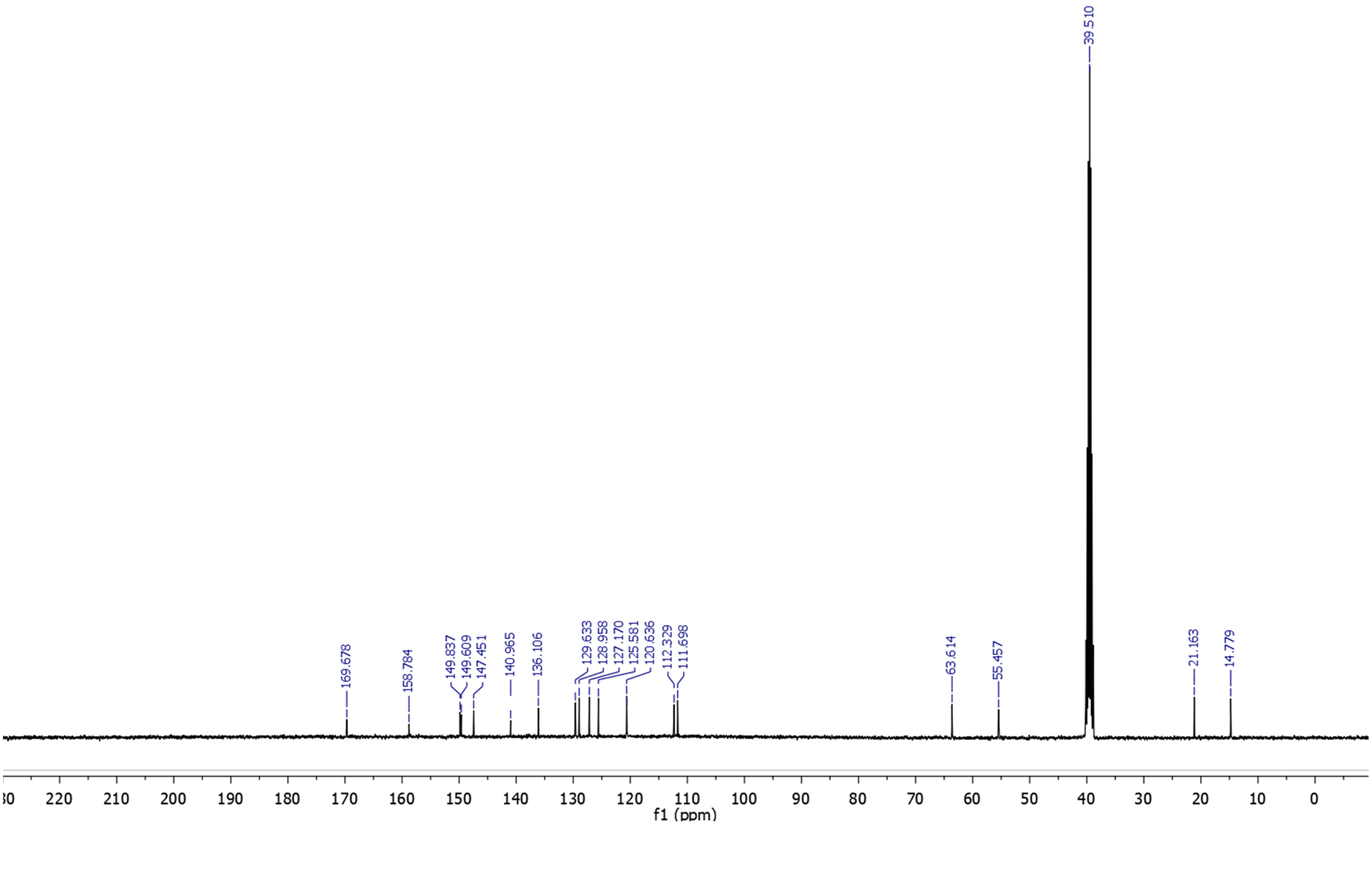

Figure S26. ${}^{1}H$ NMR at 400 MHz and ${}^{13}C$ NMR at 100 MHz spectra for compound **52**.

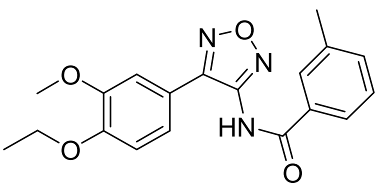

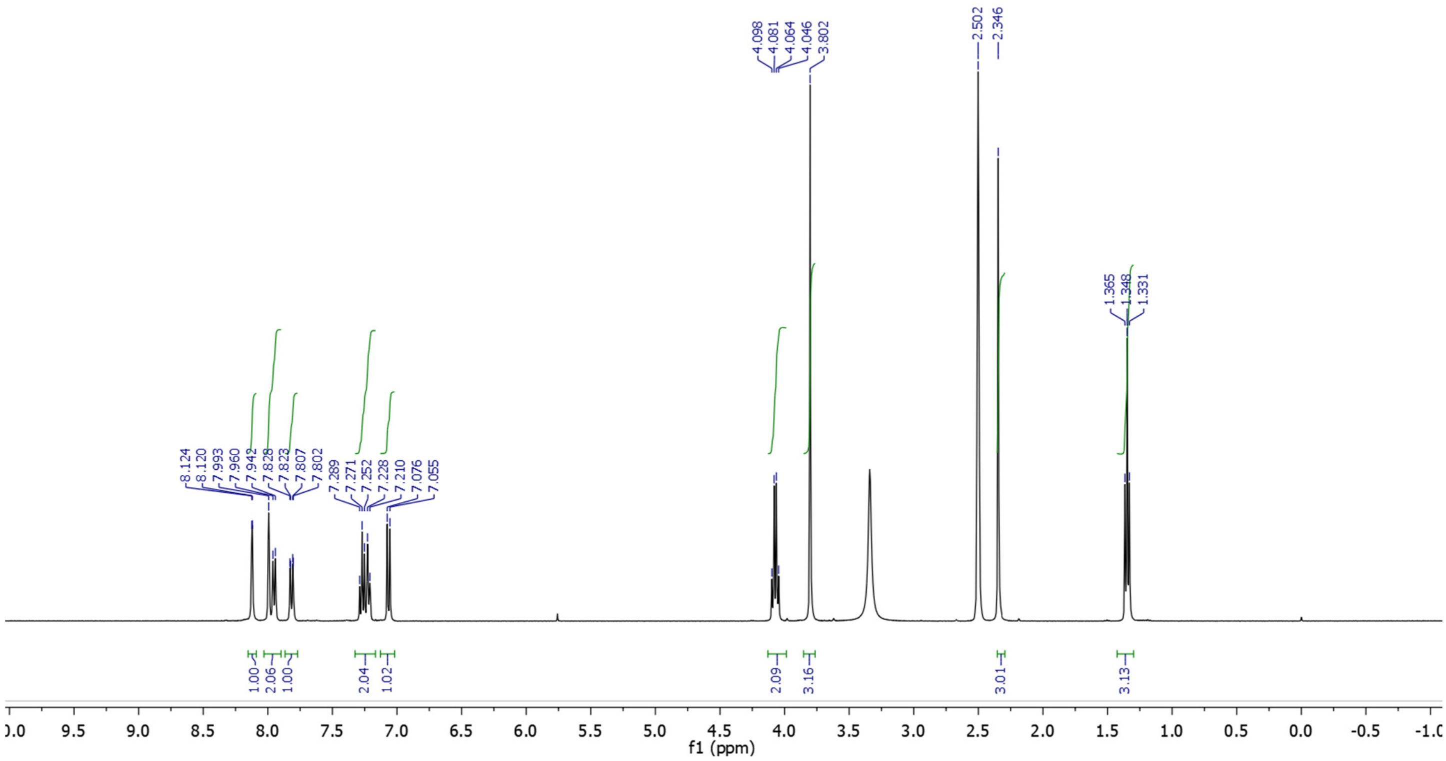

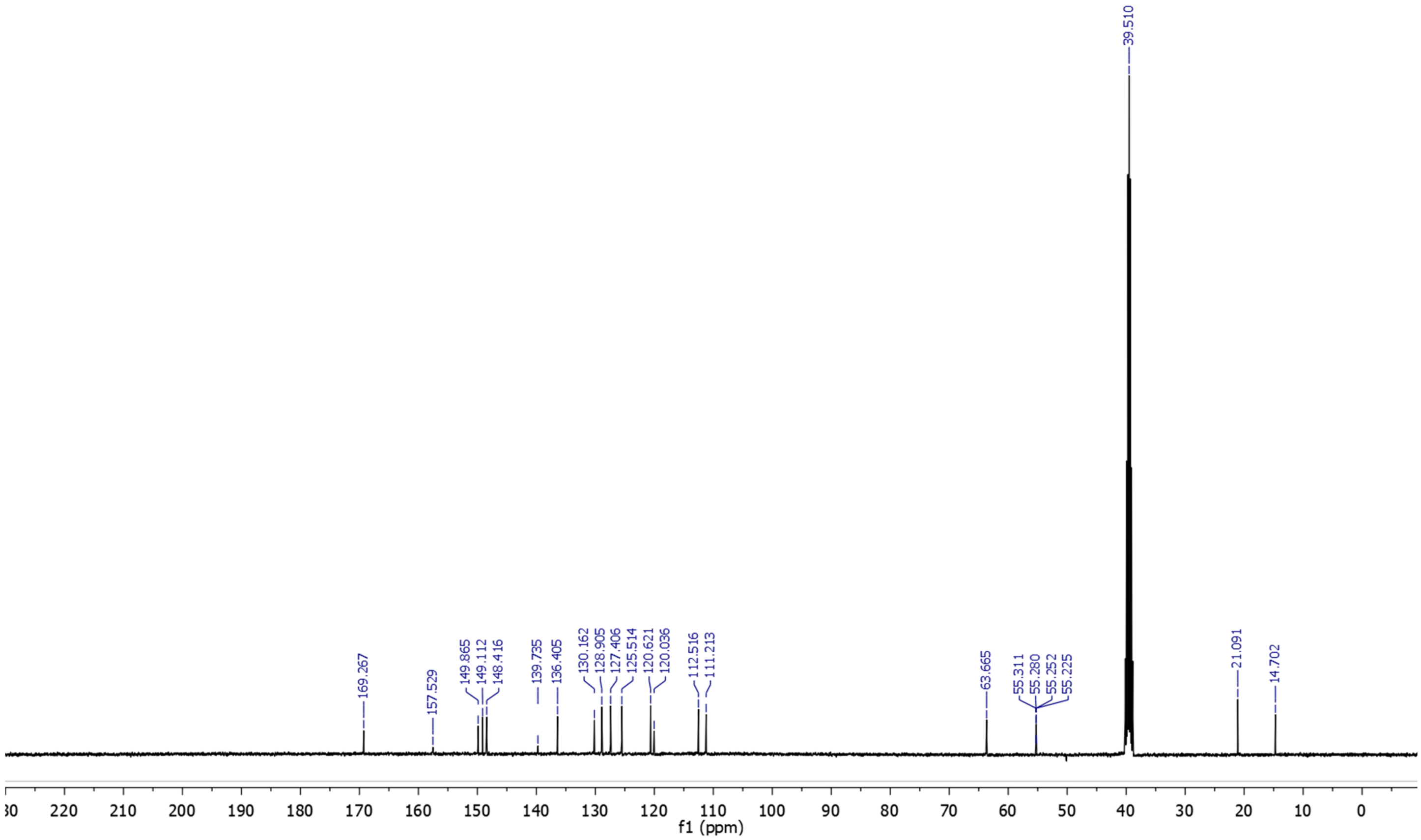

Figure S27. ^{1}H NMR at 400 MHz and ^{13}C NMR at 100 MHz spectra for compound **53**.

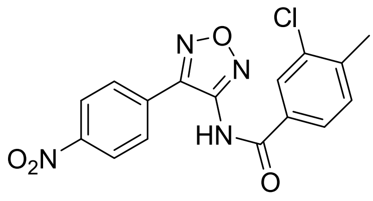

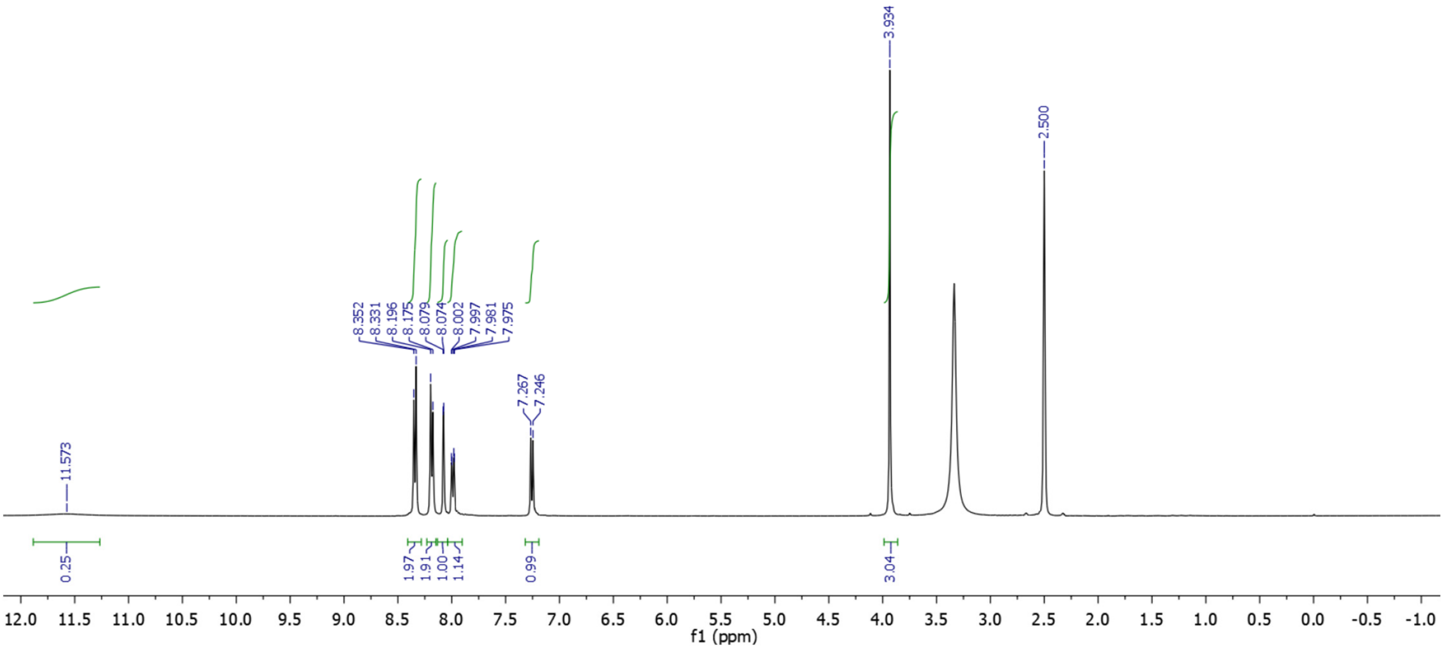

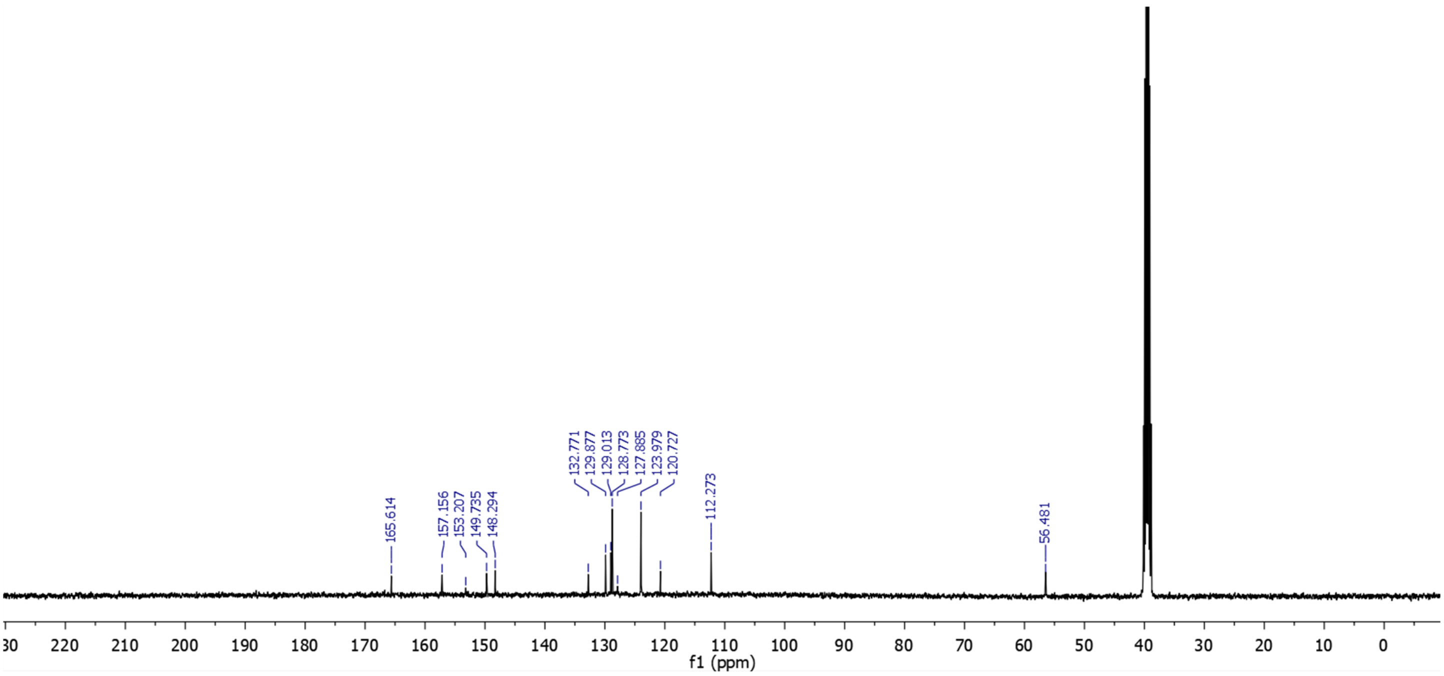

Figure S28. ^{1}H NMR at 400 MHz and ^{13}C NMR at 100 MHz spectra for compound **54**.

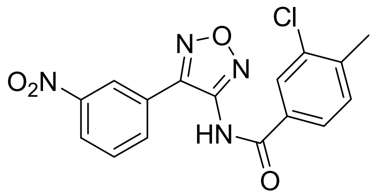

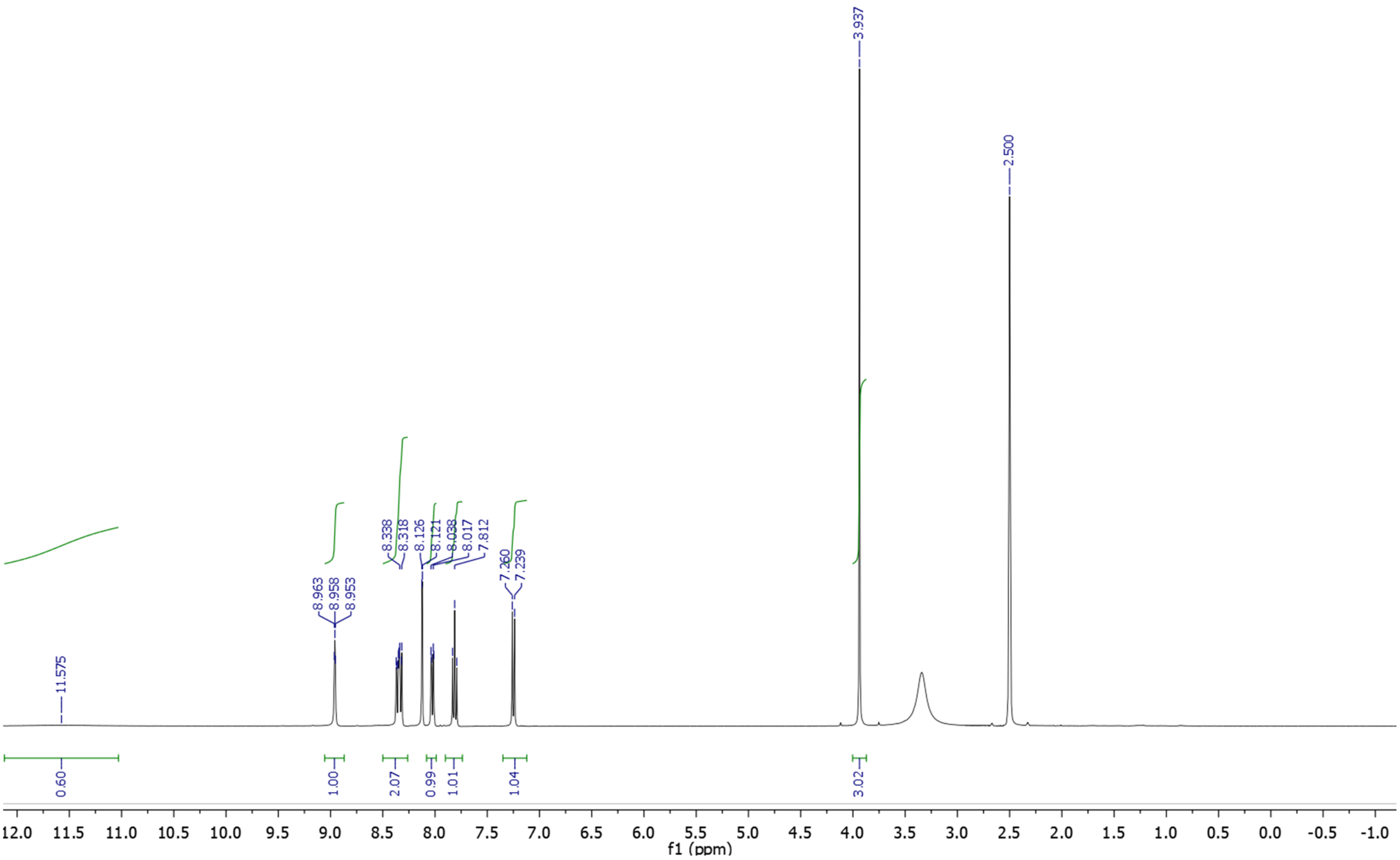

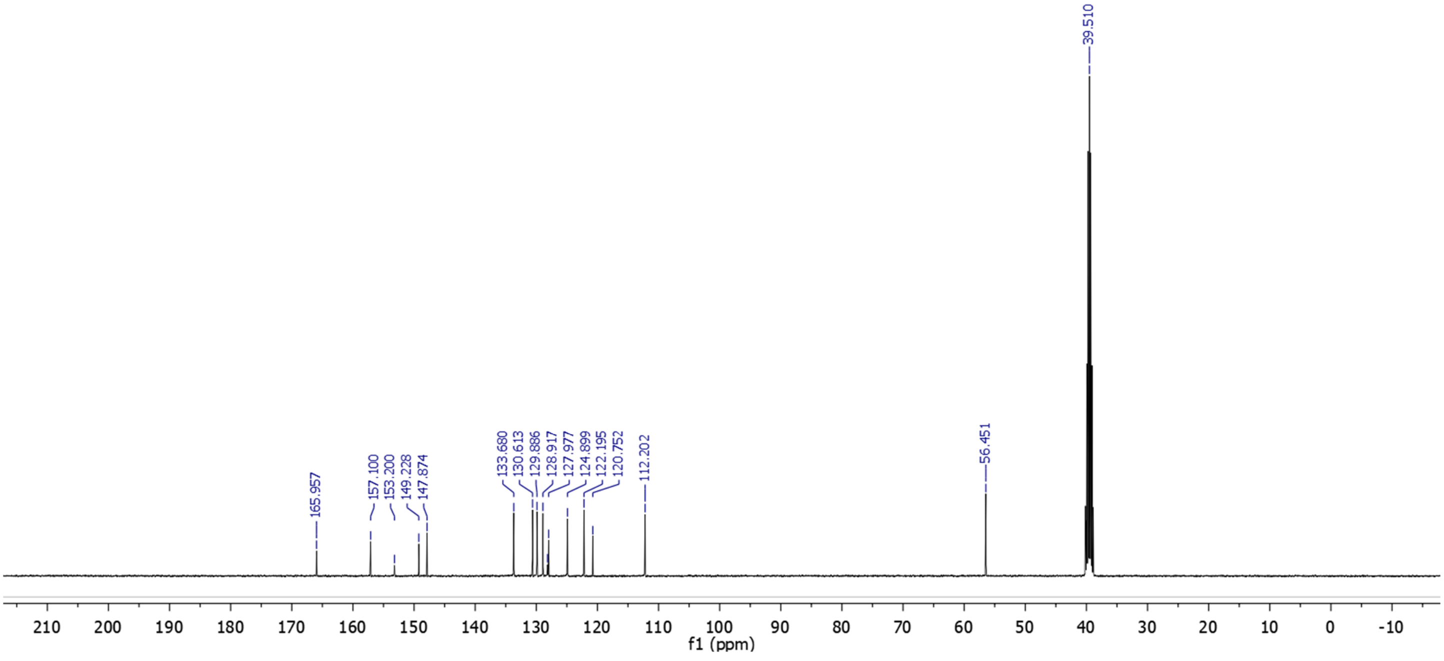

Figure S29. ^{1}H NMR at 400 MHz and ^{13}C NMR at 100 MHz spectra for compound **55**.

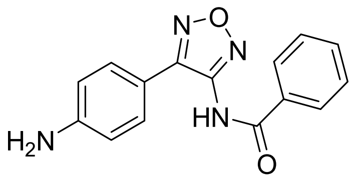

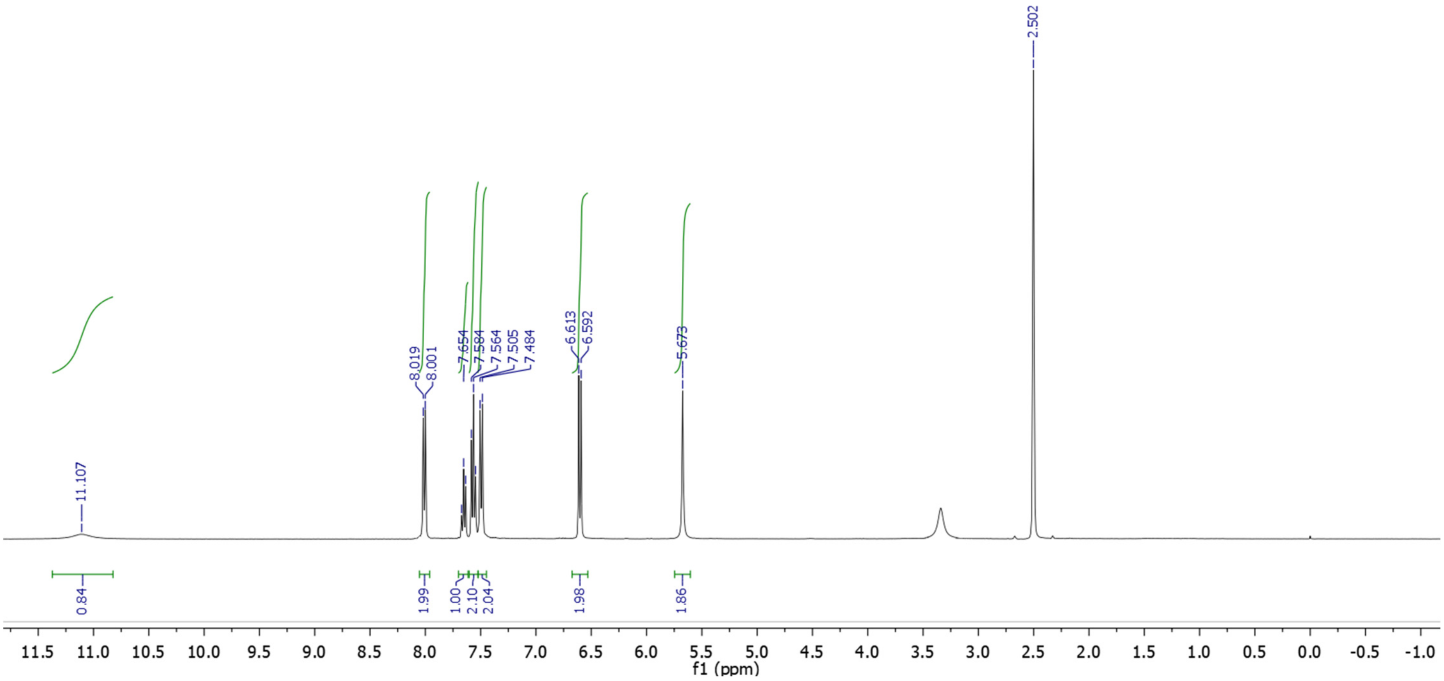

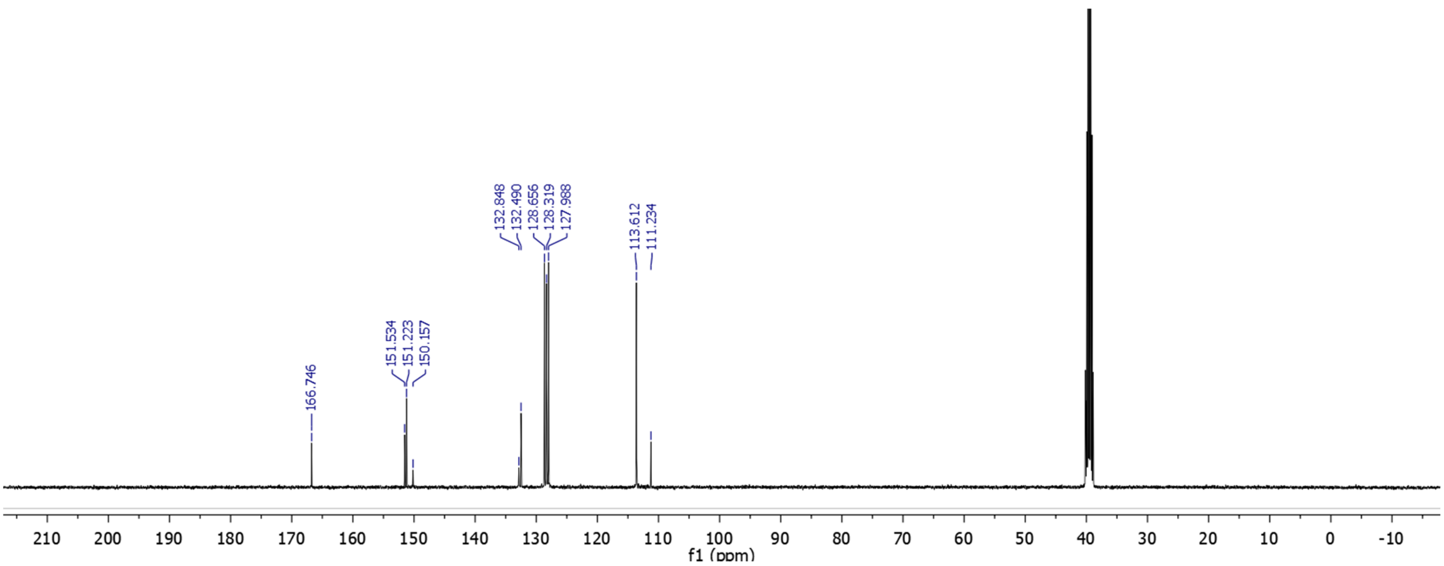

Figure S30. ^{1}H NMR at 400 MHz and ^{13}C NMR at 100 MHz spectra for compound **56**.

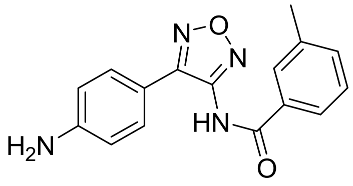

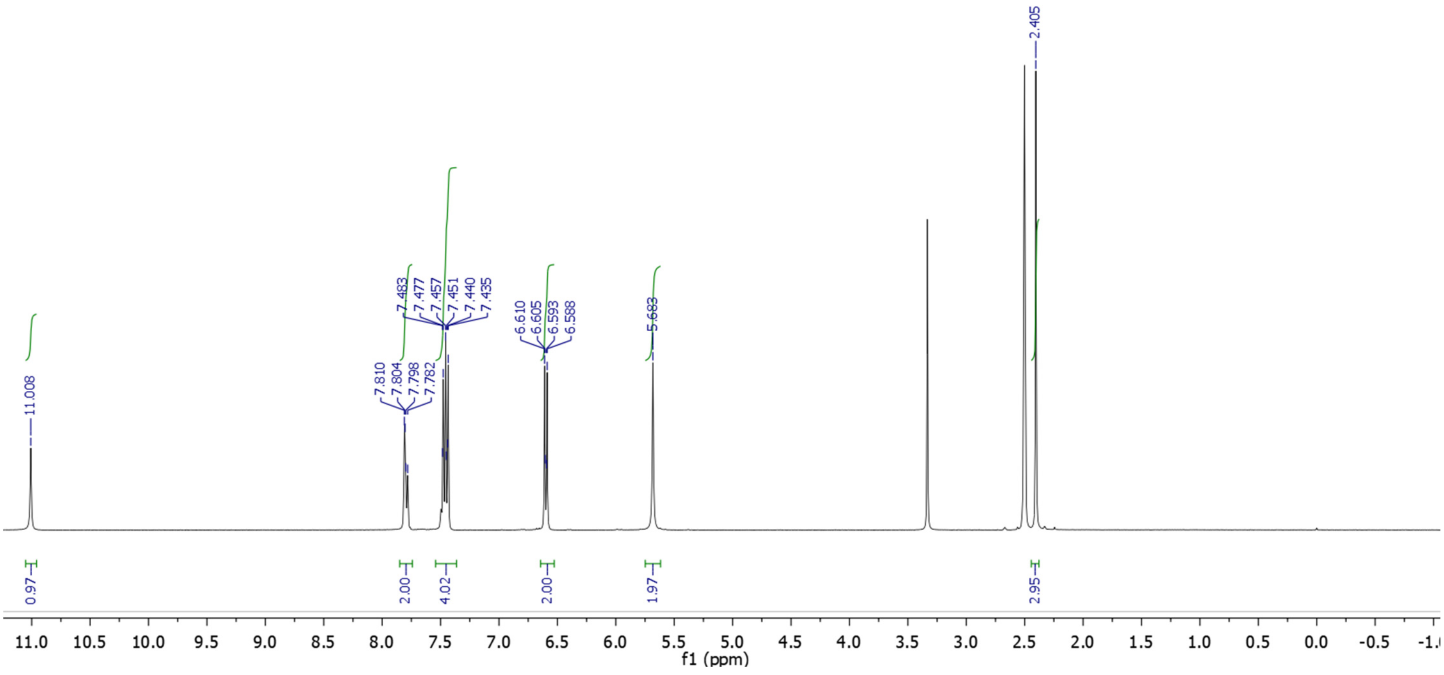

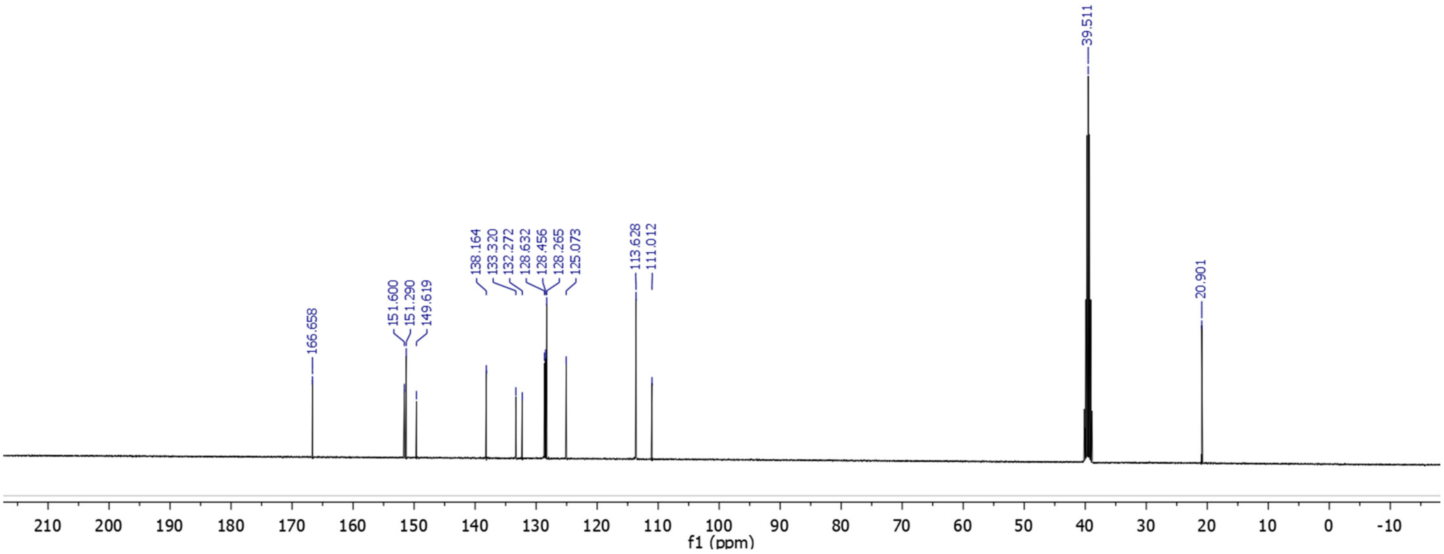

Figure S31. ^{1}H NMR at 400 MHz and ^{13}C NMR at 100 MHz spectra for compound **57**.

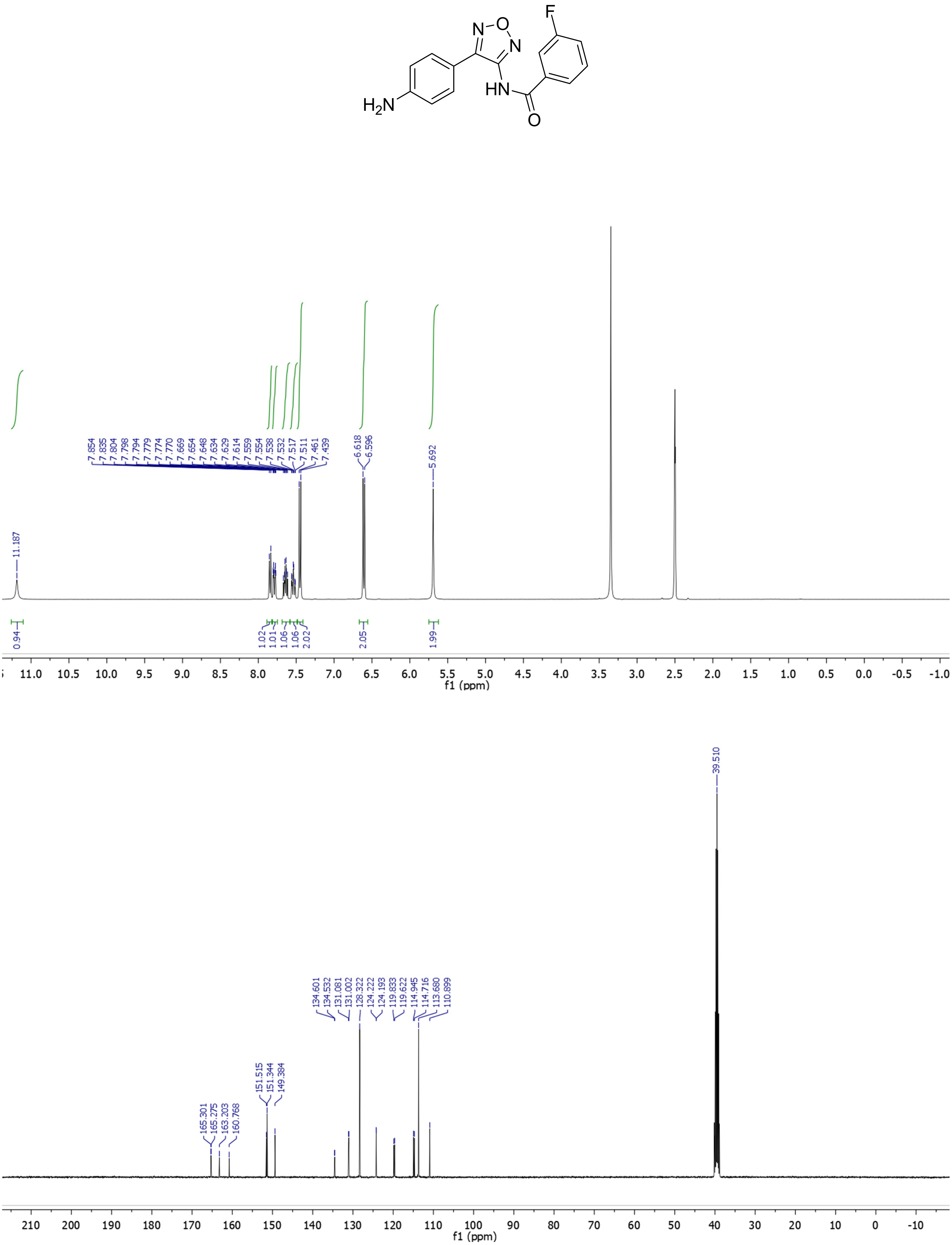

Figure S32. ^{1}H NMR at 400 MHz and ^{13}C NMR at 100 MHz spectra for compound **58**.

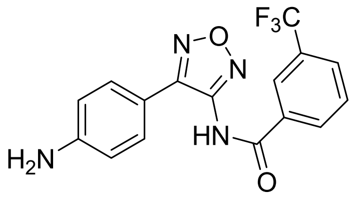

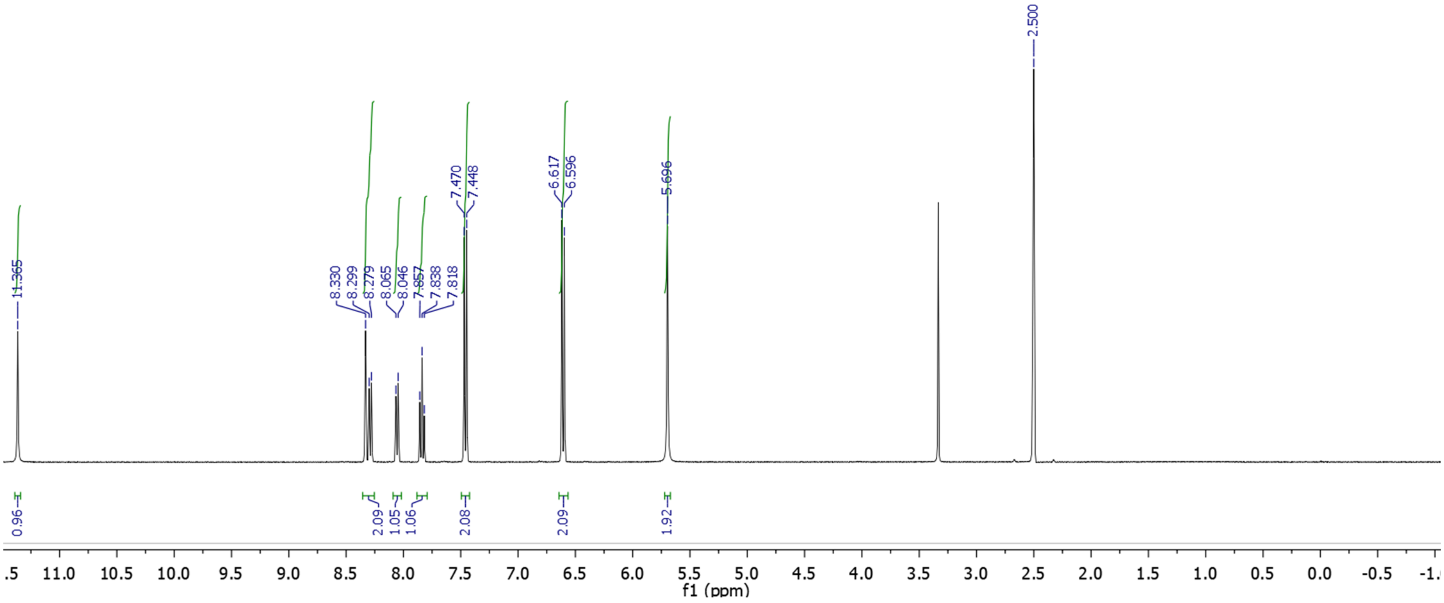

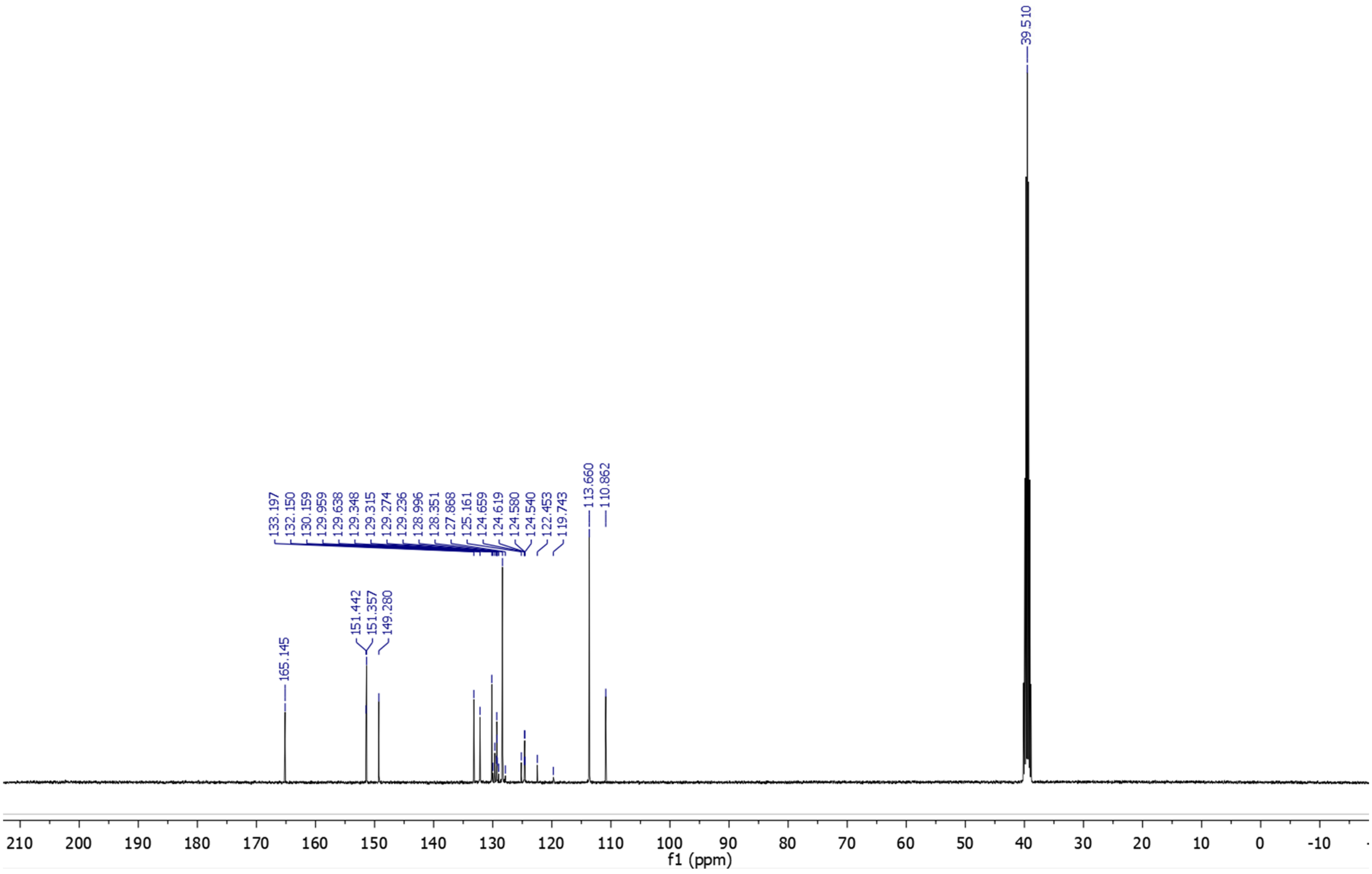

Figure S33. ^{1}H NMR at 400 MHz and ^{13}C NMR at 100 MHz spectra for compound **59**.

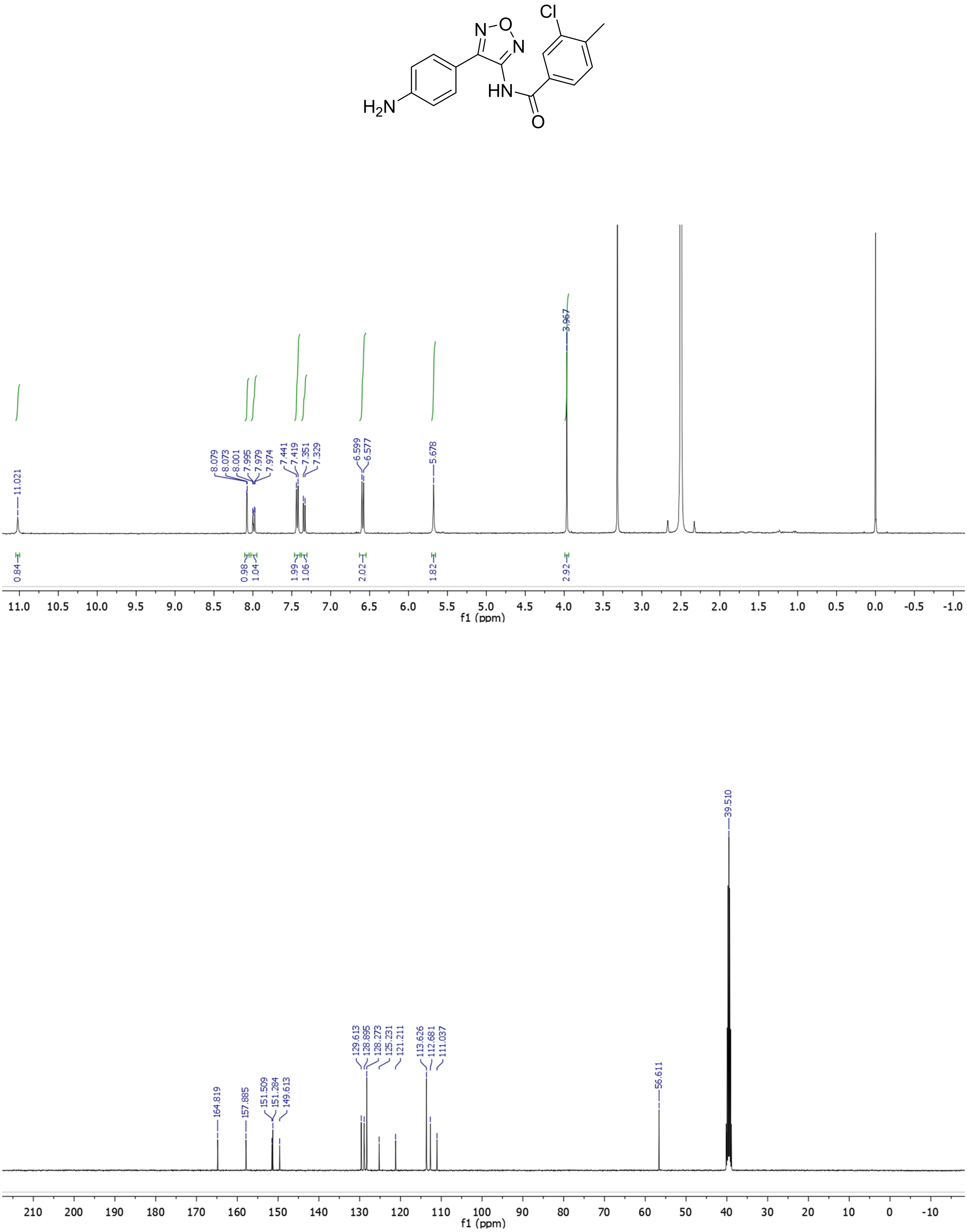

Figure S34. ^{1}H NMR at 400 MHz and ^{13}C NMR at 100 MHz spectra for compound **60**.

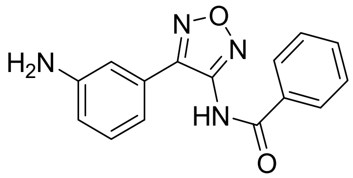

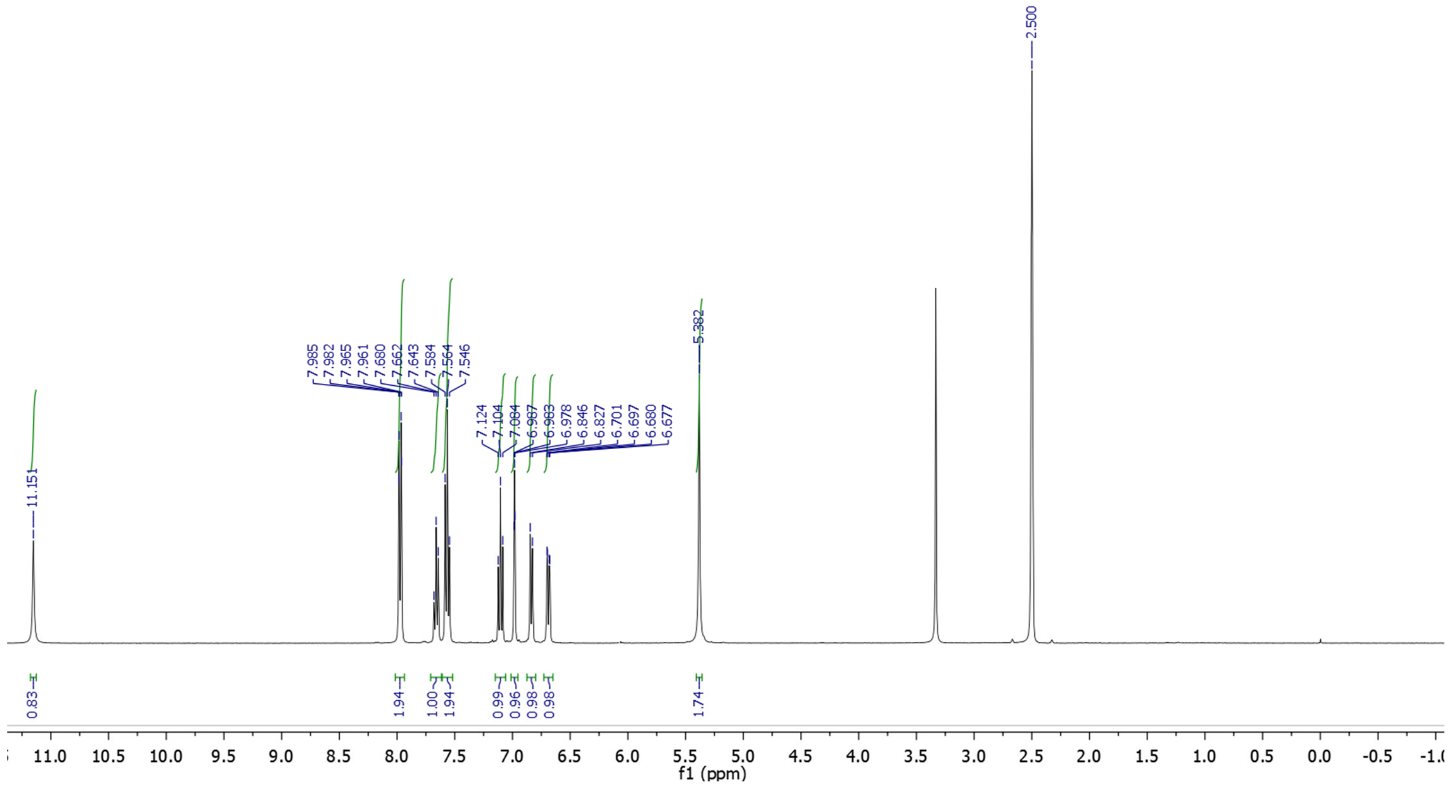

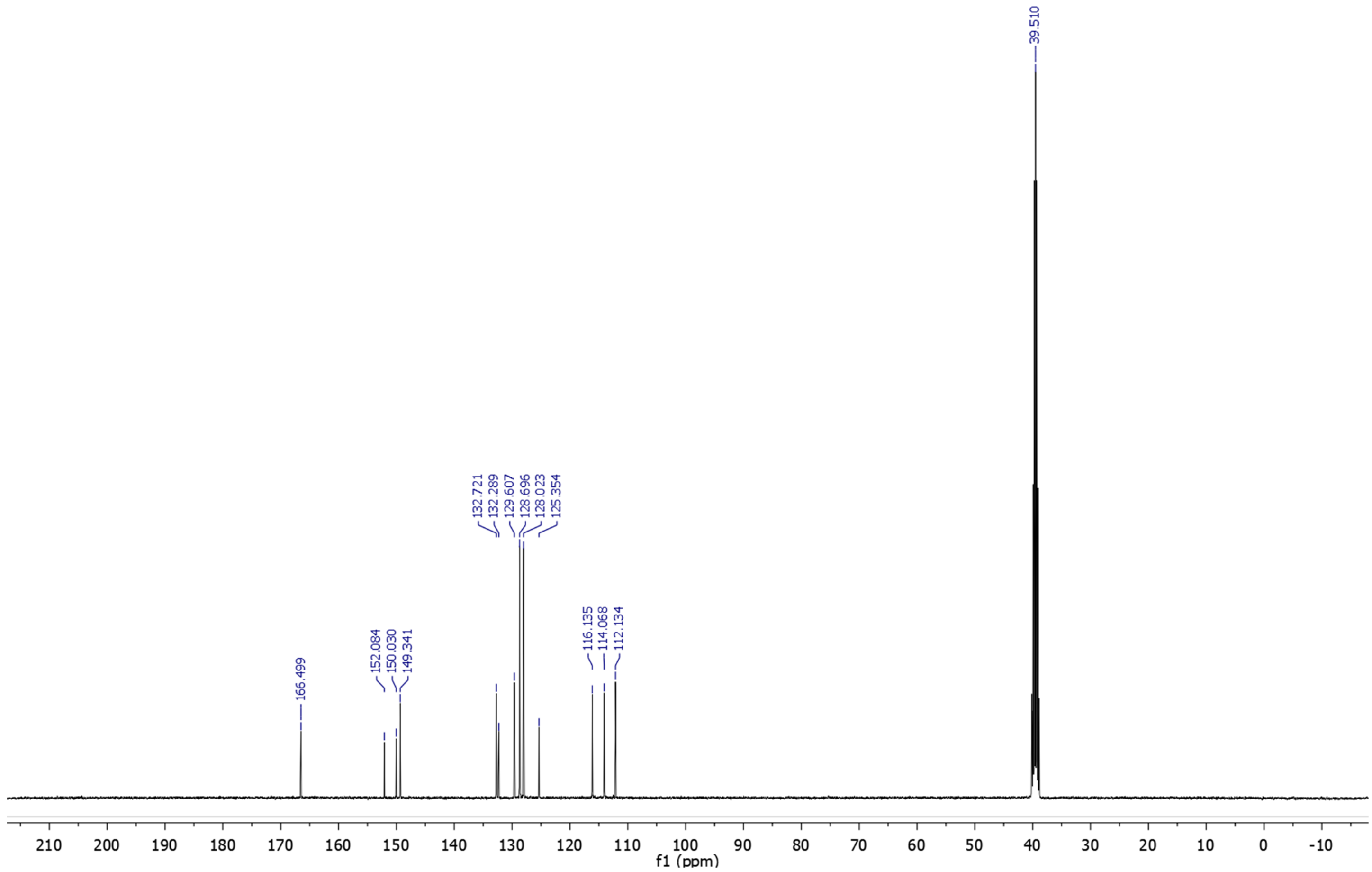

Figure S35. ^{1}H NMR at 400 MHz and ^{13}C NMR at 100 MHz spectra for compound **61**.

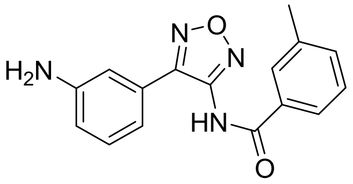

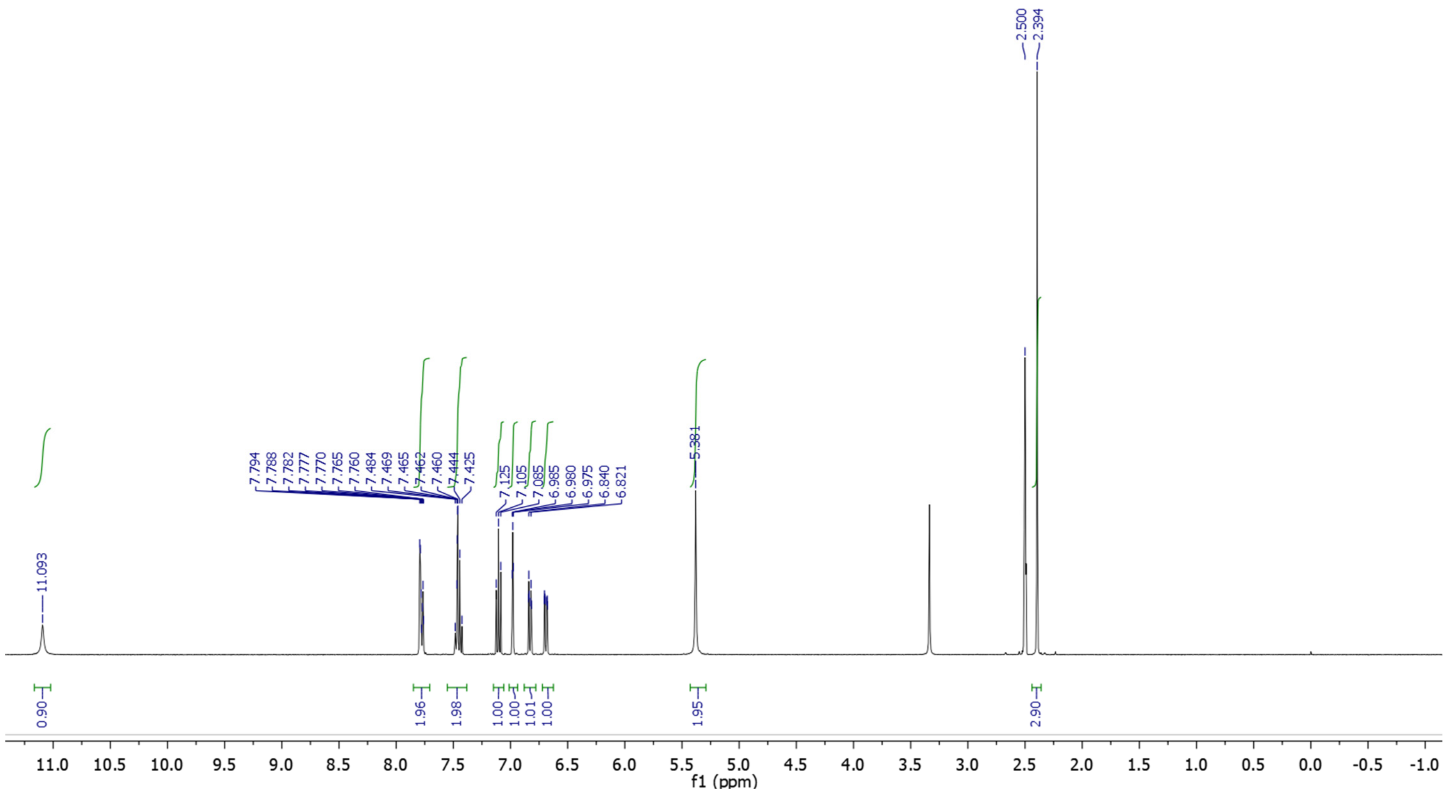

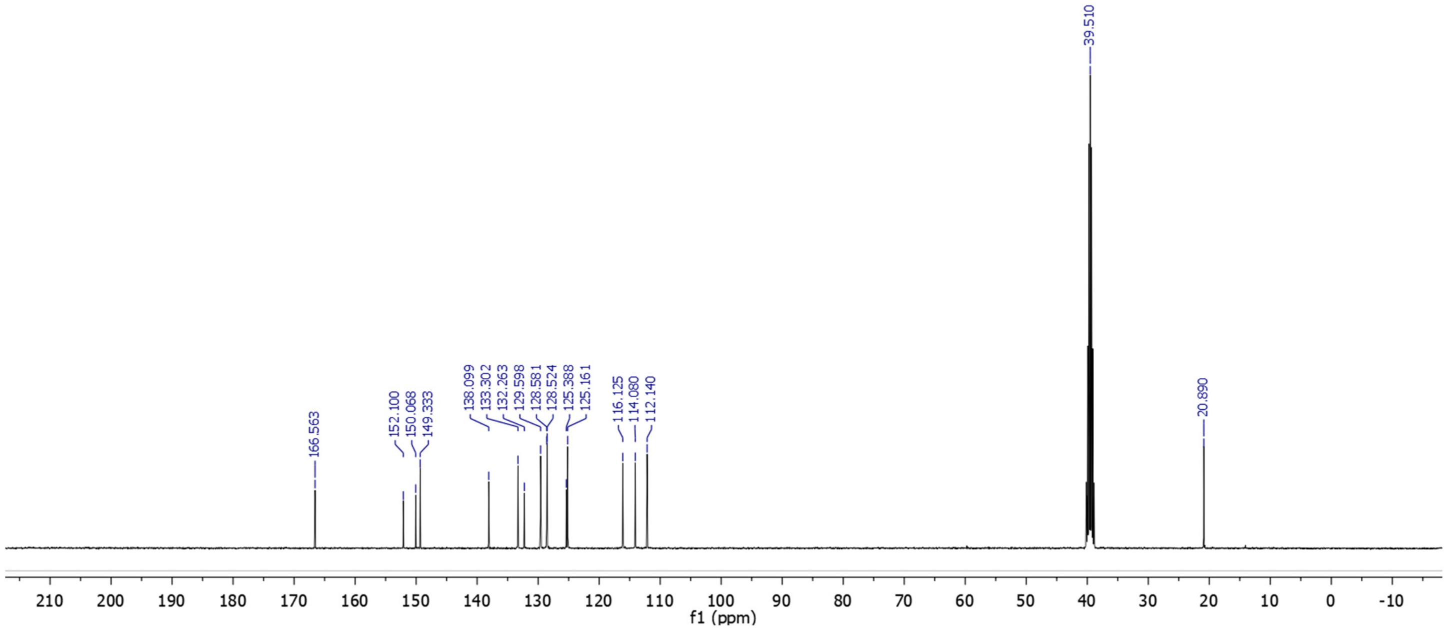

Figure S36. ^{1}H NMR at 400 MHz and ^{13}C NMR at 100 MHz spectra for compound **62**.

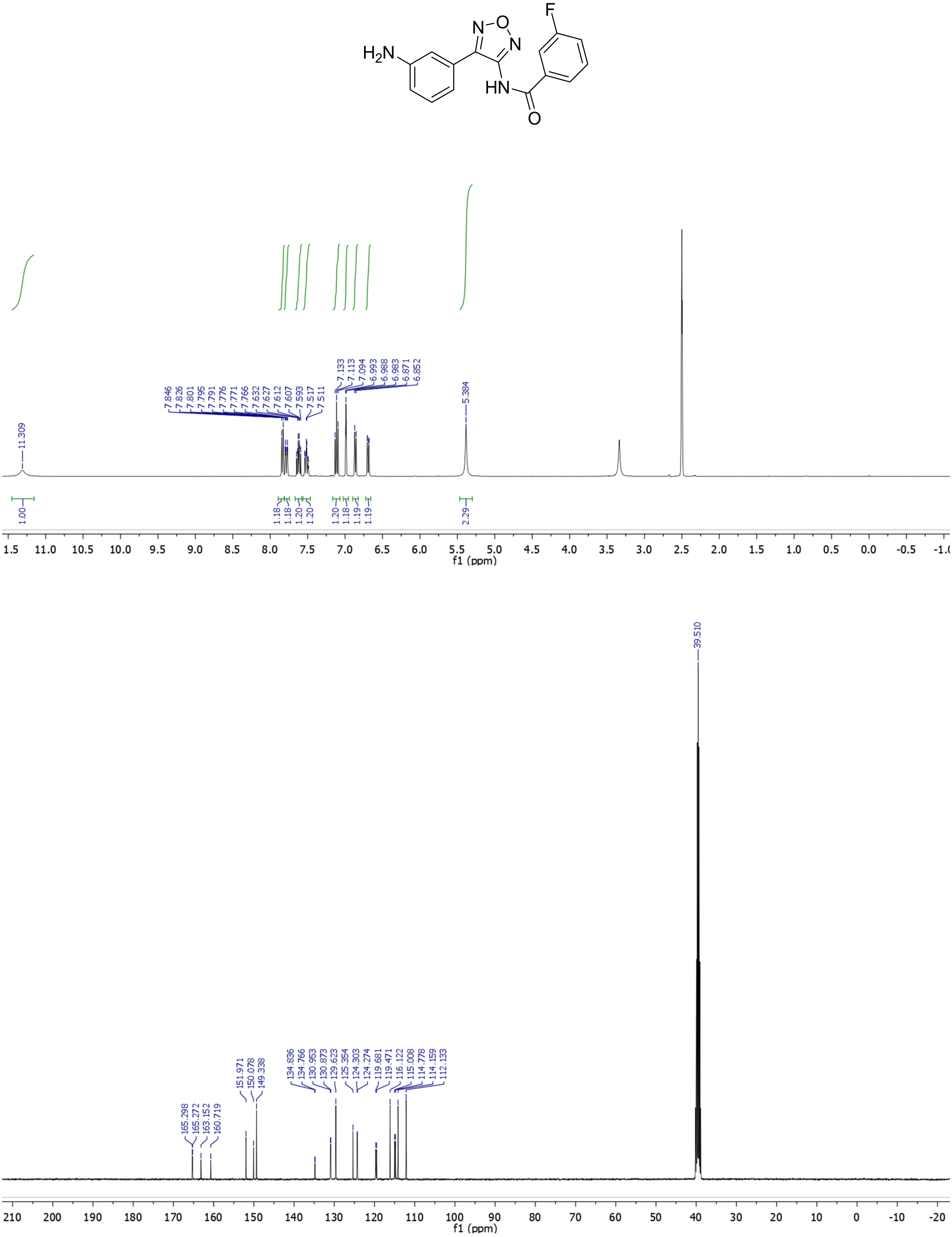

Figure S37. ^{1}H NMR at 400 MHz and ^{13}C NMR at 100 MHz spectra for compound **63**.

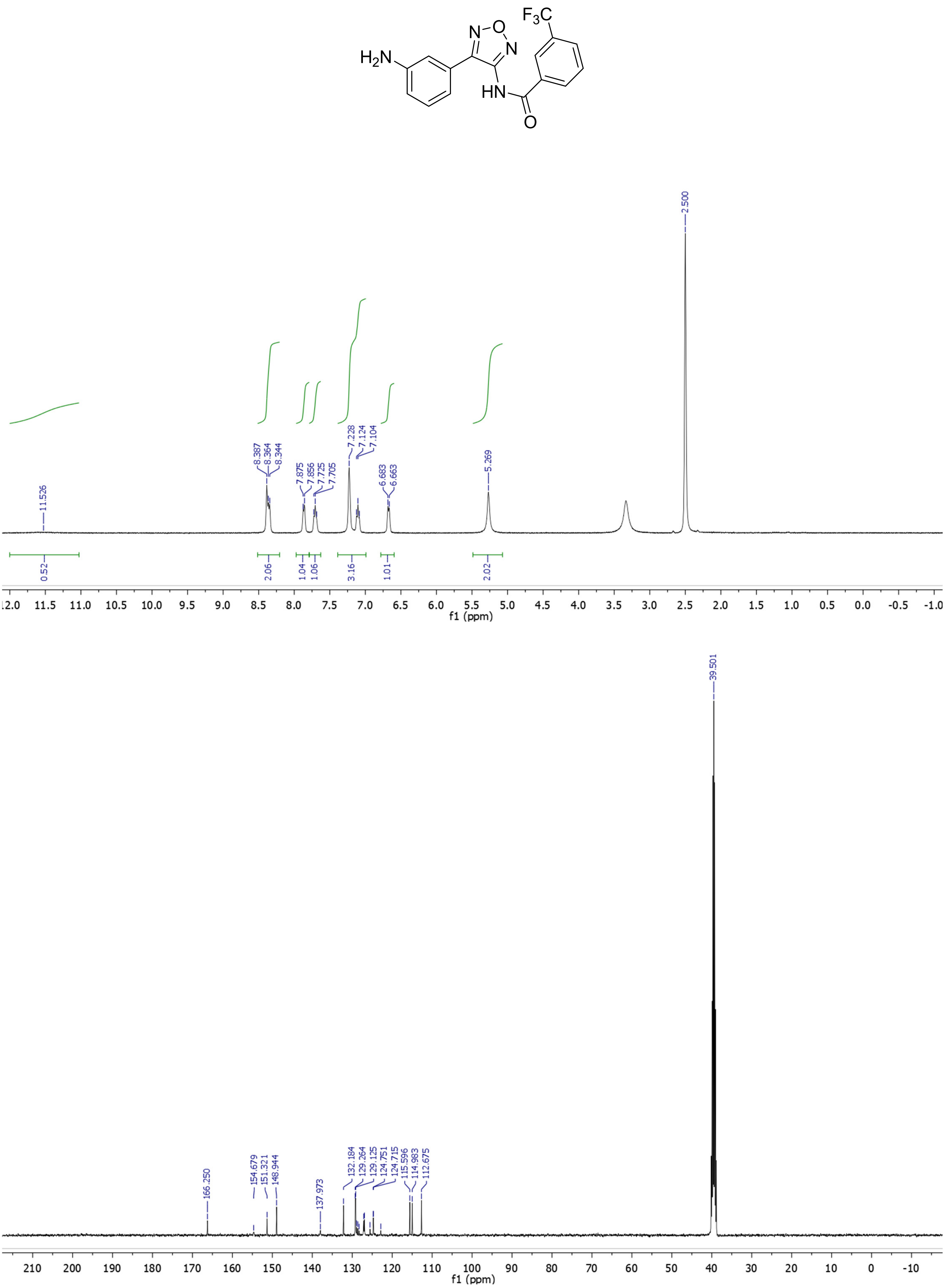

Supplement: Supplementary file 1 [file ijms-24-14480-s001.zip › ijms-2611793-supplementary.pdf]
